# Germ Warfare in a Microbial Mat Community: CRISPRs Provide Insights into the Co-Evolution of Host and Viral Genomes

**DOI:** 10.1371/journal.pone.0004169

**Published:** 2009-01-09

**Authors:** John F. Heidelberg, William C. Nelson, Thomas Schoenfeld, Devaki Bhaya

**Affiliations:** 1 Department of Biological Sciences, Marine Environmental Biology Division, Wrigley Institute for Environmental Studies, University of Southern California, Avalon, California, United States of America; 2 Lucigen Corporation, Middleton, Wisconsin, United States of America; 3 Department of Plant Biology, Carnegie Institution for Science, Stanford, California, United States of America; University of Hyderabad, India

## Abstract

CRISPR arrays and associated *cas* genes are widespread in bacteria and archaea and confer acquired resistance to viruses. To examine viral immunity in the context of naturally evolving microbial populations we analyzed genomic data from two thermophilic *Synechococcus* isolates (*Syn* OS-A and *Syn* OS-B′) as well as a prokaryotic metagenome and viral metagenome derived from microbial mats in hotsprings at Yellowstone National Park. Two distinct CRISPR types, distinguished by the repeat sequence, are found in both the *Syn* OS-A and *Syn* OS-B′ genomes. The genome of *Syn* OS-A contains a third CRISPR type with a distinct repeat sequence, which is not found in *Syn* OS-B′, but appears to be shared with other microorganisms that inhabit the mat. The CRISPR repeats identified in the microbial metagenome are highly conserved, while the spacer sequences (hereafter referred to as “viritopes” to emphasize their critical role in viral immunity) were mostly unique and had no high identity matches when searched against GenBank. Searching the viritopes against the viral metagenome, however, yielded several matches with high similarity some of which were within a gene identified as a likely viral lysozyme/lysin protein. Analysis of viral metagenome sequences corresponding to this lysozyme/lysin protein revealed several mutations all of which translate into silent or conservative mutations which are unlikely to affect protein function, but may help the virus evade the host CRISPR resistance mechanism. These results demonstrate the varied challenges presented by a natural virus population, and support the notion that the CRISPR/viritope system must be able to adapt quickly to provide host immunity. The ability of metagenomics to track population-level variation in viritope sequences allows for a culture-independent method for evaluating the fast co-evolution of host and viral genomes and its consequence on the structuring of complex microbial communities.

## Introduction

Clustered Regularly Interspaced Palindromic Repeats (CRISPRs) and related Cas (CRISPR associated) genes have been identified in several bacterial and archaeal genomes, but until recently no specific function was ascribed to them [1]–[7]. CRISPR arrays consist of multiple (2–250) direct repeats typically 21–47 base pairs (bp) with each repeat separated by variable spacer sequences [8]–[10]. CRISPRs are frequently adjacent to *cas* genes, which encode proteins with sequence similarity to components of the eukaryotic RNA interference (RNAi) system [11]–[13], and the CRISPR/Cas system has gained recent attention because they have been proposed to provide the host with acquired resistance to extrachromosomal elements (*e.g.,* viruses and plasmids) through a mechanism analogous to the RNAi system. In this model, the variable spacer sequences between the CRISPRs are transcribed and interfere with viral gene expression (possibly via targeted degradation) [2], [6], [8], [12], [14]. Because of the recent experimental support for this model (including this work), we propose that ‘spacers’ be renamed ‘viritopes’ to better describe the critical role of these viral-derived sequences in acquired resistance, as well to indicate that these sequences are specific and maybe rapidly evolving (somewhat analogous to ‘epitopes’ in proteins).

Although the potential role for the CRISPR systems in host immunity had been suggested for some time [11], direct evidence in support of their role in providing immunity against viruses has only come recently from the demonstration that well-characterized strains of *Streptococcus thermophilus*, a bacterium used for yogurt and cheese making, respond to viral predation by integrating new viritope DNA, derived from the infecting phage genome, into their CRISPR arrays [15]. Barrangou *et al*. also demonstrated that addition or removal of specific viritopes changed the phage-resistance phenotype of the bacterium, indicative of viritopes-specific resistance [15]–[17].

Very few studies have been done to examine the CRISPR/virus dynamics in naturally occurring microbial communities [18], [19]. Analysis of limited community genomic data derived from acidophilic biofilms suggested that there may have been recent lateral transfer of the CRISPR/Cas locus between populations of two distinct *Leptospirillum* group II bacteria [19]. Additionally, comparative genomics suggest that viritope sequences were subsequently lost in the population followed by acquisition of new heterogeneous viritopes. However, in the absence of a relevant viral metagenome the role and importance of the viritope sequences could only be inferred [19].

Hotspring microbial mats in the effluent channels of Octopus Spring and Mushroom Spring in Yellowstone National Park are relatively dense, simple and stable prokaryotic communities, where the uppermost green layers are dominated by obligate phototrophs (predominantly *Synechococcus* sp), while green non-sulfur-like bacteria (GNSLB) such as *Chloroflexus* sp. and *Roseiflexus* sp. are found in the lower orange pigmented layers [20], [21]. Molecular approaches, such as denaturing gradient gel electrophoresis and 16S RNA phylogenies, have been used to measure the diversity of cyanobacteria within the photic zone of the microbial mat communities. From these extensive studies has emerged the view that cyanobacterial (*Synechococcus* sp.) communities within the mats have a well-defined distribution that correlates with established environmental gradients of temperature and light availability [20], [22]. To extend and correlate these observations to relevant genomic differences between these populations we sequenced the genomes of two closely related *Synechococcus* isolates, namely *Synechococcus JA-3-3Aa* (hereafter *Syn* OS-A) which is predominantly found in the higher temperature ranges of the mat and *Synechococcus JA-2-3B′a (2-13)* (hereafter *Syn* OS-B′) which is dominant at the lower temperature ranges of the mats [23], [24]. Preliminary comparative analysis of these two genomes has suggested the presence of potentially niche adaptive genes/functions that were unique to one population [23]. In addition to these complete genome sequences, prokaryotic metagenome sequences were obtained from both the low and high temperature regions of Octopus Spring and Mushroom Spring mats [23]. Furthermore, a viral metagenome (virome) derived from Octopus Spring water is also available [25]. This provides a unique opportunity to simultaneously examine the CRISPRs and their associated viritopes in the *Synechococcus* isolates and prokaryotic metagenomic sequence database as well as to carry out a comparative analysis of these viritopes to the virome database. From these comparative analyses emerges a fascinating snapshot of ‘germ warfare’ in a natural microbial community in which we find evidence that both viruses and host populations are rapidly evolving. Furthermore, we can use relevant metagenomic information to track these dynamics over temporal and spatial scales.

## Results and Discussion

Our aim was to examine the role of CRISPR mediated viral immunity on virus/host interactions within the context of a naturally evolving mat community and to consider their role in the population dynamics within this community. We took a comparative genomic approach in which we analyzed the genomes of two thermophilic *Synechococcus* isolates, a microbial metagenome database and a virome database all collected from either Octopus or Mushroom hotsprings at Yellowstone National Park [23], [25]. By using a culture-independent approach to obtain environmental CRISPR and viritope sequences we avoided the problems commonly associated with cultivation biases [26], [27], in this case specifically alleviating the difficulties of obtaining a representative collection of individual *Synechococcus* isolates and their associated viruses in culture.

**Figure 1:**
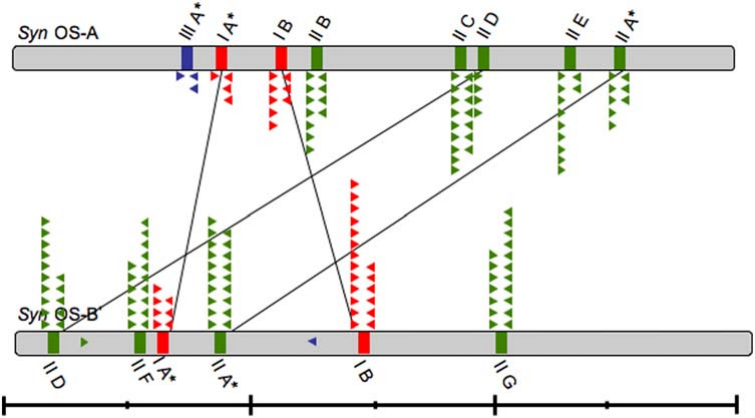
Schematic location of CRISPR loci on *Syn* OS-A OS-B genomes. Bars indicate the relative positions of the Type I (red), Type II (green) and Type III (blue) CRISPR loci on the genomes of *Syn* OS-A (top) and *Syn* OS-B′ (bottom). Asterisks indicate CRISPRs with an associated *cas* operon. CRISPRs within syntenic genome blocks are connected with lines. Approximate location and direction of the mapped clone-mates of CRISPR containing clones are show as triangles colored by the repeat type (as above). Two clones that mapped to a location on a reference genome that lacks a CRISPR array are shown within the genome box. Line below shows genome size (large ticks at 1 Mbp; small ticks at 0.5 Mbp).

CRISPR loci identified on the *Syn* OS-A and *Syn* OS-B′ genomes were categorized into three types (Type I, II, and III) based on the sequence of the repeats (**Table 1** and **Table S1**). The Type III repeat is found in a CRISPR of the *Ecoli* subtype based on the classification of Haft *et. al.* while the Type I and II are as yet untyped [9]. *Syn* OS-A contains a total of eight CRISPR arrays, two of Type I, five of Type II, and one of Type III (**Figure 1**), while *Syn* OS-B′ contains six CRISPRs, two of Type I and four of Type II. The Type III repeat sequence, which we identified in *Syn* OS-A, but not in *Syn* OS-B′ is also present in the *Roseiflexus* RS-1, a GNSLB, which is abundant in the microbial mat.

**Table 1:** CRISPR repeats in *Synechococcus* OS-A and OS-B′.

Repeat Type	# Repeats	Repeat consensus	Start Position	End Position	CRISPR_ID^a^
**OS-A**	97				
IA*	42	**RKRKSCR**T**SCYC**TTGC**R**GGGA**R**AAGGTAG**R**GATCAAC	889207	892334	NC_007775_2
IB	9	GTTTC**S**GTCCCCTTGCGGGGAAAAGGTAGGGATCAAC	1139963	1140602	NC_007775_3
IIA*	12	**S**TTTC**YR**A**Y**T**Y**CT**MRR**GATCCC**Y**C**YO**AAGGG**RD**AAC	2557478	2559420	NC_007775_9, NC_007775_10
IIB	7	GTTCCCCCTTC**S**GGGGGATCC**Y**TAGA**W**A**KK**G**KRW**A**S**	1260140	1260640	NC_007775_4
IIC	2	AGTTCCCCCTTCGTGGGGATCCCTAGAAATTGGAAAC	1860020	1860135	NC_007775_5
IID	2	GTTTCCAATTTCTAGGGATCCCCCCGAAGGGGGAAC	1960215	1960325	NC_007775_6
IIE	16	**KWK**TCCAATTTCTAGGGATCCCCC**O**GAAGGGGGAAC	2327972	2329201	NC_007775_7
III*	7	**Y**GGTTCACCCCCACGGGTGTGGGGACAAC	733236	733709	NC_007775_1
**OS-B′**	125				
IA*	9	**RKWKS**C**R**T**S**C**Y**CTTGC**R**GGGA**R**AAGGTAG**R**GATCAAC	604640	605267	NC_007776_4
IB	16	GTTTCCGTCCCCTTGCGGGGAAAAGGTAGGGATCAAC	1428062	1429246	NC_007776_8
IIA*	16	GTTTCCAATT**O**CTA**KR**GATCCCCCC**R**AAGG**RRR**AAC	866037	867185	NC_007776_5
IID	35	GT**Y**C**Y**CCCTTCGGGGGGATCCCTAGAA**R**T**Y**GGAAAC	156596	159176	NC_007776_1
IIF	18	GTTTCCAATTTCTAGGGATCCCCCCGAAGGGGGAAC	515271	516591	NC_007776_3
IIG	31	GTTCCCCCTTCGGGGGGATCCYTAGAAATTGGAAA**M**	2016367	2018657	NC_007776_9

The Table shows the CRISPR Repeat Type, number of repeats, the consensus sequence with the start and end position of the array on the genome and the CRISPR ID assigned by CRISPR db.

a From CRISPRdb [30]. All sequence symbols follow the IUPAC Nucleotide Symbol code. **G** Guanine; **A** Adenine; **T** Thymine; **C** Cytosine; **R** G or A; **Y** T or C; **M** A or C; **K** G or T; **S** G or C; **W** A or T; **H** A or C or T; **B** G or T or C; **V** G or C or A; **D** G or A or T, **N** G or A or T or C. The letter **O** represent s a gap found in one or more of the sequences.

### Type I CRISPRs

**Figure 2:**
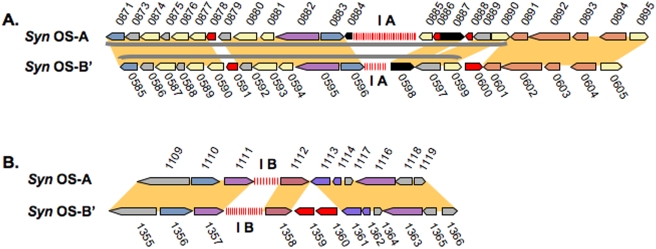
Type I CRISPR regions. A) CRISPR-IA region from *Syn* OS-A (top) and *Syn* OS-B′ (bottom). The *cas* gene cluster is indicated by a grey bracket. B) CRISPR-IB region. Gene identifiers are shown above or below each gene, excluding the GenBank locus tag prefix ‘CYA_’ (for *Syn* OS-A) or ‘CYB_’ (for *Syn* OS-B′). Genes are color-coded following the COG-based IMG convention (http://img.jgi.doe.gov), (described in Figure 4), except that genes annotated as “hypothetical protein” are in grey, genes with no putative ortholog in the other genome are in red, and genes annotated as transposases are in black. Orthologous genes are indicated by yellow blocks. Detailed information on each gene is available in Table S3.

The Type I CRISPR repeat is 37 bp and loci containing this repeat are found at two locations on both the *Syn* OS-A and *Syn* OS-B′ genomes; one CRISPR array is adjacent to the *cas* genes (IA) (CRISPR arrays with associated *cas* genes are designated with an A suffix), while the other is not (IB) (**Table 1** and **Table S1**, **Figure 1** **and** **2**). *Syn* OS-A CRISPR-IA has 42 repeats (889,207–889,366 bp), and *Syn* OS-A CRISPR-IB has 9 repeats (1,139,963–1,140,566 bp). *Syn* OS-B′ CRISPR-IA has 9 repeats (604,640–604,749 bp), and *Syn* OS-B′ CRISPR-IB has 16 repeats (1,429,210–1,428,099 bp) (**Table 1**, **Figure 1**). There is synteny between the *Syn* OS-A and *Syn* OS-B′ genomes in the regions surrounding CRISPR-IA (**Figure 2A** **)**. This is notable considering that despite being closely related organisms (average 86% nucleic acid identity (NAID) for coding genes), *Syn* OS-A and *Syn* OS-B′ have highly rearranged genomes, with the average size of co-linear genome fragments <6 kbp [23]. The syntenic region around CRISPR-IA includes the *cas* genes and a highly conserved nitrate ABC transport system (94%–98% amino acid identity (AAID); **Table S2**). However, both genomes have a few unique genes that interrupt the conserved gene order in this region (*i.e.,* CYA_0878, CYA_ 0886 (a pseudogene of a CRISPR-associated protein Cas1 with an interruption-C) and CYA_0888 (a pseudogene of a CRISPR-associated protein Cas1 with an interruption-N) and CYB_0591 and CYB_0600, indicated by red arrows in **Figure 2A**). The *cas2* gene is present in *Syn* OS-A (CYA_0885), but there is no detectable *cas2* at an analogous location in *Syn* OS-B′. The Cas2 proteins represent a novel family of endoribonucleases, and therefore might be expected to be a requirement for functional CRISPR mediated immunity [13]. Metagenome assemblies of *Syn* OS-B′-like sequences show a *cas2* gene in place of the ISSoc1 transposon (CYB_0598), suggesting that the *cas2* deletion has only occurred in a subpopulation of *Syn* OS-B′ within the community (data not shown).

The CRISPR-IB region is also syntenic between the *Syn* OS-A and *Syn* OS-B′ genomes (**Figure 2B**), suggesting that the chromosomal regions harboring the CRISPR-IA and CRISPR-IB were in the common ancestor and no substantial rearrangements have occurred within these regions since divergence of the *Syn* OS-A and *Syn* OS-B′ lineages. Note that in the CRISPR-IA region there is an ISSoc1 transposon within the *cas* cluster in both genomes with 93% AAID (CYA_0887 and CYB_0598), while in the *Syn* OS-B′ IB, two genes (CYB_1359 and 1360) disrupt the synteny although it is generally maintained across a region spanning ∼11 Kbp.

### Type II CRISPRs

**Figure 3:**
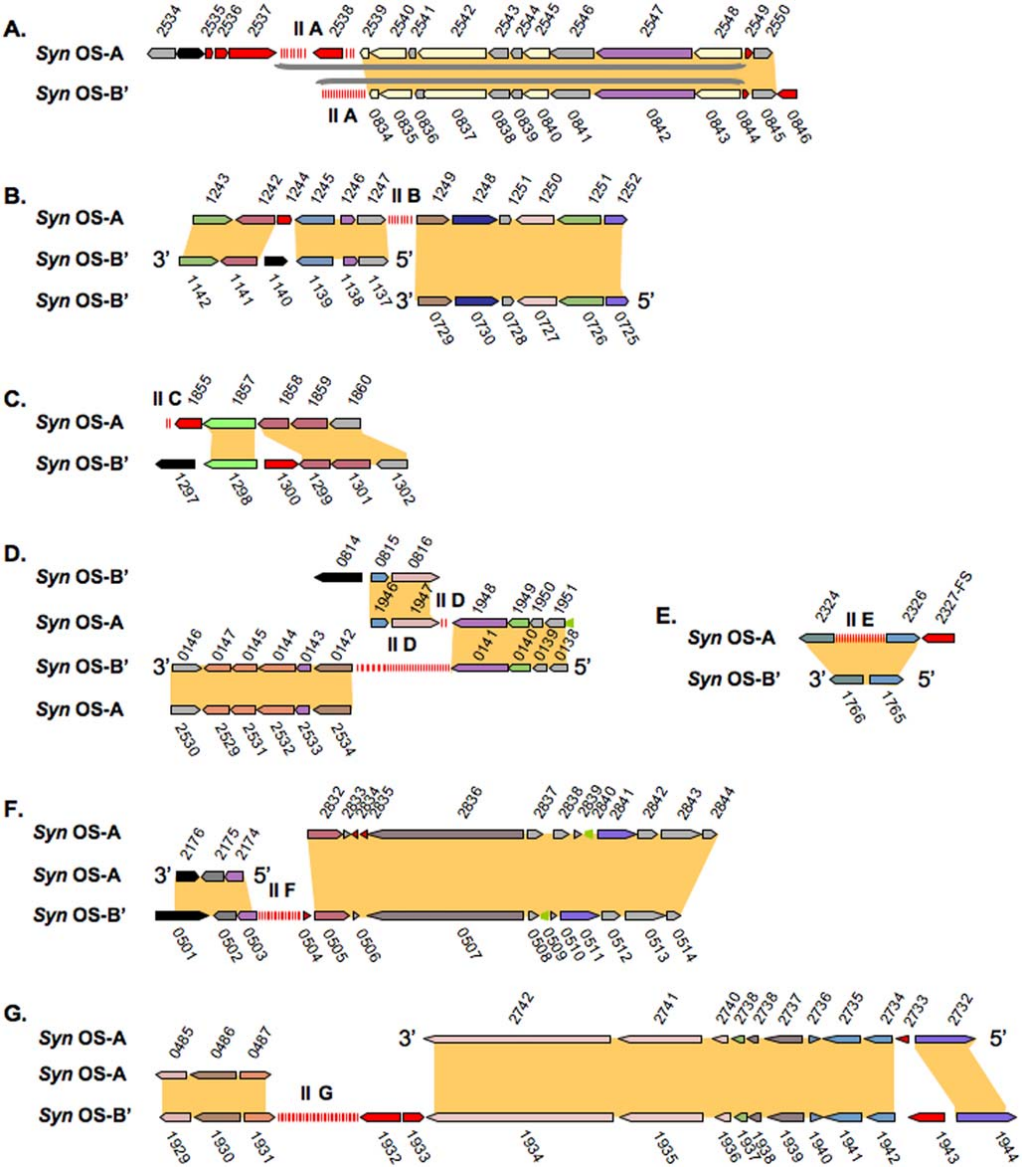
Type II CRISPR regions. Homologous regions from *Syn* OS-A and *Syn* OS-B′ where one or both genomes has a Type II CRISPR locus are displayed. The CRISPR displayed is indicated by the panel letter (*e.g.* panel ‘A’ shows CRISPR-IIA, panel ‘B’ shows CRISPR-IIB, etc.) Figure conventions are as described in Figure 2. FS indicates a frameshift in the CDS. Additional details can be found in Table S3.

The Type II CRISPR repeat is 36 bp (**Table 1** and **S1**). Type II CRISPR arrays are found at five locations on the *Syn* OS-A genome (IIA-E) and at four locations on the *Syn* OS-B′ genome (IIA, IID, IIF and IIG) (**Figure 1**). One CRISPR array on each genome (IIA) is associated with the *cas* genes while the others are not. The *Syn* OS-A CRISPR-IIA has 12 repeats associated with the *cas* genes (2,557,478–2,559,455 bp). The other locations of the Type II CRISPRs on the *Syn* OS-A genome include 7 repeats at 1,260,139–1,260,640 (IIB), two repeats at 1,860,020–1,860,135 (IIC), two repeats at 1,960,214–1,960,325 (IID), and 17 repeats at 2,327,972–2,329,201 (IIE). *Syn* OS-B′ CRISPR-IIA has 16 repeats associated with the *cas* genes (866,037–867,185 bp). The other locations of the CRISPR-II on *Syn* OS-B′ include 35 repeats spanning 156,596–159,176 bp (IID), 18 repeats spanning 515,271–516,592 (IIF), and 31 repeats spanning 2,016,367–2,018,657 (IIG) (**Table 1** and **Figure 3** **)**.

Synteny between the *Syn* OS-A and *Syn* OS-B′ genomes is maintained around the CRISPR-IIA and CRISPR-IID regions (**Figure 3A and 3D**); none of the other CRISPR-IIs are found with similar genomic context between *Syn* OS-A and *Syn* OS-B′ (**Figure 3**). The presence of identical CRISPR-II repeats and the synteny between the *Syn* OS-A and *Syn* OS-B′ genomes associated with CRISPR-IIA and IID suggests that the last common ancestor of both organisms contained at least one CRISPR-II.

### CRISPRS arrays not associated with a *cas* gene cluster

Both *Syn* OS-A and *Syn* OS-B′ genomes have multiple CRISPR arrays of which only one array of each type is associated with the *cas* genes (Type IA and Type IIA). CRISPR arrays are rarely seen in the absence of *cas* genes but such cases have been documented [11]. In most bacterial genomes there is one CRISPR array contiguous with the *cas* gene cluster [9], [11], which has led to the proposal that Cas proteins may function primarily on proximally arranged CRISPRs. It has also recently been demonstrated that the *cas* encoded enzymatic machinery is not effective in conjunction with the CRISPRs of a separate locus [15]. Currently, it is not known if the unassociated CRISPR arrays on the *Syn* OS-A and *Syn* OS-B′ genomes are active. However, the extensive variation in viritope count and content between the various CRISPRs is a possible indication that these unassociated arrays are active. Additionally, there are cases where a metagenome sequence (see below) that maps to a location on the genome contains a greater number of repeats than the analogous location in the reference genome (*e.g.,* CRISPR_II_metagenome_CYPL031TF contains 11 repeats and maps to *Syn* OS-A CRISPR-IIC where there are only 2 repeat sequences). It is unclear, when or how, the CRISPR-II arrays which are not associated with *cas* genes moved into their current locations.

### Type III (Ecoli subtype) CRISPRs

**Figure 4:**
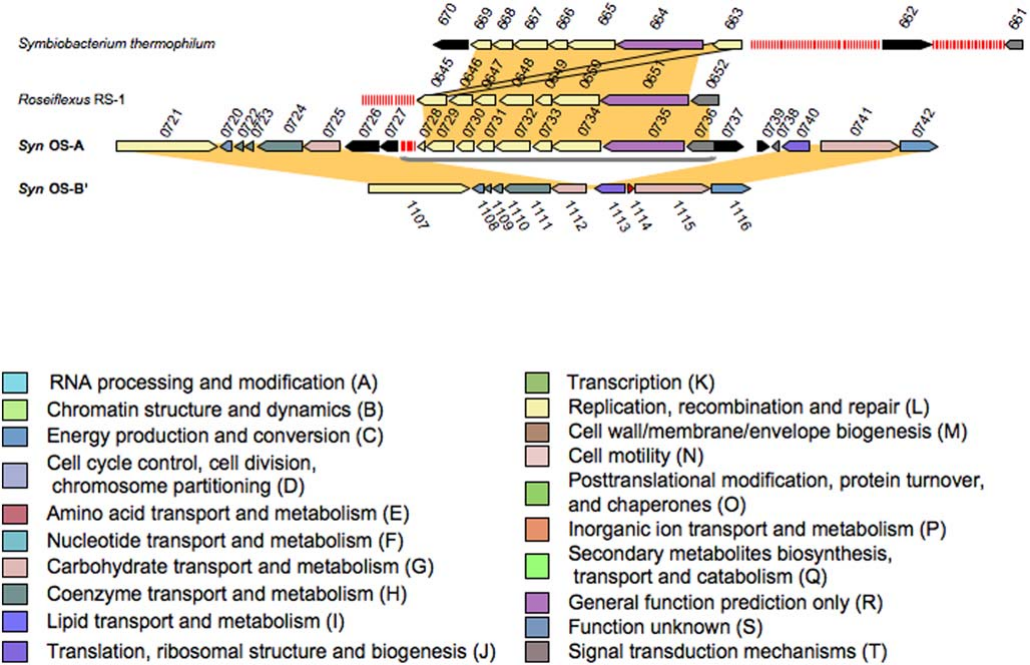
Comparison of Type III CRISPR locus to *Roseiflexus* RS-1 and *Symbiobacterium.* Homologous regions from *Syn* OS-A, *Syn*-OS-B′, *Roseiflexus* RS-1 and *Symbiobacterium* are displayed. Gene identifiers are shown above or below each gene, excluding the GenBank locus tag prefix ‘CYA_’ (for *Syn* OS-A), ‘CYB_’ (for *Syn* OS-B′), RoseRS_ (for *Roseiflexus* RS-1) or STH (for *Symbiobacterium thermophilum)*. Additional figure conventions are as described in Figure 2. Details about the genes can be found in Table S3.

*Syn* OS-A has an Ecoli subtype (as defined by Haft *et. al.* [9]) CRISPR locus, which is absent from the *Syn* OS-B′ genome. However, we found that at the syntenic location on the *Syn* OS-B′ genome there is a single Type III repeat sequence (**Figure 1**). We also found a single metagenome clone containing a Type III repeat that maps to the *Syn* OS-B′ genome rather than to the *Syn* OS-A genome based on its clone ends. Ecoli subtype CRISPR/cas loci with a nearly identical repeat are, however, present in both the *Roseiflexus* RS-1 and the *Symbiobacterium thermophilum* genomes (**Figure 4**). This is notable since the subtypes are defined by Cas protein content rather than by repeat sequence similarity [9]. *S. thermophilum* is a thermophilic bacterium originally isolated from compost and is characterized by a marked growth dependence on microbial commensalism. Phylogenetic studies indicate that *S. thermophilum* belongs to an unknown taxonomic group in the Gram-positive bacterial cluster [28]. *Roseiflexus* RS-1, which is related to *Chloroflexus* sp., is a GNSLB dominant in the higher temperature regions of the hotspring microbial mats where it is found in close proximity to *Syn* OS-A [21]. Both of these organisms have been studied mainly from an eco-physiological perspective and little is known about their CRISPR/*cas* loci or viruses that infect them. There are 8 repeats associated with this CRISPR (**Table 1** and **SI** **Table 1**) at one location (732,659–745,386 bp) on the *Syn* OS-A genome. In *Roseiflexus* RS-1 and *Symbiobacterium thermophilum* there are 18 and 86 repeats, respectively (**Figure 4**). The *cas* gene content and order are preserved between the three organisms, although the protein sequences maintain only 40%–66% amino acid similarity (AASIM) (**Table S2**). All the viritopes are unique between these organisms (data not shown), which is consistent with the notion that CRISPRs play a role in viral immunity, since different viruses likely infect these phylogenetically diverse bacterial species. It has been suggested that CRISPR loci move between different organisms by lateral gene transfer [5], [19], and it is interesting to note that the Type III/Ecoli subtype CRISPR/cas loci in both *Syn* OS-A and *S. thermophilum* are flanked by transposons (shown as black arrows) (**Figure 4**). Considering the similarity of the CRISPR repeat sequences, the absence of CRISPR III in the related *Syn* OS-B′ genome, the transposons flanking the Type III CRISPR/*cas* locus, and the co-residence of *Roseiflexus* sp. in the mat, it is likely that the Type III CRISPR/cas locus in *Syn* OS-A is the result of recent DNA transfer.

### Viritopes in the Genomes

Although the CRISPR I and II repeat sequences and their related *cas* genes exhibit high identity (**SI** **Table 1** **,** and **3**), the viritope sequences are highly variable (**Table S3**). Of a total of 208 viritopes present in *Syn* OS-A and *Syn* OS-B′, only the first viritope sequence after the *cas* gene cluster in the CRISPR-IA is shared between the genomes (at >85% NAID over 70% of the viritope sequence). Searching with these viritope sequences against GenBank using either BLASTN or TBLASTX yielded no significant hits (except to the *Syn* OS-A and *Syn* OS-B′ genomes themselves). However, it is generally acknowledged that viral genome data is under-represented in GenBank and that viral genomes are also very varied [29] so this is not an unexpected result. Thus, without further sequence coverage of both the prokaryotic and viral metagenome the implications of these results are as yet, unclear. It is possible that the *Syn* OS-A and *Syn* OS-B′ isolates, which have adapted to different niches, are attacked by different viruses, consequently one could expect little or no similarity among the viritopes (however see later section that describes some common viritopes that were identified). Another possibility is that a very large population of viritopes may be generated from a single virus genome. With the sequence coverage of the virome currently available we cannot rule out either of these possibilities.

### CRISPR arrays represented in the microbial metagenome libraries

o acquire a more comprehensive picture of CRISPR diversity within the *Syn* OS-A and *Syn* OS-B′ lineages, we searched a Yellowstone hotspring microbial metagenome library (containing a total of 202,331 sequences) for CRISPR repeats (**Table S1**). Libraries were derived from Mushroom and Octopus Spring and in both cases libraries were made from mat samples collected from “low” (∼60°C) and “high” (∼65°C) temperature regions of the effluent channel [23]. Two approaches were taken in searching the metagenome for CRISPRs. For the first search all the metagenome sequences were submitted to CRISPRFinder [30]. CRISPRFinder [30] was used for its ability to find the direct repeats, thus allowing it to identify both those CRISPRs homologous to those in the *Syn* OS-A and *Syn* OS-B′ genomes, as well as any other *bona fide* CRISPRs not present in the reference genomes. Analysis of both the CRISPR-containing sequence and the sequence from the clone-mate (all inserts in the metagenomic clone library were sequenced from both ends of the vector, thus each clone provides sequence information for two ‘clone mates’), did not yield any new CRISPRs in clones derived from a *Syn* OS-A- or *Syn* OS-B′-like organisms. Therefore, it is likely that the three CRISPR types found in *Syn* OS-A and *Syn* OS-B′ genomes are the most prevalent CRISPRs in this *Synechococcus* population. We cannot, however, rule out the presence of rare or low abundance CRISPRs in the population that may not be represented in the metagenomic library.

In the second search to identify CRISPRs, all the microbial metagenome sequences were searched against a database of all *Syn* OS-A and *Syn* OS-B′ CRISPRs using BLASTN. A total of 187 metagenomic clones identified as being *Syn* OS-A- or *Syn* OS-B′-like (based on nucleotide identity of either the sequence or its clone-mate to the genomes) were found to contain Type I (43 clones), Type II (139 clones) or Type III (5 clones) CRISPRs. The majority of these clones (180 clones) could be mapped to a CRISPR locus on either the *Syn* OS-A and *Syn* OS-B′ reference genome. However, there were two clones that mapped to locations on OS-B′ that lack a CRISPR (CYPCW50TR and CYPKN21TF). Five other clones could not be specifically mapped because they either had one mate that mapped to a transposase or the mapped positions of the clone mates were distant from each other, suggesting some sort of genome rearrangement had occurred (**Figure 1** **,** **3** **, Table S3, and Table S4**). The viritope sequences identified were searched against each other using BLASTN. Considerable diversity was observed within these viritope sequences. We identified a total of 1,323 *Syn* OS-A-/*Syn* OS-B′-like Type I, Type II or Type III (Ecoli subtype) CRISPR flanked viritopes, of which 1,069 (80%) are only identified once in the metagenome (the cutoff used for identity was set at >85% NAID over 70% of the viritope sequence). No viritope sequence was found more than 5 times (**Table S3 and S5**).

In another metagenomic study of CRISPR arrays to date, there was a log normal distribution of viritope sequences, with both a conserved core set of viritope sequences and long tail of viritope sequences that were found only once or rarely. The viritope sequences showed a distinct positional distribution pattern, with shared viritopes located at the beginning of the CRISPR array, partially shared viritopes in the middle, and unique viritopes toward the end of the CRISPR array [19]. The analysis of the Yellowstone metagenomic sequences shows that there is considerably more diversity among the viritope sequences, and there is limited evidence that viritopes near the beginning of the CRISPR array are more likely to be shared. There are only six viritopes from either *Syn* OS-A or *Syn* OS-B′ genomes represented in microbial metagenomic clones, of which five are in the first or second position within the CRISPR array, which is consistent with the observation that viritope sequences tend to be acquired at the 5′ end of the CRISPR array [16].

Examination of the location of individual viritopes within CRISPR regions in the metagenome provides two interesting insights into viritope and CRISPR function. In the first case, we found 12 examples of viritope sequences that are inserted between Type I CRISPR repeats on one clone (or genome) but between Type II CRISPR repeats on another clone (or genome) (**Table S5**). This suggests that both CRISPR types have a similar mechanism or selectivity for acquiring viritopes. If this is the case, it should be borne out by further sequence analysis of viritopes. Furthermore, it suggests functional redundancy between the Type I and Type II CRISPRs.

We also found evidence of 22 viritope sequences that are common between the *Syn* OS-A- and *Syn* OS-B′-like organisms (**Table S5**). This suggests that the same viritope can be acquired independently by both *Synechococcus* lineages or that viritopes/CRISPR segments are being exchanged between the genomic lineages. This would be advantageous if the viritopes provided immunity to (and were derived from) viruses which infect both the *Syn* OS-A- or OS-B′-like organisms. In addition, the exactness of the viritope length and sequence conservation suggests that viritope selection is precise and probably not caused by a random cleavage of viral sequences followed by insertion into the CRISPR array, or that viritope maintenance is under selective pressure and only effective viritopes are preserved within the array.

We attempted to identify if any viritope sequences were uniquely linked to a particular geographic location (*e.g.* only in DNA isolated from Mushroom or Octopus Spring samples) or to a particular temperature region of the microbial mat. While our metagenome sequence coverage is too low to carry out a statistically robust analysis of viritopes, we do find common viritope sequences in both springs and at both high and low temperatures, which are consistent with the hypothesis that there may be common viruses in these geographically close and geochemically similar springs (**Table S5**). Both springs are located in the Lower Geyser Basin of Yellowstone National Park and Mushroom Spring is located 0.5 km from the well-studied Octopus Spring and the effluent waters have a very similar composition [31], [32]. Deeper sequence coverage of the viritopes in the CRISPR loci may uncover certain viral variants that are restricted to certain microniches but at this point we are unable to discern any such specific variant populations.

### Viral metagenome (virome) from Octopus Srping

A viral metagenome (virome) was recently derived (DNA collection carried out in October, 2003) from Octopus Spring effluent channel water flowing above the mats [25]. This virome sample was collected the same month as the samples for the microbial metagenome from Mushroom Spring and thirteen months prior to the collection of the Octopus Spring metagenome sample (November, 2004). Virus enriched fractions were isolated from hotspring water and concentrated by filtration [25]. This was followed by a new linker dependent DNA amplification method and library construction [25]. A total of 21,198 sequences were generated from Octopus Spring virome and a BLASTX analysis was done to identify genes. Most of these viral sequences did not have high homology to known proteins; however, several sequences were similar to phage proteins including helicases and lysins. Additionally, similarities seen in viromes from the Octopus Spring and a different lower temperature spring in the same geyser basin support the hypothesis that there is significant overlap of viral metagenomes between hot springs in close proximity [25].

### Comparison of viritopes to a virome derived from Octopus Spring

As mentioned above, the viritope sequences yielded no significant hits against GenBank using either BLASTN or TBLASTX (except to the *Syn* OS-A and *Syn* OS-B′ genomes themselves). Upon searching the viritope sequences against the virome database (using BLASTN), we identified four distinct viritope sequences present in the microbial metagenome that were also found in the virome**.** Of these, three sequences are well conserved, while the fourth is more divergent (**Table 2**). It is important to note that the *Syn* OS-A and *Syn* OS-B′ isolates, the virome and microbial metagenome samples were not collected simultaneously (see above) and since CRISPR arrays are suspected to evolve rapidly to respond to immediate viral attack, it is not surprising that we do not see more high quality sequence matches.

**Table 2:** CRISPR viritopes with similarity to virome sequences.

gnl|ti|1647165544-RC	ACTAAGGGTCCACTCTGGT**G**CGGTATGGCACTGGT
gnl|ti|1647183951-RC	ACTAAGGGTCCACTCTGGT**G**CGGTATGGCACTGGT
gnl|ti|1647183950	ACTAAGGGTCCACTCTGGT**G**CGGTATGGCACTGGT
gnl|ti|1647178644	ACTAAGGGTCCACTCTGGT**G**CGGTATGGCACTGGT
gnl|ti|1647166765	ACTAAGGGTCCACTCTGGT**G**CGGTATGGCACTGGT
gnl|ti|1647170869	ACTAAGGGTCCACTCTGGTCCGGTATGGCACTGGT
gnl|ti|1647170868-RC	ACTAAGGGTCCACTCTGGTCCGGTATGGCACTGGT
CRISPR_II_YMIA938TF-SP-5	ACTAAGGGTCCACTCTGGTCCGGTATGGCACTGGT
CRISPR_II_YMIA938TF-SP-4	ACTAAGGGTCCACTCTGGTCCGGTATGGCACTGGT
= = = = = = = = = = =	
gnl|ti|1647165465	TT**G**GCCAGTCGTCTCAAGGAGCACGGCTGGCC**T**
CRISPR_III_CYNAC89TF-SP-5	TTAGCCAGTCGTCTCAAGGAGCACGGCTGGCCC
= = = = = = = = = = =	
gnl|ti|1647175271	AGTTTAC**T**CT**T**AAGTGGGAAGG**T**GGCTTTGTCCACCATCC
gnl|ti|1647164556	AGTTTAC**T**CT**T**AAGTGGGAAGG**T**GGCTTTGTCCACCATCC
gnl|ti|1647173811	AGTTTACCCT**T**AAGTGGGAAGG**T**GGCTTTGTCCACCATCC
gnl|ti|1647170208	AGTTTACCCT**T**AAGTGGGAAGG**T**GGCTTTGTCCACCATCC
gnl|ti|1647162994	AGTTTACCCT**T**AAGTGGGAAGG**T**GGCT**A**TGTCCACCATCC
CRISPR_II_YMBCR81TF-SP-2	AGTTTACCCTCAAGTGGGAAGGCGGCTTTGTCCACCATCC
= = = = = = = = = = =	
gnl|ti|1647168791	TCCCTT**T**A**A**T**C**G**G**GA**T**AAACAACACCCAGGTAT-GAACCTGGGTGCTG**GA**GG**GG**G**A**
gnl|ti|1647168790	**GG**CC**GCATC**T**C**G**G**GA**T**AAACAACACCCAGGTAT-GAACCTGGGTGCTG**GA**GG**GG**G**A**
CRISPR_I_CYPLU89TR-SP-2	------CATTGGGAAGAAACAACACCCAGGTAT-GAACCTGGGT------------
CRISPR_I_CYPLU89TR-SP-3	----------------AAACAACACCCAGGTAT-GAACCTGGGTGCTGTTGGTTGG
CRISPR_II_CYPBU82TR-SP-5	-----------TCCCCCACCAACACCCAGGTATTTCACCTGGGTGTTGTTT-----
CRISPR_II_YMJAO09TR-SP-3	TCCCTTCACTAGGGATAAAGAACACCCAGGTAT-GAACCTGGGT------------

Segments of virome sequence that align to viritope sequences are displayed. Bold nucleotides indicate SNPs relative to the viritope sequence. RC indicates that the sequence shown is a reverse complement of the original virome sequence.

Comparing the viritopes to the virome sequences provides an interesting snapshot of the ongoing ‘germ warfare’ between the virus and host. For example, two identical viritopes identified from the microbial metagenome database that are adjacent to each other in a CRISPR array (CRISPR_II_metagenome_YMIA938TF-SP-4 and CRISPR_II_metagenome_YMIA938TF-SP-5) have seven matches in the virome database (**Table 2**, section 1). Of these seven virome matches, two were identical to the viritope sequence, while the other five had a single nucleotide polymorphism (SNP) in which there was a C to G tranversion. The fact that there is a SNP associated with these virome sequences is consistent with the concept that mutations within the viral population may result in the ability to evade the host immunity system and warranted further exploration.

**Figure 5:**
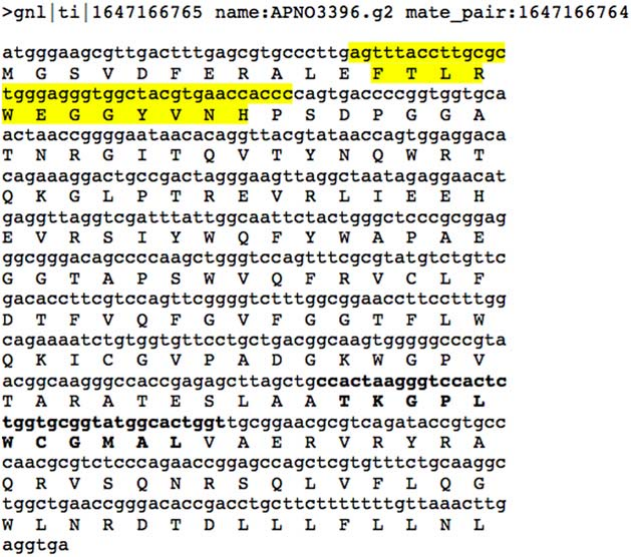
Example of a putative gene encoding a lysozyme derived from the virome. The location of the CRISPR viritopes is highlighted. The yellow highlighted region matches CRISPR_II_YMBCR81TF-SP-2 and the bold highlighted region is CRISPR_II_YMIA938TF-SP-4/5.

To further explore the potential effect of the SNP on the viral peptide sequence, we used ORFinder (NCBI) on the virome sequence read to identify the putative coding sequence (CDS) of the viral proteins from which the viritope might have been derived. This analysis revealed that three viritope sequences, CRISPR_II_metagenome_YMIA938TF-SP-4, CRISPR_II_metagenome_YMIA938TF-SP-5 (which are identical) and CRISPR_II_metagenome_YMBCR81TF-SP-2 aligned to two different locations within an open reading frame (ORF) that encodes a putative CDS (**Figure 5**). The putative CDS that contains these viritope sequences is a member of the PFAM DUF847 protein family. This family consists of several hypothetical bacterial sequences as well as one viral sequence (P5 from *Pseudomonas* phage phi8, NP_524573). While the exact function of this family is unknown, these proteins are related to lysozyme enzymes. Many phages encode a lysozyme-like protein or endolysin, which attacks the cell wall late in phage infection causing cell lysis and release of viruses. The dsDNA phages of bacteria use endolysins or muralytic enzymes in conjunction with holin, a small membrane protein, to degrade the peptidoglycan found in bacterial cell walls [33], [34]. Viral genes encoding lysozyme might be especially advantageous targets for a bacterial defense system since it might prevent or postpone lysis and thereby reduce the spread of the virus. Putative genes encoding lysozyme were abundant in the virome and, despite the noted SNPs, were highly conserved relative to other sequences both within the virome and between the virome and mesophilic phages [25]. This implies that evolution of these genes may be more constrained and slower than other phage genes. Cyanophage may use this type of lysin based on the observation that *Lyngbya* PCC 8106 a mat-forming, filamentous, non-heterocystous, nitrogen-fixing cyanobacterium contains a prophage-like island containing a probable phage-related lysozyme (ZP_01622740). However, there is no evidence of prophages in the *Syn* OS-A or *Syn* OS-B′ genomes and little is known about the phages that infect them.

The virome was searched for any additional examples of this putative DUF847 gene by using the identified CDS as a query. A total of 23 virome reads were found that contained the segment of the gene covered by viritope CRISPR_II_metagenome_YMBCR81TF-SP-2 (**Table 3** and **Figure 5**). Comparison of these sequences to CRISPR_II_metagenome_YMBCR81TF-SP-2 at both the nucleotide and amino acid level showed that while only 5 of the 23 sequences from the virome have over 90% NAID to the viritope sequence; the remaining 19 sequences *all* contain mutations that translate into silent or conservative changes in the peptide sequence such that the translation products are still 100% similar (75–100% identical) (**Table 3**). In several of these cases the nucleic acid sequence was only 70% identical and could possibly no longer be affected by the CRISPR immunity system. This finding confirms and extends the concept that the virus is co-evolving with the host and may be evading host immunity conferred by the CRISPR viritope and yet has retained infectivity. A key feature of CRISPR-mediated virus immunity is that the acquired viritope must be nearly identical to the viral genomic sequence to provide resistance [15]. Obviously we are not able to get a complete picture of this process and how it evolves since we are sampling over a short period of time, but this study emphasizes the power of using the culture-independent metagenomic approach to examine the dynamics and evolution of this process.

**Table 3:** Virome sequences showing silent or conservative changes relative to the viritope Cluster_2_YMBCR81TF-SP-2 sequence.

Sequence identifier	Nucleic Acid Sequence	%NAID	Predicated AA Sequence	% AASIM/AAID
Viritope	AGTTTACCCTCAAGTGGGAAGGCGGCTTTGTCCACCATCC		FTLKWEGGFVHH	
Y_1647173811	..........T...........T.................	95	............	100/100
Y_1647170208	..........T...........T.................	95	............	100/100
Y_1647175271	.......T..T...........T.................	92	............	100/100
Y_1647164556	.......T..T...........T.................	92	............	100/100
Y_1647162994	..........T...........T....**A**............	92	........Y...	100/91
B_1647183951-RC	........T.G**CGC**.....G..T....**AC**..G**A**....C..	70	...R....Y.N.	100/75
B_1647183950	........T.G**CGC**.....G..T....**AC**..G**A**....C..	70	...R....Y.N.	100/75
B_1647178644	........T.G**CGC**.....G..T....**AC**..G**A**....C..	70	...R....Y.N.	100/75
B_1647170869	........T.G**CGC**.....G..T....**AC**..G**A**....C..	70	...R....Y.N.	100/75
B_1647170868-RC	........T.G**CGC**.....G..T....**AC**..G**A**....C..	70	...R....Y.N.	100/75
B_1647166765	........T.G**CGC**.....G..T....**AC**..G**A**....C..	70	...R....Y.N.	100/75
B_1647165544-RC	........T.G**CGC**.....G..T....**AC**..G**A**....C..	70	...R....Y.N.	100/75
P_1647167058	....C.....G..A........G..G...........C..	86	............	100/100
P_1647166181	.*A*.................G.....G.**AC**..A**A**.**T**.....	80	........Y.N.	100/83
P_1647164597	.*A*.................G.....G.**AC**..A**A**.**T**.....	80	........Y.N.	100/83
P_1647164475	.*A*.................G.....G.**AC**..A**A**.**T**.....	80	........Y.N.	100/83
P_1647164033	.*A*.................G.....G.**AC**..A**A**.**T**.....	80	........Y.N.	100/83
P_1647163511	.*A*.................G.....G.**AC**..A**A**.**T**.....	80	........Y.N.	100/83
P_1647173178	.*C*.....A..A..A........T..T.**AC**..A**A**....C..	75	........Y.N.	100/83
P_1647166871	.*C*.....A..A..A.....G..T..G.**AC**..A**A**....C..	73	........Y.N.	100/83
P_1647162773	.*C*.....A..A..A.....G..T..G.**AC**..A**A**....C..	73	........Y.N.	100/83
P_1647183575	........T.G**CGC**.....G..T....**AC**..G**A**....C..	70	...R....Y.N.	100/75
P_1647183574	........T.G**CGC**.....G..T....**AC**..G**A**....C..	70	...R....Y.N.	100/75

**Nucleic Acid Sequences.** The viritope sequence is in Line 1. Matching sequences from the virome are shown below. Identical nucleotides are shown as a “.”. Nucleotides in regular and bold text indicate synonymous and non-synonymous changes, respectively, relative to the viritope sequence. Nucleotides in italics indicate SNPs which cannot be assigned a specific amino acid translation since the sequences have been translated in the +3 frame, thus the first base of the codon is missing. Sequence identifier prefixes indicate the method used to find them: ‘**Y**’–virome sequence with high NAID to CRISPR_II_YMBCR81TF-SP-2; **‘B**’–virome sequence with high NAID to CRISPR_II_YMIA938TF-SP-4/5; ‘**P**’–amino acid similarity to translations of ‘Y’ and ‘B’ sequences. ‘RC’ indicates that the sequence shown is the reverse complement of the database entry. NAID of the virome sequences to the viritope are shown. For the predicted amino acid (AA) sequence of the viritope and virome sequences which have been translated starting from nucleotide position #3. Percent similarity (SIM) and identity (ID) of amino acids to the translated amino acids of the viritope are shown at extreme right. Identical amino acids are shown as a “.”. Those AAs that differ from the viritope AA sequence are shown, and represent conservative changes.

### Concluding remarks

The role and importance of phage and phage resistance mechanisms in the population structure and dynamics of microbial communities is still very poorly understood although CRISPR related host immunity is currently the subject of intense interest (see Sorek *et al*, 2008 for a recent review [7]). In this study, we have gained some important insights into aspects of host/virus interactions in natural populations. CRISPRs most likely play an important role in defense against phages, however the details of the mechanism are not yet understood. The ∼1,300 viritope sequences identified in the microbial metagenome provide a catalog of viritopes from hundreds of *Syn* OS-A- or *Syn* OS-B′-like individual cyanobacterial cells (based on the assumption that since the number of metagenome clone sequences available is very small relative to the number of individuals in the population, each clone is likely to represent a DNA insert from a single individual). Interestingly, we observe very few shared viritope sequences. Even a comparison of the microbial metagenome viritope repertoire of the *Syn* OS-A- or *Syn* OS-B′-like sequences to the viral metagenome yields very few exact matches. It is important when interpreting this observation to keep in mind the time frame over which these data were collected. The cyanobacterial isolates were collected over a year prior to the DNA sampling for the virome. Likewise, the sampling of the Mushroom Spring microbial mats for metagenome characterization was carried out a year before the Octopus Spring sampling. However, even a comparison of the microbial metagenome viritope repertoire of the *Syn* OS-A- or *Syn* OS-B′-like sequences yields very few exact matches (these were isolated in the same month and from the same hotspring). This suggests that either the diversity of the phage population is so high that the CRISPR system is overwhelmed, or that the CRISPR response to viral attack is swift and very localized (perhaps to the microniche level). Another possibility is that the potential ‘viritope sequence space’ is very large, and thus, it is unlikely that the same viritope will be generated twice. For example, a virus with only a 5 kb genome could be the source of 125 non-overlapping viritopes of 40 bp; while a virus with a 150 kb genome could generate as many as 3,750 non-overlapping viritopes. If viritope acquisition is random, even a small virus population could result in the diversity of viritope sequences observed in this study. It has been estimated that there is a very large phage population in natural environments and there may be as many as 5–10 phages for every bacterial cell in an aquatic environment [35], [36]. In contrast, Octopus hot spring has a virus to microbe abundance ratio of 0.34; however its estimated 1,310 viral types greatly outnumber the microbial species diversity for the mats [25]. This suggests the dynamics between phage and bacteria in this system results in very rapid changes.

Rapid changes within the CRISPR arrays due to virus/host dynamics have been suggested based on analysis of viritope sequences identified in bacteria from an acid mine drainage ecosystem which show a high degree of variability. Moreover, virus population genomic analyses provided evidence of rampant recombination events [19]. We have not yet carried out detailed population analyses to examine recombination events in the hotspring ecosystem. Here we show that by examining both the host viritope and a viral metagenome derived from the same environment we obtain a snapshot of germ warfare in action. Since the metagenomes provide information without the need for cultivation of either the host or phage it is possible to derive information from an entire community in its natural dynamic state. Analysis of metagenomic sequences and entire genomes of cultivated microbes both have unique advantages and disadvantages. Metagenomic analysis provides a more representative sampling with less culture-bias and allows inferences about distribution and abundance of specific sequences, but necessarily results in analysis of relatively short contiguous sequences. Analysis of complete genomes allows identification of neighboring genes, gene order and gross genome structure and allows association of sequences with microbial physiology, but information about relative abundance is lost. The combination of these two sources of genomic data proved particularly powerful in understanding the dynamics of the apparent interrelation of predator and prey, in this case host and virus. In theory, with deep sequence coverage of targeted regions from host viritope and viral metagenomes one might assemble a comprehensive picture of ‘germ warfare’ in naturally evolving populations. One may also be able to trace changes over time and correlate this to changes in virus sequence, viral populations, and the populations of host bacteria.

## Methods

### Study site location and environmental genomic sequencing

Metagenomic sequences were generated from high (∼65°C) and low (∼60°C) temperature samples of the microbial mats of Octopus and Mushroom Springs, two springs with similar physicochemical characteristics that are located close to each other [37]. Total DNA was isolated from the top green layer (upper ∼1 mm) from these microbial mats. Plasmid libraries with small (2–3 kbp) and large (10–12 kbp) inserts were constructed in pUC-derived vectors following random mechanical shearing (nebulization) of genomic DNA [23]. Sequencing was performed on an ABI 3730xl (Applied Biosystems) capillary DNA sequencer at the J. Craig Venter Institute's Joint Technology Center (JTC). Detailed information on the sample location and number of sequences are in **Table S6** (modified from [23]). The *Synechococcus* isolates which were sequenced (*Syn* OS-A and *Syn* OS-B′) were from collections made from Octopus Spring in July, 2002 [22].

*Analysis of the CRISPR arrays CRISPRfinder.* The entire genome of *Syn* OS-A or *Syn* OS-B′ were submitted to the CRISPRfinder tool [30]. Genes adjacent to the CRISPR array were used to search for the ortholog in the other genome. Likewise, the entire dataset of the metagenome was submitted to CRISPRfinder 25,000 sequences at a time. All viritope and spacer sequences were copied from the CRISPRfinder into multiple fasta-formatted files. To determine the metagenome clones that were derived from *Syn* OS-A- or *Syn* OS-B′-like organisms, both end-reads were searched with BLASTN against a database of all competed microbial genomes at the Comprehensive Microbial Genome Site [38]. Only those clones were there was a hit of >80% of the read length and 90% NAID to either *Syn* OS-A or *Syn* OS-B′ were included. This would eliminate clones derived from other microbial genomes, but it would also exclude clones containing only a CRISPR array since the viritope are variable enough to be excluded from the above cut-off.

### Comparative metagenomics of the CRISPR loci

The synteny between the genomes was determined by determining putative orthologs with bi-directional best BLAST searches [39]. Briefly, the peptide sequences of the predicted proteins surrounding each CRISPR array were searched with BLASTP against the other genome. The protein with the best BLAST match (based on bit score) was then searched back against the original genome and only those proteins that had reciprocal best BLAST scores were considered as putative orthologs. The region of synteny on the genomes was found by extending the orthologs until orthologs were found elsewhere in the genome.

### Multiple alignments of the CRISPR repeat sequences

To map all variability within each CRISPR repeat type sequence, all members of each repeat type were aligned with the software program MUSCLE [40].

### Viritope similarity search

All viritopes were searched for similarity to other sequences within the Yellowstone hotspring metagenome and virome [25] and the GenBank nucleotide sequence database using BLASTN. Matches were considered significant if they had >95% NAID over 70% of the viritope). There were no significant viritope sequence matches in GenBank

## Supporting Information

Table S1 Summary of all CRISPR repeat sequences in Syn OS-A and Syn OS-B′ genomes and metagenome.
(0.11 MB DOC)

Table S2 Comparison of syntenic genes flanking the CRISPR loci
(0.05 MB XLS)

Table S3 Summary of all viritope sequences in Syn OS-A and Syn OS-B′ genomes and metagenome.
(1.46 MB DOC)

Table S4 BLASTN results of all CRISPR containing clone sequences against the Syn OS-A and Syn OS-B′ genomes.
(0.19 MB XLS)

Table S5 Viritope sequences found multiple times in the genomes and metagenomes.
(0.04 MB DOC)

Table S6 Summary of sample sites, number of CRISPR containing sequences and total number of sequences in the metagenome datasets.
(0.04 MB DOC)

## References

[1] Ishino Y, Shinagawa H, Makino K, Amemura M, Nakata A (1987). Nucleotide sequence of the iap gene, responsible for alkaline phosphatase isozyme conversion in *Escherichia coli*, and identification of the gene product.. J Bacteriol.

[2] Mojica FJ, Diez-Villasenor C, Garcia-Martinez J, Soria E (2005). Intervening sequences of regularly spaced prokaryotic repeats derive from foreign genetic elements.. J Mol Evol.

[3] Mojica FJ, Diez-Villasenor C, Soria E, Juez G (2000). Biological significance of a family of regularly spaced repeats in the genomes of Archaea, Bacteria and mitochondria.. Mol Microbiol.

[4] Mojica FJ, Ferrer C, Juez G, Rodriguez-Valera F (1995). Long stretches of short tandem repeats are present in the largest replicons of the Archaea *Haloferax mediterranei* and *Haloferax volcanii* and could be involved in replicon partitioning.. Mol Microbiol.

[5] Godde JS, Bickerton A (2006). The repetitive DNA elements called CRISPRs and their associated genes: evidence of horizontal transfer among prokaryotes.. J Mol Evol.

[6] Lillestol RK, Redder P, Garrett RA, Brugger K (2006). A putative viral defence mechanism in archaeal cells.. Archaea.

[7] Sorek R, Kunin V, Hugenholtz P (2008). CRISPR–a widespread system that provides acquired resistance against phages in bacteria and archaea.. Nat Rev Microbiol.

[8] Bolotin A, Quinquis B, Sorokin A, Ehrlich SD (2005). Clustered regularly interspaced short palindrome repeats (CRISPRs) have spacers of extrachromosomal origin.. Microbiol.

[9] Haft DH, Selengut J, Mongodin EF, Nelson KE (2005). A guild of 45 CRISPR-associated (Cas) protein families and multiple CRISPR/Cas subtypes exist in prokaryotic genomes.. PLoS computational biology.

[10] Kunin V, Sorek R, Hugenholtz P (2007). Evolutionary conservation of sequence and secondary structures in CRISPR repeats.. Genome Biol.

[11] Jansen R, Embden JD, Gaastra W, Schouls LM (2002). Identification of genes that are associated with DNA repeats in prokaryotes.. Mol Microbiol.

[12] Makarova KS, Grishin NV, Shabalina SA, Wolf YI, Koonin EV (2006). A putative RNA-interference-based immune system in prokaryotes: computational analysis of the predicted enzymatic machinery, functional analogies with eukaryotic RNAi, and hypothetical mechanisms of action.. Biol Direct.

[13] Beloglazova N, Brown G, Zimmerman MD, Proudfoot M, Makarova KS (2008). A novel family of sequence-specific endoribonucleases associated with the Clustered Regularly Interspaced Short Palindromic Repeats.. J Biol Chem.

[14] Pourcel C, Salvignol G, Vergnaud G (2005). CRISPR elements in *Yersinia pestis* acquire new repeats by preferential uptake of bacteriophage DNA, and provide additional tools for evolutionary studies.. Microbiol.

[15] Barrangou R, Fremaux C, Deveau H, Richards M, Boyaval P (2007). CRISPR provides acquired resistance against viruses in prokaryotes.. Science.

[16] Horvath P, Romero DA, Coute-Monvoisin AC, Richards M, Deveau H (2008). Diversity, activity, and evolution of CRISPR loci in *Streptococcus thermophilus*.. J Bacteriol.

[17] Deveau H, Barrangou R, Garneau JE, Labonte J, Fremaux C (2008). Phage response to CRISPR-encoded resistance in *Streptococcus thermophilus*.. J Bacteriol.

[18] Andersson AF, Banfield JF (2008). Virus population dynamics and acquired virus resistance in natural microbial communities.. Science.

[19] Tyson GW, Banfield JF (2008). Rapidly evolving CRISPRs implicated in acquired resistance of microorganisms to viruses.. Environ Microbiol.

[20] Ward DM, Ferris MJ, Nold SC, Bateson MM (1998). A natural view of microbial biodiversity within hot spring cyanobacterial mat communities.. Microbiol Mol Biol Rev.

[21] Nubel U, Bateson MM, Vandieken V, Wieland A, Kuhl M (2002). Microscopic examination of distribution and phenotypic properties of phylogenetically diverse *Chloroflexaceae*-related bacteria in hot spring microbial mats.. Appl Environ Microbiol.

[22] Allewalt JP, Bateson MM, Revsbech NP, Slack K, Ward DM (2006). Effect of temperature and light on growth of and photosynthesis by *Synechococcus* isolates typical of those predominating in the octopus spring microbial mat community of Yellowstone National Park.. Appl Environ Microbiol.

[23] Bhaya D, Grossman AR, Steunou AS, Khuri N, Cohan FM (2007). Population level functional diversity in a microbial community revealed by comparative genomic and metagenomic analyses.. ISME J.

[24] Ward DM (2006). Microbial diversity in natural environments: focusing on fundamental questions.. Antonie van Leeuwenhoek.

[25] Schoenfeld T, Patterson M, Richardson PM, Wommack KE, Young M (2008). Assembly of Viral Metagenomes from Yellowstone Hot Springs.. Appl Environ Microbiol Epub ahead of print.

[26] Rappe MS, Giovannoni SJ (2003). The uncultured microbial majority.. Annu Rev Microbiol.

[27] Snyder JC, Spuhler J, Wiedenheft B, Roberto FF, Douglas T (2004). Effects of culturing on the population structure of a hyperthermophilic virus.. Microb Ecol.

[28] Ueda K, Yamashita A, Ishikawa J, Shimada M, Watsuji TO (2004). Genome sequence of *Symbiobacterium thermophilum*, an uncultivable bacterium that depends on microbial commensalism.. Nucleic Acids Res.

[29] Ackermann HW, Kropinski AM (2007). Curated list of prokaryote viruses with fully sequenced genomes.. Res Microbiol.

[30] Grissa I, Vergnaud G, Pourcel C (2007). CRISPRFinder: a web tool to identify clustered regularly interspaced short palindromic repeats.. Nucleic Acids Res.

[31] Ramsing NB, Ferris MJ, Ward DM (2000). Highly ordered vertical structure of *Synechococcus* populations within the one-millimeter-thick photic zone of a hot spring cyanobacterial mat.. Appl Environ Microbiol.

[32] Brock TD (1978). Thermophilic microorganisms and life at high temperatures.

[33] Loessner MJ (2005). Bacteriophage endolysins–current state of research and applications.. Curr Opin Microbiol.

[34] Fischetti VA (2005). Bacteriophage lytic enzymes: novel anti-infectives.. Trends Microbiol.

[35] Breitbart M, Rohwer F (2005). Here a virus, there a virus, everywhere the same virus?. Trends Microbiol.

[36] Wommack KE, Colwell RR (2000). Virioplankton: viruses in aquatic ecosystems.. Microbiol Mol Biol Rev.

[37] Papke RT, Ramsing NB, Bateson MM, Ward DM (2003). Geographical isolation in hot spring cyanobacteria.. Environm Microbiol.

[38] Peterson JD, Umayam LA, Dickinson T, Hickey EK, White O (2001). The Comprehensive Microbial Resource.. Nucleic acids research.

[39] Altschul SF, Gish W, Miller W, Myers EW, Lipman DJ (1990). Basic local alignment search tool.. J Mol Biol.

[40] Edgar RC (2004). MUSCLE: multiple sequence alignment with high accuracy and high throughput.. Nucleic Acids Res.

