# Supplementary material for: Germ Warfare in a Microbial Mat Community: CRISPRs Provide Insights into the Co-Evolution of Host and Viral Genomes
Source: PLoS One. 2009 Jan 9;4(1):e4169. doi: 10.1371/journal.pone.0004169 (PMC2612747; doi:10.1371/journal.pone.0004169)
Supplement: Table S1 — Summary of all CRISPR repeat sequences in Syn OS-A and Syn OS-B′ genomes and metagenome. (0.11 MB DOC) [file pone.0004169.s001.doc]

**Table S1.** CRISPR repeat sequences in the genome and metagenome. Nucleotides in bold designate those that are different from the dominant Type I, II, or III repeat sequences. “N” indicates an unknown base from a metagenome clone that has a truncated sequence at either the beginning or end of the sequence read. Numbers of repeat found in the genome or metagenome are indicated at right.

**CRISPR-I Repeat sequence**

| GTTTCCGTCCCCTTGCGGGGAAAAGGTAGGGA-TCAAC | 412 copies in OS-A, OS-B', and Metagenome |
| --- | --- |
| GTTTCCGTCCCCTTGCGGGGAAAAGGTAGGGA-TCAA**T** | 3 copies in Metagenome |
| **AGAGG**C**A**T**G**C**T**CTTGC**A**GGGA**G**AAGGTAG**A**GA-TCAAC | 3 copies in OS-A, OS-B', and Metagenome |
| **T**T**G**T**TGAG**CC**T**CTTGC**A**GGGA**G**AAGGTAG**A**GA-TCAAC | 2 copies in Metagenome |
| GTTTCCGTCCCCTT**T**CGGGGAAAAGGTAGGGA-TCAAC | 2 copies in Metagenome |
| GTTTCCGTCCCCTTGCGGGGAAAAGGTAGGGA-T**T**AAC | 2 copies in Metagenome |
| GTTTC**T**GTCCCCTTGCGGGGAAAAGGTAGGGA-TCAAC | 1 copy in Metagenome |
| GTTTC**G**GTCCCCTTGCGGGGAAAAGGTAGGGA-TCAAC | 1 copy in OS-A |
| GTTTCCGTCCCCTTG**G**GGGGAAAAGGTAGGGA-TCAAC | 1 copy in Metagenome |
| GTTTCCGTCCCCTTGCGGGGA**G**AAGGTAGGGA-TCAAC | 1 copy in Metagenome |
| GTTTCCGTCCCCTTGCGGGGAAAAGGTAGGGA**A**TCAA**-** | 1 copy in Metagenome |
| GTTTCCGTCCCCTTGCGGGGAAA**-**GGTAGGGA-TCAAC | 1 copy in Metagenome |

**CRISPR-II Repeat sequence.**

| GTTCCCCCTTC-GGGGGGA-TCCCTAG-AAATTGGAAAC | 1,011 copies in OS-A, OS-B', and Metagenome |
| --- | --- |
| GTTCCCCCTTC-GGGGGGA-TCCC**C**AG-AAATTGGAAAC | 30 copies in Metagenome |
| GTTCCCCCTTC-GGGGGGA-T**T**CCTAG-AAATTGGAAAC | 26 copies in Metagenome |
| GTTCCCCCTTC-G**T**GGGGA-TCCCTA**A**-AAATTGGAAAC | 12 copies in Metagenome |
| GTT**-**CCCCTTC-GGGGGGA-TCCCTAG-AAATTGGAAAC | 11 copies in Metagenome |
| GTTCCCCCTTC-GGGGGGA-T**G**CCTAG-AAATTGGAAAC | 10 copies in Metagenome |
| GTTCCCCCTT**T**-G**T**GGGGA-TCCCTAG-AAATTGGAAAC | 9 copies in Metagenome |
| GTTCCCCCTTC-G**T**GGGGA-TCCCTAG-AAATTGGAAAC | 9 copies in OS-A and Metagenome |
| GTTCCCCCTTC-GGGGGGA-TCCCTAG-AAATTG**A**AAAC | 6 copies in Metagenome |
| GTT**TTT**CCTTC-GGGGGGA-TC**TA**TAG--AATTGGAAAC | 5 copies in Metagenome |
| GTTCCCCCTTC-GGGGGGA-TCCCTAG-AAATTGGA**CTA** | 5 copies in OS-A and Metagenome |
| GTTCCCCCTTC-CGGGGGA-TCCCTAG-A**T**A**GG**G**TGTAG** | 5 copies in OS-A and Metagenome |
| GTTCCCCCTTC-GGGGGGA-TCCCTAG-AAATTGGAA**G**C | 4 copies in Metagenome |
| GTT**-**CCCCTTC-**-**GGGGGA-TCCCTAG-AAATTGGAAAC | 4 copies in Metagenome |
| GTT**TT**CCCTTC-GGGGGGA-TC**TTG**AG-AA**G**T**CA**GAAAG | 3 copies in OS-A and Metagenome |
| GTTCC**T**CCTTC-GGGGGGA-TCCCTAG-AAATTGGAAAC | 3 copies in Metagenome |
| GTTCCC**T**CTTC-GGGGGGA-TCCCTAG-AAATTGGAAAC | 3 copies in Metagenome |
| GTTCCCCCTT**TC**GGGGGGA-TCCCTAG-AAATTGGAAAC | 3 copies in Metagenome |
| GTTCCCCCTT**T**-GGGGGGA-TCCCTAG-AAATTGGAAAC | 3 copies in OS-B' and Metagenome |
| GTTCCCCCTTC-GGGGGGA-TCCCTAG-**G**AATTGGAAAC | 3 copies in OS-A and Metagenome |
| GTTCCCCCTTC-GGGGGGA-TCCCTAG-AAATTGGAA**-G** | 3 copies in Metagenome |
| GTTCCCCCTTC-GGGGGGA-TCCCTAG-AAATTGGAA**-C** | 3 copies in Metagenome |
| GTTCCCCCTTC-GGGGGGA-TCCCTAG-AAATTGGAAA**T** | 3 copies in Metagenome |
| GTTCCCCCTTC-GGGGGGA-TCCCTAG-AAATTGGAAA**A** | 3 copies in Metagenome |
| GTTCCCCCTTC-GGG**A**GGA-TCCCTAG-AAATTGGAAAC | 3 copies in Metagenome |
| GTTCCCCCTTC**G**GGGGGGA-TCCCTAG-AAATTGGAAA**-** | 2 copies in Metagenome |
| GTTCCCCCTTC-G**T**GGGGA-T**T**CCTAG-AAATTGGAAAC | 2 copies in Metagenome |
| GTTCCCCCTTC-GGGGGGA-TC**T**CTAG-AAATTGGA**CTA** | 2 copies in Metagenome |
| GTTCCCCCTTC-GGGGGGA-TCC**T**TAG-AAATTGGAAAC | 2 copies in OS-A and Metagenome |
| GTTCCCCCTTC-GGGGGGA-TCCCTAG-AAATTGGAAA**G** | 2 copies in Metagenome |
| GTTCCCCCTTC-GGGGGGA-TCCCTAG-AAATTGGAAA**-** | 2 copies in Metagenome |
| GTTCCCCCTTC-GGGGGGA-TCCCTAG**A**AAATT**A**G**-**AA**G** | 2 copies in Metagenome |
| GTTCCCCCTTC-GGGGGGA-TCCCTAG-AAA**-**TGGAAAC | 2 copies in Metagenome |
| GTTCCCCCTTC-GGGGGGA-TCCCTAG-**-**AATTGGAAAC | 2 copies in Metagenome |
| GTTCCCCCTTC-GG**A**GGGA-TCCCTAG-AAATTGGAAAC | 2 copies in Metagenome |
| GTTCCCCCTTC-GG**A**GGGA-TCCCTAG-AAATT**A**GAAAC | 2 copies in Metagenome |
| GTT**AT**CCCTTC-**A**G**A**GGGA-GCCCTAG-AAATTGGAAAC | 2 copies in Metagenome |
| G**C**TC**T**C**T**CTTC-**T**GGGGGA-TCCCTAG-AAATTGGAAAC | 2 copies in Metagenome |
| **A**T**AT**C**TATGG**C-GGGGGGA-TCCCTAG-**G**AATTGGAAAC | 2 copies in Metagenome |
| **N**TTCCCCCTTC-GGGGGGA**T**TCCCTAG-AAATT**C**G**G**AA**A** | 1 copy in Metagenome |
| **NNNNNNNN**TTC-GGGGGGA-TCCCTAG-AAATTGGAAAC | 1 copy in Metagenome |
| **NNN**CCCCCTTC-GGGGGGA-TCCCTAG-A**G**ATTGGAAAC | 1 copy in Metagenome |
| GTT**TTT**CCTTC**G**GGGGGGA-TC**TA**TAG-**-**AATTGGAAAC | 1 copy in Metagenome |
| GTT**T**C**T**CCTTC-GGGGGGA-TC**T**CTAG-AAATTGGAAAC | 1 copy in Metagenome |
| GTT**T**CCCCTTC-**-**GGGGGA-TCCCTAG-AAATTGGAAAC | 1 copy in Metagenome |
| GTTC**T**CCCTTC-GGGGGGA-TCCCTAG-AAATTGGAAAC | 1 copy in OS-B' |
| GTTC**T**CCCTTC-GGGGG**A**A-TCCCTAG-AAATTGGAAAC | 1 copy in Metagenome |
| GTTCCCC**G**TTC-GGGGGGA-TCCCTAG-AAATTGGAAAC | 1 copy in Metagenome |
| GTTCCCCCTTC-G**T**GGGGA-TCCCT**G**G-A**C**ATTGGAAA**T** | 1 copy in Metagenome |
| GTTCCCCCTTC-GGGGGGA-TC**T**CTAG-AAATTGGAAAC | 1 copy in Metagenome |
| GTTCCCCCTTC-GGGGGGA-TCC**T**TAG-AAATTGGAAA**A** | 1 copy in OS-B' |
| GTTCCCCCTTC-GGGGGGA-TCCCTAG-AA**G**T**C**GGAAAC | 1 copy in OS-B' |
| GTTCCCCCTTC-GGGGGGA-TCCCTAG-AAATTGG**G**AAC | 1 copy in Metagenome |
| GTTCCCCCTTC-GGGGGGA-TCCCTAG-AAATTGG**G**AA**A** | 1 copy in Metagenome |
| GTTCCCCCTTC-GGGGGGA-TCCCTAG-AAATTGGAA**TT** | 1 copy in Metagenome |
| GTTCCCCCTTC-GGGGGGA-TCCCTAG-AAATT**A**GAAAC | 1 copy in Metagenome |
| GTTCCCCCTTC-GGGGGGA-TCCCTAG-AAA**NNNNNNNN** | 1 copy in Metagenome |
| GTTCCCCCTTC-GGGGGGA-TCC**A**TAG-AAATTGGAAAC | 1 copy in Metagenome |
| GTTCCCCCTTC-GGGGGGA-**C**C**T**CTAG-AAATTGGAAAC | 1 copy in Metagenome |
| GTTCCCCCTTC-GGGGGGA-**-**CCCTAG-AAATTGGAAG**-** | 1 copy in Metagenome |
| GTTCCCCCTTC-GGG**A**GGA-TCCCTAG-AAA**C**TGGAAAC | 1 copy in Metagenome |
| GTTCCCCCTTC-CGGGGGA-TCCCTAG-A**T**A**-**GG**TGTAG** | 1 copy in Metagenome |
| GTTCCCCCTTC-**C**GGGGGA-TCCCTAG-AAATTGGAAAC | 1 copy in Metagenome |
| GTTCCCCCTTC-**-**GGGGGA-TCCCTAG-AAATTGGAAAC | 1 copy in OS-A |
| GTTCCCCCT**G**C-GGGGGGA-TCCCTAG-AAATTGGAAAC | 1 copy in Metagenome |
| GTTCCCCC**CTTC**GGGGGGA**A**TCCCTAG**A**AAATTGGAAAC | 1 copy in Metagenome |
| GTTCC**-**CCTTC-**-**GGGGGA-TCCCTAG-AAA**-**TGGAAAC | 1 copy in Metagenome |
| GTTC**A**CCCTTC-GGGGGGA-TCCCTAG-AAATTGGAAAC | 1 copy in Metagenome |
| GTT**AT**CCCTTC-**A**G**A**GGGA-TCCCTAG-AAATTGGAAAC | 1 copy in Metagenome |
| GTT**AT**CCCTT**-**-**A**G**A**GGGA-TCCCTAG-AAATTGGAAAC | 1 copy in OS-A |
| GTT**-**CCCCTTC-G**T**GGGGA-TCCCTAG-AAATTGGAAAC | 1 copy in Metagenome |
| GTT**-**CCCCTTC-GGGGGGA-T**T**CCTAG-AAATTGGAAAC | 1 copy in Metagenome |
| GTT**-**CCCCTTC-GGGGGGA-TCCCTAG-AAATTGGAA**CA** | 1 copy in Metagenome |
| GTT**-**CCCCTTC-GGGGGGA-TCCCTAG-AAAT**-**GGAAAC | 1 copy in Metagenome |
| GT**C**CCCCCTT**T**-G**T**GGGGA-TCCCTAG-AAATTGGAAAC | 1 copy in Metagenome |
| GT**C**CCCCCTTC-GGGGGGA-TCCCTAG-AAATTGGAAAC | 1 copy in OS-B' |
| G**G**TCCCCCTTC-GGGGGGA-TCCCTAG-AAATTGGAAAC | 1 copy in Metagenome |
| **C**TTCCCCCTTC-GGGGGGA-TCCCTAG-AAATTGGAAAC | 1 copy in Metagenome |
| **C**T**GT**CCCCTTC-**-**GGGGGA-TCCCTAG-AAATTGGAAAC | 1 copy in Metagenome |
| **A**TT**-**CCCCTTC-GGGGGGA-TCCCTAG-AAATTGGAAAC | 1 copy in Metagenome |

**CRISPR-III Repeat sequence**

| CGGTTCACCCCCACGGGTGTGGGGACAAC | 26 copies in OS-A and Metagenome |
| --- | --- |
| **T**GGTTCACCCCCACGGGTGTGGGGACAAC | 7 copies in OS-A and Metagenome |
| CGGTTCACCCCCACG**T**GTGTGGGGACAAC | 11 copies in Metagenome |
| **G**GGTTCACCCCCACG**T**GTGTGGGGACAAC | 1 copy in Metagenome |
| **T**GGTTCACCCCCACG**T**GTGTGGGGACAAC | 1 copy in Metagenome |
| C**C**G**GCT**AC**T**CCCAC**A**G**A**TGTGGGGACAAC | 2 copies in Metagenome |
| **T**GGTTCACCCCCACGGG**C**GTGGGGACAA**T** | 1 copy in *Roseiflexus* RS1 |
| CGGTTCACCCCCACGGG**C**GTGGGGACAA**T** | 18 copies in *Roseiflexus* RS1 |
| CGGTTCACCCCCACG**C**G**C**GTGGGGAC**G**AC | 81 copies in *S. thermophilum* |
| CGGTTCACCCCCACG**C**G**C**GTGGG**A**AC**G**AC | 1 copy in *S. thermophilum* |
| **T**GGTTCACCCCCACG**C**G**C**GTGGGGAC**G**AC | 3 copies in *S. thermophilum* |
| **G**GG**CGT**ACCCCCACG**C**G**C**GTGGGGAC**G**AC | 1 copy in *S. thermophilum* |
