# Supplementary material for: Germ Warfare in a Microbial Mat Community: CRISPRs Provide Insights into the Co-Evolution of Host and Viral Genomes
Source: PLoS One. 2009 Jan 9;4(1):e4169. doi: 10.1371/journal.pone.0004169 (PMC2612747; doi:10.1371/journal.pone.0004169)
Supplement: Table S3 — Summary of all viritope sequences in Syn OS-A and Syn OS-B′ genomes and metagenome. (1.46 MB DOC) [file pone.0004169.s003.doc]

**Table S2. All Viritope Sequences identified in the Syn OS-A and OS-B' genomes and in the metagenome.** Nomenclature is based on CRISPR repeat type (I, II, or III), genome (OS-A or OS-B') or metagenome (with metagenome identifier) and VT refers to viritope with the number indicating spacer #

| >CRISPR_IA_OS-A-VT-1 |
| --- |
| CTCACTTCTCTTTGCTCATTACTCGCTTCTCTCTA |
| >CRISPR_IA_OS-A-VT-2 |
| GTTACTCACTGTAGAACACGAATTGCGTGATGAAAACT |
| >CRISPR_IA_OS-A-VT-3 |
| CATAAACCAAGTATGCAGCGTAGTCTAGATACTGTTCA |
| >CRISPR_IA_OS-A-VT-4 |
| AGTAGAGCAGGATGGCGGCAAATTCGAGAAATGTGTGAA |
| >CRISPR_IA_OS-A-VT-5 |
| TCTAACTAGAGTAATACCTGGGTTTAACTGTGAACCAGGAGA |
| >CRISPR_IA_OS-A-VT-6 |
| GACTGGGCAGTGGCATGCCTCCTGAACTAACCTAAC |
| >CRISPR_IA_OS-A-VT-7 |
| GCGGATTTCATGTTGTGCCCGACAGTAGCCTTACTTT |
| >CRISPR_IA_OS-A-VT-8 |
| ACGATCCAGTGGGGACGTGGACGCTGGACCATTAACTG |
| >CRISPR_IA_OS-A-VT-9 |
| GGCAAAGGTATTTACTCCTTAGACTAAGTAAAAGGGCA |
| >CRISPR_IA_OS-A-VT-10 |
| CGGACAAAATTGTCAAAGGACAAGCCAAGACTCTGAAGC |
| >CRISPR_IA_OS-A-VT-11 |
| GGGTGGATTCCGACTGAAACCGGTTTGGCTACCCGAGT |
| >CRISPR_IA_OS-A-VT-12 |
| GGGGCACAGATGTCGTGTTCTACGATCTGGAAACAGTCGTAGA |
| >CRISPR_IA_OS-A-VT-13 |
| AACAAAGGCCTTCGTGGTATGGTTAAACTTCGCA |
| >CRISPR_IA_OS-A-VT-14 |
| TGTAGATAAACCGGACACCGCTTTTGGTGCGTTGGA |
| >CRISPR_IA_OS-A-VT-15 |
| AACGGGTTCTCCCTCAAGGAGGCGCCCGCCATACTGAGAG |
| >CRISPR_IA_OS-A-VT-16 |
| TTTTTAGAGGGGCAGAGTTTGTATAGACTGCCTAGGAGG |
| >CRISPR_IA_OS-A-VT-17 |
| AACCATCGCTGGATCGGGGTTAATTCCCTCACTAATGC |
| >CRISPR_IA_OS-A-VT-18 |
| GGGGCTAACCAAACTGGGTTAGCCTAGATCATCTGCAAAA |
| >CRISPR_IA_OS-A-VT-19 |
| GAGGGCCGTCTCCTGACTGCCGGAACCTTCTTGGTGA |
| >CRISPR_IA_OS-A-VT-20 |
| AGTAATCTGAGGGTTGGGACAAGAGGTTTTATATAACTCAG |
| >CRISPR_IA_OS-A-VT-21 |
| TTCCAGTAGTAGTTGCGACCCCCGATTTCCCTCACT |
| >CRISPR_IA_OS-A-VT-22 |
| AAATTTATCTTTAAAGACCCGAACGAGTATATGTTCT |
| >CRISPR_IA_OS-A-VT-23 |
| CTAACCTAGATCTCGATATCACAAGTGAGGTAGAAC |
| >CRISPR_IA_OS-A-VT-24 |
| CTACCTATACGGTAGTAAAAACCTCTACATCCGTCACTGCTA |
| >CRISPR_IA_OS-A-VT-25 |
| TGTGGACTAGTGACGGTGTTGGCGGTCGCAAGCTAACCTTC |
| >CRISPR_IA_OS-A-VT-26 |
| TCGGCTAGAAACCGAGTACAAAGCCGCACTTGCAAAACA |
| >CRISPR_IA_OS-A-VT-27 |
| GTTCGAACCACCAACTTGGGTGGTTCGACAATAACTT |
| >CRISPR_IA_OS-A-VT-28 |
| TTGTTATGCAACTACTGGAACCGGTGTTCACCGTCATT |
| >CRISPR_IA_OS-A-VT-29 |
| TCCATGTTGTTGATTAACCACATGACCTCATTTTGGGTC |
| >CRISPR_IA_OS-A-VT-30 |
| GTTAAGAATGCCCTAAAGTTGTCCAGTGATCAACTGGC |
| >CRISPR_IA_OS-A-VT-31 |
| AGTGAAAACCAGGATCCAAAAGGCGAAAGCCTGCGGGTTCT |
| >CRISPR_IA_OS-A-VT-32 |
| GAATGTCAGTCTATACGATGAATGGAAAGACACTCAAG |
| >CRISPR_IA_OS-A-VT-33 |
| GCCATTGAGCGGCAGCGGGCTGAACAACAAGCCCGTGTGGAGAA |
| >CRISPR_IA_OS-A-VT-34 |
| TTGCTAAGCAACGAATAAACCTCCCCCACCCACACA |
| >CRISPR_IA_OS-A-VT-35 |
| GTGAGACACAATCGCTGCAGCCTGTGGGTTCAACTTCAT |
| >CRISPR_IA_OS-A-VT-36 |
| ACCGTTTTATTATAGCCCACGGTGCCGGGGCTCCGCAAGAC |
| >CRISPR_IA_OS-A-VT-37 |
| TCGAAGGCCAATTTCGTAGAGTACACGCCGAAAGGC |
| >CRISPR_IA_OS-A-VT-38 |
| TGTTGGGGGAGGGACTACCTTAGAGGGTTCCCCCCATCAC |
| >CRISPR_IA_OS-A-VT-39 |
| CTGGAAATTCTCGCTACTGCAGGGCGAGAAAGAAGC |
| >CRISPR_IA_OS-A-VT-40 |
| AAGACCTCCCAGATCGCCGTTGCAGTGCGCTCCAAA |
| >CRISPR_IA_OS-A-VT-41 |
| CTATTATAATCCGTATCACAAGGGGGATTTTATTCAGT |
| >CRISPR_IB_OS-A-VT-1 |
| CTGTGCAAGCAGCCATTCGGGGGTCCGTGCTCCGAGAGCT |
| >CRISPR_IB_OS-A-VT-2 |
| AACGGTTGGCTAATCCGGGAAACCGGATGGGAAATAGG |
| >CRISPR_IB_OS-A-VT-3 |
| CAAATCCAGAACCAGGTGCTTATAGCACTTGTAAAGG |
| >CRISPR_IB_OS-A-VT-4 |
| GGCGAAAGCCTGCGGATTCCGCTTGCTCCCCGCGTAC |
| >CRISPR_IB_OS-A-VT-5 |
| CTGTAATAAAACTCGGGAAGCGGCTCCGACTATACCGGG |
| >CRISPR_IB_OS-A-VT-6 |
| TCGGGGCACCCCCTCTGGAAATGTAGATGCCAGTGTGG |
| >CRISPR_IB_OS-A-VT-7 |
| TCATCGACGACTAACTTTAAGGAAGGCTACCTTATTGGT |
| >CRISPR_IB_OS-A-VT-8 |
| TCATCGACGACTAACTTTAAGGAAGGCTACCTTATTGGT |
| >CRISPR_IA_OS-B-VT-1 |
| CTCACTTCTCTTTGCTCATTACTCGCTTCTCTCTA |
| >CRISPR_IA_OS-B-VT-2 |
| AATACGAGCGAATTGACTGCGATATTCCTCACTACTATG |
| >CRISPR_IA_OS-B-VT-3 |
| AAGCCTTGACGAGCAAGGACTGCTCCTACCGTTCTAAGCACAA |
| >CRISPR_IA_OS-B-VT-4 |
| TTGGTTAAGGATGGCCTGGTCATAGACTTCTATGACT |
| >CRISPR_IA_OS-B-VT-5 |
| AACGAGACTAATTTTGGATCCGAATCGGAGTCTTTC |
| >CRISPR_IA_OS-B-VT-6 |
| ACGACATTCCCGAAGGCGCAACGATCAAATGGGG |
| >CRISPR_IA_OS-B-VT-7 |
| CATCCGGGAAACCGGGTGGGAGAGACTCTATTCAGC |
| >CRISPR_IA_OS-B-VT-8 |
| AACATCGTCGATGGTTTTCCCCATTTCGACTGGGC |
| >CRISPR_IB_OS-B-VT-1 |
| GAGCCATACTTCGACGGTGAAAAGGTGTACTGTAACGG |
| >CRISPR_IB_OS-B-VT-2 |
| AGGAAGTTTAACCGTAAGGTTGCTAAATCCGCAACTGTG |
| >CRISPR_IB_OS-B-VT-3 |
| TCATTACCTTAACGTGGAGCGGGGTTTAGGTAGCCGTGGCGATA |
| >CRISPR_IB_OS-B-VT-4 |
| TTAGCGTGCTACCCGCCACTGCCCACGCTGGTGGGTATTTTAT |
| >CRISPR_IB_OS-B-VT-5 |
| GTACGCCCGAGAGGGGTGCAAAAAGGGCGGTATGAGTA |
| >CRISPR_IB_OS-B-VT-6 |
| GTGTTCACCTGTCGAAGCCGTTCGGTCTGCGGGAAGTCTCCC |
| >CRISPR_IB_OS-B-VT-7 |
| GACAGCAATATTCTGTACCAACAAATGGAAAGAACAT |
| >CRISPR_IB_OS-B-VT-8 |
| TGAACTAGTCTTATATAAGGAGTGCCTACCTCAGGC |
| >CRISPR_IB_OS-B-VT-9 |
| GCCTACCTCAGGCACTCCTTTATTTTTTAGAGCAGGT |
| >CRISPR_IB_OS-B-VT-10 |
| AGCGAAGGAGCGGATCCGCAAGGCGAAAGCCCGCGGATTCCGC |
| >CRISPR_IB_OS-B-VT-11 |
| CTCGGTGTACCAGGTTACTGTCTTTCGGCACCTAGAAACCGGAGCC |
| >CRISPR_IB_OS-B-VT-12 |
| ATGGTGTTGACGGTAGCGGTGGTCGCTGGTACGATAGG |
| >CRISPR_IB_OS-B-VT-13 |
| GTCCGGTCGGCGGCATTGGGGATCCACGCGACGTCGCGTCT |
| >CRISPR_IB_OS-B-VT-14 |
| GGGTGGAGGTAAGTTTCTGTAGAGTTTACCGTTTACT |
| >CRISPR_IB_OS-B-VT-15 |
| CCGGTTCGCCGCCAAGCAACCTCACTGTGGAGCA |
| >CRISPR_I_metagenome_CYNA505TF-VT-1 |
| AGTAGAGACTTAGTATATTCGGATCTTTAAAAGC |
| >CRISPR_I_metagenome_CYNA505TF-VT-2 |
| TTATACTCTTATCTAGCTTCTTAAGCTCAGCAACAC |
| >CRISPR_I_metagenome_CYNA505TF-VT-3 |
| CCCTCTGCGTTAGCTCCCACCGGATGGCTGCTTCCAGATTCTGGAA |
| >CRISPR_I_metagenome_YMBDU19TF-VT-1 |
| TTTTCCTGGGTGTTGTTTTATCCCTGTAAAGGGAGGT |
| >CRISPR_I_metagenome_YMBDU19TF-VT-2 |
| CGAGCTAGCAATTCCCGAACCTTATCGGCGTTAATGGGAA |
| >CRISPR_I_metagenome_YMBDU19TF-VT-3 |
| CAGTGGGCTTCACCCCAAATTCTTTGAGCTGCCGCCGCAACGCAGC |
| >CRISPR_I_metagenome_YMBDU19TF-VT-4 |
| TCTCTCAACTCGTGTTCTACTGTCAGTAACTCAAGTAAT |
| >CRISPR_I_metagenome_YMBDU19TF-VT-5 |
| ACCCAATCTGGGTGGGCCTCGATGAAGTGGTCAGAA |
| >CRISPR_I_metagenome_YMBDU19TF-VT-6 |
| GACGCCATCCAAATGGACATGGCGTCGGATTTATCGGCA |
| >CRISPR_I_metagenome_YMBCA60TF-VT-1 |
| TACTCTACTTCTTTATTGATAATGATTGATATAAA |
| >CRISPR_I_metagenome_YMBCA60TF-VT-2 |
| TAGGGGAAGCCTCCACTCGATACAAGTATAAGTACCC |
| >CRISPR_I_metagenome_YMBCA60TF-VT-3 |
| AGAACTGCCCAAGAATACTCGTCGTGGTGGTCGGACTT |
| >CRISPR_I_metagenome_YMBCA60TF-VT-4 |
| TTGCAAACGAGCTTCTGCCCAAGTAATAGGACCTAGAG |
| >CRISPR_I_metagenome_YMBCA60TF-VT-5 |
| TTTGAAGGGGAATTAATAGCAAAAGGTAGAGAGG |
| >CRISPR_I_metagenome_YMBCA60TF-VT-6 |
| TAAACAGAGAAAGGGCTTAGATTAATTACTAAGCCCTTTCT |
| >CRISPR_I_metagenome_YMBCA60TF-VT-7 |
| TTCGCGGTAGCTACCCCGGTCGAGCCAGTTACTTACGAGTA |
| >CRISPR_I_metagenome_YMBCA60TF-VT-8 |
| ATCGAATACATTTTGGAGGCTCTAAGAAGGAGGAAGT |
| >CRISPR_I_metagenome_YMBCA60TF-VT-9 |
| GCCGAGATATCAAGAAGGTCAGCGATAGAGCCAAACGGCTTCAAGAG |
| >CRISPR_I_metagenome_CYPAN67TR-VT-1 |
| TCGAGCTTGATGGTGGGATCATCAAGCCGAAGGAGGTCAACGCGAA |
| >CRISPR_I_metagenome_CYPAN67TR-VT-2 |
| AGTACATGTTTTCGACAATTGCCTCGGTGCTCTTTGGAAA |
| >CRISPR_I_metagenome_CYPAN67TR-VT-3 |
| CTGGATGTTCAACGAGCGAGTGGAAAGATACTATGTGG |
| >CRISPR_I_metagenome_CYPAN67TR-VT-4 |
| TTCTCGATGTGGCTAGCAGGGTCGGTGCTTTTCCAAAG |
| >CRISPR_I_metagenome_CYPAN67TR-VT-5 |
| TATGTCTTCAACAACCGCGAGGACTATTTCGCAAACTGCA |
| >CRISPR_I_metagenome_CYPAN67TR-VT-6 |
| TTGAAGAAGTACCCACTATATTAATTGAGTCTGAC |
| >CRISPR_I_metagenome_CYPAN67TR-VT-7 |
| AAGGGAGGATTAGTATGGTTATGAAGTTCTGGG |
| >CRISPR_I_metagenome_CYPEB92TR-VT-1 |
| AGTTCGTCGTCACGTAGGGAGCACTCGACTAAGTACTCCC |
| >CRISPR_I_metagenome_CYPEB92TR-VT-2 |
| ATACAAGGGAGGTTGCTATGGAACCGGTGATCACTATCACTC |
| >CRISPR_I_metagenome_CYPEB92TR-VT-3 |
| GATTTTTAGGATAAGTTTCTGATAGTAGAATCCTAAATAGCTGC |
| >CRISPR_I_metagenome_CYPEB92TR-VT-4 |
| GGTGACCAAAGGCCTTCCCATCCGGTTTCACGGATG |
| >CRISPR_I_metagenome_CYPEB92TR-VT-5 |
| GTAGCCATAGGAACCTCCTAGGTTCAATTTGGTTATTACAG |
| >CRISPR_I_metagenome_CYPEB92TR-VT-6 |
| TTTGGGTGTTACAGGAAACCGGATGGCGTAGACTATTCAA |
| >CRISPR_I_metagenome_CYPEB92TR-VT-7 |
| GACACACCGCCAATCGGTGAGGTTTACTAAAAATGGATGTTGATCCCTACCTTCTAAAAATGGAT |
| >CRISPR_I_metagenome_CYPEB92TR-VT-8 |
| GTCACATGTACAAGTACACTAAGGAGCAAATCTTGAGGTCTTCT |
| >CRISPR_I_metagenome_CYPEB92TR-VT-9 |
| GGAGAATTTCCTCTTTCGTGAACATAACCGACCTC |
| >CRISPR_I_metagenome_CYPA321TF-VT-1 |
| TCAACAACTCCCACACTTCTTCTTGAGATATGTTGGAGC |
| >CRISPR_I_metagenome_CYPA321TF-VT-2 |
| AGGATAAAGTAGAAGTTGGAAGTGATCGAGTTCATTT |
| >CRISPR_I_metagenome_CYPA321TF-VT-3 |
| AGGATAAAGTAGAAGTTGGAAGTGATCGAGTTCATTT |
| >CRISPR_I_metagenome_CYPA321TF-VT-4 |
| AACCCAGCTACAGTGATGTTCCAACGCTCTTGCATAGCATCC |
| >CRISPR_I_metagenome_CYPA321TF-VT-5 |
| CGCTTCACGTTTGGACTCCTTTATTGAAAATGTGCCTAA |
| >CRISPR_I_metagenome_CYPA321TF-VT-6 |
| GAATGCCAACACGCCAGTGAGGCTATCGACGGTCCAACCGT |
| >CRISPR_I_metagenome_CYPA321TF-VT-7 |
| AGCCCTAAGGCTTCAGCCACGGACAAGCGAATGTTCTTGAC |
| >CRISPR_I_metagenome_CYPA321TF-VT-8 |
| AGTTCGACGGGATCCCACTCTTCGAGGTCGGTGAGCCAATCTA |
| >CRISPR_I_metagenome_CYPA321TF-VT-9 |
| ATGAGTCTTTTCCTGAAGTTCTCATCACGCAAT |
| >CRISPR_I_metagenome_CYPA321TF-VT-10 |
| CTCGATTGCGAACGGAGGTTCTTCTGGACTTTTTTGT |
| >CRISPR_I_metagenome_CYPAN59TF-VT-1 |
| GATCTGTTGCTTGGTGTACTTCATAACAGACTCCTTC |
| >CRISPR_I_metagenome_CYPAN59TF-VT-2 |
| GGCAATCGGTGATCGCTGGTAGTAATCCGAAGGAATC |
| >CRISPR_I_metagenome_CYPAN59TF-VT-3 |
| AGCAGGTCCGCACGATCCAGCATCTGTTTGGGCAAA |
| >CRISPR_I_metagenome_CYPAN59TF-VT-4 |
| TCCTGCAGGATAACCCAACGCTCCCGAAAGTAGAAAACTT |
| >CRISPR_I_metagenome_CYPAN59TF-VT-5 |
| GGCATCACAGTCGTGAAGGCCTGCTGGAAGACAGCACCTTGACT |
| >CRISPR_I_metagenome_CYPAN59TF-VT-6 |
| CCCTCAGCTTGGCGCTTCGCGCATTCAATGTAGCGGGT |
| >CRISPR_I_metagenome_CYPAN59TF-VT-7 |
| GGGGCGAGATACGCCTTCCGGTCCTTCACTGACCATACGG |
| >CRISPR_I_metagenome_CYPBI49TR-VT-1 |
| CTCGTTAAGAGGCTTGTCAGCCTCCCTGCTAATAGCTCGGACT |
| >CRISPR_I_metagenome_CYPBI49TR-VT-2 |
| GGGAGTAGGGCTAGCTCTAGGTCTTACAGATAAGCGA |
| >CRISPR_I_metagenome_CYPBI49TR-VT-3 |
| GACAAGCCCCCCTCTCGGGCGGGCATTTTTGACTCCCCT |
| >CRISPR_I_metagenome_CYPBI49TR-VT-4 |
| GGTGGAAAGAGTTACCTGGATCCGCGAAGGCGAAAG |
| >CRISPR_I_metagenome_CYPBI49TR-VT-5 |
| CGTTGTATGCATCCTCCGCATTACGCGCTTTTGCTTTGGC |
| >CRISPR_I_metagenome_CYPBI49TR-VT-6 |
| CTGGAGCGGGAGCTGGCCAGGGTACAGGCCCAACAGG |
| >CRISPR_I_metagenome_CYPBW78TR-VT-1 |
| TATCCAACGCCGCTTTTTCATTGTCGAACTCCTTAGAG |
| >CRISPR_I_metagenome_CYPBW78TR-VT-2 |
| ATGAGCAGGACGCACCCGTCGATGCCGAGGTTATCGGC |
| >CRISPR_I_metagenome_CYPBW78TR-VT-3 |
| GAGTGAAACCCTTCCCGAGCGGCCAGCCATCTCAACCTCCCTAAG |
| >CRISPR_I_metagenome_CYPBW78TR-VT-4 |
| CTCTGCATCGATTTTCTCGTTAGTTATAGATCTTAAGA |
| >CRISPR_I_metagenome_CYPBW78TR-VT-5 |
| GAGGTAGTTAACCTCGTTGGTTATGATCGGTACACCAA |
| >CRISPR_I_metagenome_CYPBW78TR-VT-6 |
| ACTGGGTCCCCATCGAAGTCCCCGCCCATTGTCTCCCAGTCAG |
| >CRISPR_I_metagenome_CYPBW78TR-VT-7 |
| TTGATGACCCCACCATCAAGCTCGAAGTAGAAGCTGTCGG |
| >CRISPR_I_metagenome_CYPBW78TR-VT-8 |
| GGTAACTGCCCCTCAAAGATTGCATTCAGCACAGCCATAGCT |
| >CRISPR_I_metagenome_CYPBW78TR-VT-9 |
| ACATTTGTACCTCCCTTCTAGAGGGATAAAGGACACCCA |
| >CRISPR_I_metagenome_CYPBW78TR-VT-10 |
| ACATTTGTACCTCCCTTCTAGAGGGATAAAGGACACCCA |
| >CRISPR_I_metagenome_CYPD001TR-VT-1 |
| CCAAGAATCCTCCCAGCAGCTACACGAGTGTAGATCAATG |
| >CRISPR_I_metagenome_CYPD001TR-VT-2 |
| TGAAGACAACGCAAAAGGAGGTACTGTAGGAGAAATTGGGTGG |
| >CRISPR_I_metagenome_CYPD001TR-VT-3 |
| TTGCCGGGGAAGATGTAACCGAAGGCCATCAGGGCCATAGGCCC |
| >CRISPR_I_metagenome_CYPD001TR-VT-4 |
| GGACTTGGCATCACAGTCGTAAAGGCCTGCTTAAACACAG |
| >CRISPR_I_metagenome_CYPD001TR-VT-5 |
| TATGGGATATAGTCGTCAAACCGCTCGTACTGGTTAAA |
| >CRISPR_I_metagenome_CYPD001TR-VT-6 |
| GTCTTTAGACAAACGTAAGCAAGTTTTAGTCACTGGC |
| >CRISPR_I_metagenome_CYPD001TR-VT-7 |
| CGCTGGGACTTCCACGGAACAGCCGTGAACCCGGAGC |
| >CRISPR_I_metagenome_CYPD001TR-VT-8 |
| ACATACTCGATTACTTCGCTACCGTTTCCTTCCTCTATA |
| >CRISPR_I_metagenome_CYPD971TF-VT-1 |
| TTGACTTAAATTTGCCTTTAAAGGGCCTGGAGAGTA |
| >CRISPR_I_metagenome_CYPD971TF-VT-2 |
| CCAAATGCATGCTGTGGTATAAACAACCACACTACAGTGGAGT |
| >CRISPR_I_metagenome_CYPD971TF-VT-3 |
| GACGCCTCGGACAAGATTTGAACTTGTGACCGACCGCTT |
| >CRISPR_I_metagenome_CYPD971TF-VT-4 |
| TAGGAGGTTACAATGTCTGCTATCTTCTACGGTGTTCCAGG |
| >CRISPR_I_metagenome_CYPD971TF-VT-5 |
| GCTTTTCGTTAAGGGATATTTCTCCGAATAGCTGAAA |
| >CRISPR_I_metagenome_CYPD971TF-VT-6 |
| GCTCGGGAAGGGCCCATTCAACCAACATCTTTCCAGCTGC |
| >CRISPR_I_metagenome_CYPD971TF-VT-7 |
| CGCCTGGTGTACACTAGACGAGTCCAACATTGTCGATG |
| >CRISPR_I_metagenome_CYPD971TF-VT-8 |
| GACTGAATAACCTCCCCCCAACCAGCACCCAGGATTT |
| >CRISPR_I_metagenome_CYPD971TF-VT-9 |
| TTTGGCAGGGAATACAGGAACCTGCCTTCTAAAAAT |
| >CRISPR_I_metagenome_CYPD971TF-VT-10 |
| CGCCTGGTGTACACTAGACGAGTCCAACATTGTCGATG |
| >CRISPR_I_metagenome_CYPDX40TR-VT-1 |
| AAGGGCAAGTGCTTGCGTACAGCCGATGCTACATCCCCGGC |
| >CRISPR_I_metagenome_CYPDX40TR-VT-2 |
| TTTAGCCGAGCATCACGCTCGTTTTCGTAGCGGTACTTTGT |
| >CRISPR_I_metagenome_CYPDX40TR-VT-3 |
| TGCAACCTTTAATCGGAACTCCACTATAGTGGAGTTGCC |
| >CRISPR_I_metagenome_CYPDX40TR-VT-4 |
| TTGGTATACCTCGCTTAGGGGCTTGTCGGATTCCCT |
| >CRISPR_I_metagenome_CYPDX40TR-VT-5 |
| CTTCTCCATACCGGGTGGGTAGAACCGTCCGGAAACACGATT |
| >CRISPR_I_metagenome_CYPDX40TR-VT-6 |
| CGGACTGCAGTATTGACATCCTCCGCACTACGCGCT |
| >CRISPR_I_metagenome_CYPDX40TR-VT-7 |
| AAACGTAAACATCCGCAACCTGATGGAAGTCGGAAGC |
| >CRISPR_I_metagenome_CYPDX40TR-VT-8 |
| GATGGCTGGGTGTTACTGCCAATTTGGGTGGCTTTGTAT |
| >CRISPR_I_metagenome_CYPDX40TR-VT-9 |
| GACTTCACCATATACCGTTTGGTATATGTCGATACCGGC |
| >CRISPR_I_metagenome_CYPDX40TR-VT-10 |
| GTTTCGTTGTAAACAAACGTGTAGCGGGGCATAAAC |
| >CRISPR_I_metagenome_CYPEK86TR-VT-1 |
| TTCGCAATTATACGGAGCGCTCGGTCACGCGGTGTACCT |
| >CRISPR_I_metagenome_CYPEK86TR-VT-2 |
| CGTCTAGAGGCTGCCTGAGCTAGAGTAAGGCTACTGT |
| >CRISPR_I_metagenome_CYPEK86TR-VT-3 |
| GTGCCCAGAATTTCTTCTGGGACCGGCCTTTCTGAA |
| >CRISPR_I_metagenome_CYPEK86TR-VT-4 |
| TCGCGAATGTACTCGCAACGCTCTGCCCTCAGGATC |
| >CRISPR_I_metagenome_CYPEK86TR-VT-5 |
| ACCAACATGTAGTCCGCAGCTAAAATGCGCAAGGTGTACCA |
| >CRISPR_I_metagenome_CYPEK86TR-VT-6 |
| CATCCTCCAGACTAATCCCCAACTCATTTGCAAGCTCAC |
| >CRISPR_I_metagenome_CYPEK86TR-VT-7 |
| TCGTGATCGTAACGGTACTTAACCACCTCGTACCGAGCT |
| >CRISPR_I_metagenome_CYPEK86TR-VT-8 |
| TCACTCAGGATCTGTGCCAATGGGCGACGAAAATTCAGCCGA |
| >CRISPR_I_metagenome_CYPEK86TR-VT-9 |
| TGCCAATACCAAACAATCCGCTTGTTTGGTTCGTATGCATATGG |
| >CRISPR_I_metagenome_CYPEK86TR-VT-10 |
| GGGCTCCAGTCATCGTGGAGGACAATTTCCATGACAA |
| >CRISPR_I_metagenome_CYPFB72TF-VT-1 |
| TTTCTAACTCCGTCTAAACCTCCCCCAACCAACAGCAC |
| >CRISPR_I_metagenome_CYPFB72TF-VT-2 |
| TTCAAATACAAGCGCTTGTCACCATAAAGGTACTTTATT |
| >CRISPR_I_metagenome_CYPFB72TF-VT-3 |
| TTCGTCAGAGCTCTTTGGATCAAAATGCCACTTCCCTGC |
| >CRISPR_I_metagenome_CYPFB72TF-VT-4 |
| GCTGAGGTTCTTCGAAAGAACCAGGGCAACAGTCCGGTAGG |
| >CRISPR_I_metagenome_CYPFB72TF-VT-5 |
| GAGGTAGCCAAGTGGCTTTCAAGCGCAGAGCACCTAAC |
| >CRISPR_I_metagenome_CYPFB72TF-VT-6 |
| GTTATTCCCTGAATAAGGGAGGAGTCTATATGAACACC |
| >CRISPR_I_metagenome_CYPFB72TF-VT-7 |
| CTAAACCTCCTCCCCCAACCAACACACCCAGGTATTTC |
| >CRISPR_I_metagenome_CYPFB72TF-VT-8 |
| CACTCCGCCTACCCAGGATGGAGCGTCCACCGAGTAGGAGAA |
| >CRISPR_I_metagenome_CYPFB72TF-VT-9 |
| CCCATGATGGGGGGTATTTGAAGGTGCAGTCGCTCAAATAT |
| >CRISPR_I_metagenome_CYPGI71TR-VT-1 |
| GCAGTTTAGTTGTTCCTGCCTGAAGTTAGGAGGTTGAGAT |
| >CRISPR_I_metagenome_CYPGI71TR-VT-2 |
| AATCAATTGGCACAACAGGGCCGCAACTGTATTGCGGA |
| >CRISPR_I_metagenome_CYPGI71TR-VT-3 |
| ATCGCTCTGAAATTGGCCTAGAACCACTGGAACTGTACACA |
| >CRISPR_I_metagenome_CYPGI71TR-VT-4 |
| AGACGAAGAATTCGTCGACTACATCTTAAACAGTCTGAGAA |
| >CRISPR_I_metagenome_CYPGI71TR-VT-5 |
| ACATTTTCCCCGGCAAAGACCTGCAGCCGGGTGAGGT |
| >CRISPR_I_metagenome_CYPGI71TR-VT-6 |
| ATCGGACAGCCTGACATAGGAGCGATGCCAATACGGCGTAC |
| >CRISPR_I_metagenome_CYPH825TF-VT-1 |
| TTCGCTGCTTTGATCTGCTGACGCTGGACTTTTGTGA |
| >CRISPR_I_metagenome_CYPH825TF-VT-2 |
| TGCGGGGGAACCGGTAGTTCCCCACCCCGGAAAACCAG |
| >CRISPR_I_metagenome_CYPH825TF-VT-3 |
| CAGGTAATCACCCAGCGCCCTTTCCCCCATGTGATCAC |
| >CRISPR_I_metagenome_CYPH825TF-VT-4 |
| TGTGGAATTAGGTCAACACAGATTGTATTGACAGCTA |
| >CRISPR_I_metagenome_CYPH825TF-VT-5 |
| CTCCTGGAATAGCTTCTTACCTGCTCCCCTGCTGAA |
| >CRISPR_I_metagenome_CYPH825TF-VT-6 |
| TCTCGACATGGACTTTTTGTTTACTGAGGAACTTACGGA |
| >CRISPR_I_metagenome_CYPH825TF-VT-7 |
| AGAGCCTTAAGGCAATCACTCGCCTGCCTTAGGCTGC |
| >CRISPR_I_metagenome_CYPH825TF-VT-8 |
| TTCAGGTTTGGGTAGAAGAACGGGAGCCGGTCTTTCGA |
| >CRISPR_I_metagenome_CYPH825TF-VT-9 |
| TCCCTCCGGGATTTGAATACCGGTCCAAGTCGAATC |
| >CRISPR_I_metagenome_CYPH825TF-VT-10 |
| TCGAGCAGCTCATCGGGGGTCCAAACAAAGCGACGCTTGG |
| >CRISPR_I_metagenome_CYPH825TF-VT-11 |
| GCGGCCTTCTTGACCTTGCGGTTAAACCGCTGCCGCCGA |
| >CRISPR_I_metagenome_CYPHE06TR-VT-1 |
| GCCCATTGTGAACGAGCTAGAGCTGTCGACTTCCCGGA |
| >CRISPR_I_metagenome_CYPHE06TR-VT-2 |
| CTCGGGGGTTCGAATATCCAACATGTGCGGAGGCACTCCG |
| >CRISPR_I_metagenome_CYPHE06TR-VT-3 |
| CCGGGCTTCACAACTTGTGTCAATTGATTTAAGTCC |
| >CRISPR_I_metagenome_CYPHE06TR-VT-4 |
| CCCCCACTTCAAGGTGGGCCAGGATCGGGCGCTTATTAC |
| >CRISPR_I_metagenome_CYPHE06TR-VT-5 |
| GACGCTCCAAGTCCTCTGCAGAGAATTCCTAATTCGATCGG |
| >CRISPR_I_metagenome_CYPHE06TR-VT-6 |
| ACATAGCTGTGTCTCCCTTACACGGCCTACAAAACCA |
| >CRISPR_I_metagenome_CYPHE06TR-VT-7 |
| CACTTTGGCAAGTATGAGTGTAATATCACGGACATACTT |
| >CRISPR_I_metagenome_CYPHE06TR-VT-8 |
| ACAACCCACTGCCCCTTATATGGCTTAACCACAACAT |
| >CRISPR_I_metagenome_CYPHE06TR-VT-9 |
| AGGATAAAGTAGAAGTTGGAAGTGATCGAGTTCATTT |
| >CRISPR_I_metagenome_CYPHE06TR-VT-10 |
| TTCCTTCAGAGCTCGTAAGACCTCGCTGGCCTCGTATA |
| >CRISPR_I_metagenome_CYPHQ76TR-VT-1 |
| ATCGAAGCCTGTCGGTTCGTAAGAAACCGGCTGGTTACC |
| >CRISPR_I_metagenome_CYPHQ76TR-VT-2 |
| CTCGGGCAGCTGCCTTGACCCCTAAGTCGGGTCGAAAAAT |
| >CRISPR_I_metagenome_CYPHQ76TR-VT-3 |
| CTCAGGAAGAGGGAGGTGAAAGGTCAGTAAGAAGTATC |
| >CRISPR_I_metagenome_CYPHQ76TR-VT-4 |
| GCAAATTTAAGCCAAGTATAGACTTAGCTCGAAAAAA |
| >CRISPR_I_metagenome_CYPHQ76TR-VT-5 |
| AGATCTACCATGATTACACTAATTGCACCCTCCACAACC |
| >CRISPR_I_metagenome_CYPHQ76TR-VT-6 |
| CACTTATGGGTGGCACTGCAGTTCTGCAGCCGGATGGAACA |
| >CRISPR_I_metagenome_CYPJ722TR-VT-1 |
| CCCGCATCGACTTAGTAATGGGACGTTCAAAGGC |
| >CRISPR_I_metagenome_CYPJ722TR-VT-2 |
| GACTTCCACGACGTGGAACGTATCTTCCCCGATGTAGA |
| >CRISPR_I_metagenome_CYPJQ75TR-VT-1 |
| TACAGTACAGGTCAATGTTGAGGGTACATATAGAGA |
| >CRISPR_I_metagenome_CYPJQ75TR-VT-2 |
| ATTTCCTCGATCGGTTTGGGGCTGTTGGCCTTGACGTTAA |
| >CRISPR_I_metagenome_CYPJQ75TR-VT-3 |
| GTGTATATGGACAGCACTTGGCTATCAGTCGGAGGTT |
| >CRISPR_I_metagenome_CYPLK34TR-VT-1 |
| CTATAGCCTGGTTACAGGCTGAAAAGCGGCGCCAGCGGTTTCCG |
| >CRISPR_I_metagenome_CYPLK34TR-VT-2 |
| TGCGGTATTCCTCCTTTGCCTCATGTGGCCAGAATTGGGCCAC |
| >CRISPR_I_metagenome_CYPLK34TR-VT-3 |
| CCCAGTACGTCAATGGCACCCTGCGTTCCGAAAGGA |
| >CRISPR_I_metagenome_CYPLK34TR-VT-4 |
| TGACTGATTAGCCGTACCCCTGCAGCCGCAAACATGGCGGG |
| >CRISPR_I_metagenome_CYPLK34TR-VT-5 |
| TTTATTACAGTGCCGGTCTGCACTGACCCCGGTATAGT |
| >CRISPR_I_metagenome_CYPLK34TR-VT-6 |
| AATTCATTTGGATTTTGGTAATCATGTTAACCTCCA |
| >CRISPR_I_metagenome_CYPLK34TR-VT-7 |
| TTTCATCTGGCCGGCTATTATTTTAGCGGCCCGATCTTTG |
| >CRISPR_I_metagenome_CYPLK34TR-VT-8 |
| GCTTCGCGAAGTGAGTTCGTAACCAAACGAGTCCGGATG |
| >CRISPR_I_metagenome_CYPLK34TR-VT-9 |
| GATAATCTTCTCTGCGCGAGCCCTCGGTGTGGCTCGCTGG |
| >CRISPR_I_metagenome_CYPLK34TR-VT-10 |
| AGGACTAGCTTTACTTTTGTCAACGGTTCGAGGGGTAACTCT |
| >CRISPR_I_metagenome_CYPLU89TR-VT-1 |
| GGCCTTGGCTTTAATCACCTCTTCGATTTGCTCGATCC |
| >CRISPR_I_metagenome_CYPLU89TR-VT-2 |
| CATTGGGAAGAAACAACACCCAGGTATGAACCTGGGT |
| >CRISPR_I_metagenome_CYPLU89TR-VT-3 |
| AAACAACACCCAGGTATGAACCTGGGTGCTGTTGGTTGG |
| >CRISPR_I_metagenome_CYPLU89TR-VT-4 |
| CCTGGGTGTTGTTTCTCCTAAACAAAGGGAGGTTAAT |
| >CRISPR_I_metagenome_CYPLU89TR-VT-5 |
| TCCCAACACGGCTAGTAAAGTCGTCAATGAAGAATTGA |
| >CRISPR_I_metagenome_CYPLU89TR-VT-6 |
| TAGACCTACTACAATTGCACAAAGTCAAATGAAGA |
| >CRISPR_I_metagenome_CYPLU89TR-VT-7 |
| TTTGGCTAGCGCAATTTGCTTGTCATTGAGTAAAGTCTTACT |
| >CRISPR_I_metagenome_CYPLU89TR-VT-8 |
| GTGGCAGCCTTTGTTTAGGTTAGTTAACAAATTGATTGGA |
| >CRISPR_I_metagenome_CYPLU89TR-VT-9 |
| GGTGACTTCGAAAAACTCGCAATACGAAAGGTACTCTT |
| >CRISPR_I_metagenome_YMAA880TR-VT-1 |
| AGGGTTAACTGAAGTCACGATCCTACCTGAAGTGGTAGC |
| >CRISPR_I_metagenome_YMAA880TR-VT-2 |
| TTCGTGTTATCTATTCTAGCAAGCGCCGTTTTGTGCGCG |
| >CRISPR_I_metagenome_YMAA880TR-VT-3 |
| AAAAAGCCGAGCGCAAGCGGCAGAAGTTTACTAAGAAAG |
| >CRISPR_I_metagenome_YMAA880TR-VT-4 |
| CACACGATGCGAAAACGGAGTACCTACGCCGCTGGAC |
| >CRISPR_I_metagenome_YMAA880TR-VT-5 |
| GACCTACTACCGACTCTGCTGTGCCATCCAGAGGCTGTACG |
| >CRISPR_I_metagenome_YMAA880TR-VT-6 |
| GCGGCGATAGAAAAACTAGACTCTCATGACAGCCCCGTA |
| >CRISPR_I_metagenome_YMAA880TR-VT-7 |
| AGAGAACATGTATTCATTGGCTCTATTACGTCTGATCAC |
| >CRISPR_I_metagenome_YMAA880TR-VT-8 |
| AGCTCCAGGCTCATTAGCCTAATTGGCCCAAATTCGGG |
| >CRISPR_I_metagenome_YMAA880TR-VT-9 |
| CATGAAGCGGAGGTGGAAATTGGCGGCCAGTGTTTTACT |
| >CRISPR_I_metagenome_YMBBA67TR-VT-1 |
| GAGGTGTGGACCAGTGATGGTGTTGGTGGGCGTAAATTGACCTT |
| >CRISPR_I_metagenome_YMBBA67TR-VT-2 |
| CTGCGGGATACGATGCGCCAGTCCATGTTTAAGAGGAGC |
| >CRISPR_I_metagenome_YMBBA67TR-VT-3 |
| GGCGGAGTCCCAGCATCGGACGGTTCCAAGTTGTTGGG |
| >CRISPR_I_metagenome_YMBBA67TR-VT-4 |
| CCTGTAAAGGGAGGTCATCATGAACGTCATGAAGTTCGCCGG |
| >CRISPR_I_metagenome_YMJA680TF-VT-1 |
| GAAAATAAAGCTTCTTCTCAGATTAACTGGGATGAAGT |
| >CRISPR_I_metagenome_YMJA680TF-VT-2 |
| AGTTTACCTTTAAGGACCCAGATGAGTACTTGTTGTG |
| >CRISPR_I_metagenome_YMJA680TF-VT-3 |
| GTTTATGCCCCGCTACACGTTTGTTTACAACGAAAC |
| >CRISPR_I_metagenome_YMJA680TF-VT-4 |
| GCCGGTATCGACATATACCAAACGGTATATGGTGAAGTC |
| >CRISPR_I_metagenome_YMJA680TF-VT-5 |
| ATACAAAGCCACCCAAATTGGCAGTAACACCCAGCCATC |
| >CRISPR_I_metagenome_YMJA680TF-VT-6 |
| GGTTCTGGCAAACCTGGAACAACGGGTTGCCGATTAAGGGCTC |
| >CRISPR_I_metagenome_YMJA680TF-VT-7 |
| GCTTTACGAGCTCATGGACAAATACCAGTGGGATGTCTCC |
| >CRISPR_I_metagenome_YMJA680TF-VT-8 |
| CGGTGGGATTACCCACACACCCCTGTACTACAGCCCTGACAA |
| >CRISPR_I_metagenome_YMJA680TF-VT-9 |
| GCTCCACTCTGGACGGCTGGGTAGGATCCTGGAGTAGTGAGG |
| >CRISPR_I_metagenome_YMJA680TF-VT-10 |
| CTGCGCTTAATCACAGACGGTCCCATATCTCTAATGGG |
| >CRISPR_I_metagenome_YMJAH44TF-VT-1 |
| TTCAGTGTCCGGAGCATTTCGTCGATTTGCTCCTGTGTGAA |
| >CRISPR_I_metagenome_YMJAH44TF-VT-2 |
| ATCCTCAATTTGCTTGGTCCAACGCTTCCGGAATTCCT |
| >CRISPR_I_metagenome_YMJAH44TF-VT-3 |
| CTGTGAAGCCACACAGGCTTGCACAGCAGCCCAGCCT |
| >CRISPR_I_metagenome_YMJAH44TF-VT-4 |
| AGCTCCGACTACCTCCATGCAGTAAGCAGACTCAACC |
| >CRISPR_I_metagenome_YMJAH44TF-VT-5 |
| TACCTCATGGTCATGTTTGTACTTAGCCAGCTCGTAGCTGGTGA |
| >CRISPR_I_metagenome_YMJAH44TF-VT-6 |
| CCCATTGGGTCGTACAGTCTGCAGCACACTGGGGTACTGT |
| >CRISPR_I_metagenome_YMAAA02TF-VT-1 |
| ACAACACCCAGGTGAAATACCTGGGTGTTGGTGTGTTGGTGGG |
| >CRISPR_I_metagenome_YMAAA02TF-VT-2 |
| GTGGTGAAGGAAATCGGGGGTCGCAACTACTACTGGAA |
| >CRISPR_I_metagenome_YMAAA02TF-VT-3 |
| 3GACAATAACTTTGGATTGACGAATTATCAGTTAGCA |
| >CRISPR_I_metagenome_YMAAA02TF-VT-4 |
| ATCAACATTATCTAGTGTCCAAATTTGTGGCTACCAC |
| >CRISPR_I_metagenome_YMAAA02TF-VT-5 |
| ATGGCCGCTATTTGATTGCGGCTCCCTACCGCAATG |
| >CRISPR_I_metagenome_YMAAA02TF-VT-6 |
| TACTGGTCAAGTTGGACCGGGACAAACCAAACTAACCTGGGAA |
| >CRISPR_I_metagenome_YMACC44TR-VT-1 |
| TTCTTGACGGGTTGGGAACCGTGAAGCTGCCAGTA |
| >CRISPR_I_metagenome_YMACC44TR-VT-2 |
| AGCTCTGATGGTAACACGCGATCAAAGATCGCGATTAAA |
| >CRISPR_I_metagenome_YMACC44TR-VT-3 |
| CGCGTTCATGGTTTACCTCCCTTTACGGGATAGGAAGAAC |
| >CRISPR_I_metagenome_YMACC44TR-VT-4 |
| GTCCACACCTCCCCATTTGGGTCAGTGTATATAGC |
| >CRISPR_I_metagenome_YMACC44TR-VT-5 |
| TCTGTTAGCACGGGAGATCCTCGTGTATCCCATCGCCTC |
| >CRISPR_I_metagenome_YMACC44TR-VT-6 |
| GGGCCACTTGCGTGGTCTTCCGCTGGTCCTTGATGACCATG |
| >CRISPR_I_metagenome_YMACC44TR-VT-7 |
| GCTAGGGCTTCCGCTCTCCTCTTCGCCCGATTTGTAAG |
| >CRISPR_I_metagenome_YMACC44TR-VT-8 |
| TTAATTTCACTCAGTTTACGCGAGAAGGTGTAAACGTATCCA |
| >CRISPR_I_metagenome_CYOCM11TF-VT-1 |
| GTCGCTTTGTACGGGCACGGTTGGTTACCAACAGCCTCTT |
| >CRISPR_I_metagenome_CYOCM11TF-VT-2 |
| GTGAAGCGGGGGTGGATCTTGGTAGAAAAGGGCTGGAT |
| >CRISPR_I_metagenome_CYOCM11TF-VT-3 |
| TAGGCTCGGTATACACTCTGGGGTGTCCGCTCCTCTG |
| >CRISPR_I_metagenome_CYOCM11TF-VT-4 |
| CCCAGTCGTTATCCACCTGGGAGACTTCGGTACTACCCTG |
| >CRISPR_I_metagenome_CYOCM11TF-VT-5 |
| TCGAGCAACAGAATCAGCTCACCTCCCTTCCGCTTTAGGGAGATG |
| >CRISPR_I_metagenome_CYOCM11TF-VT-6 |
| TTCCTAGTTCTTGGTTTGGTGTAAGTGCTGTTGATGC |
| >CRISPR_I_metagenome_CYOCM11TF-VT-7 |
| TCCGGTATCTTGCTGGCGTACTCGGTGAGGTGAGTTGGAT |
| >CRISPR_I_metagenome_CYOCM11TF-VT-8 |
| ACTGACTAACCTCCCCCAACAAGGCACCCAGGATTTTCCTGGGT |
| >CRISPR_I_metagenome_CYOCM11TF-VT-9 |
| TCAAAGTGGGGGTAGCCCTCGAGCAGGTTACACTCGT |
| >CRISPR_I_metagenome_CYPBV46TF-VT-1 |
| CTCAATTCGGCTCGGGGAGCACTATGACCCGAACTACGA |
| >CRISPR_I_metagenome_CYPBV46TF-VT-2 |
| AGCGAATCAGGTACCTGTTAAATCGACCCGAAGCAGA |
| >CRISPR_I_metagenome_CYPBV46TF-VT-3 |
| CCGGGAAACCGGATGGCAAAGGCTATTCGGTCCGACCGAGGT |
| >CRISPR_I_metagenome_CYPBV46TF-VT-4 |
| CCACCCGAAACTTTTCTATCGACGACCTCGATGAGTCTTCTT |
| >CRISPR_I_metagenome_CYPBV46TF-VT-5 |
| GTGAACTAACCTCCCCCAGCAACACCCAGGATTTTTCCT |
| >CRISPR_I_metagenome_CYPBV46TF-VT-6 |
| AACAAATGGGAAACACTTCTTTATCTCGCTGCAGACC |
| >CRISPR_I_metagenome_CYPBV46TF-VT-7 |
| ACGAGGCGGCTATAGTAGAGCTAGATTACCACAGTCAG |
| >CRISPR_I_metagenome_CYPBV46TF-VT-8 |
| TAGCATGGGGTGTATTTGAGGATGAGTTATTCGGATACCCCTTCCA |
| >CRISPR_I_metagenome_CYPBV46TF-VT-9 |
| ACCTCTTTGTTGCTACAGACGCTACAACAAGAGTGGC |
| >CRISPR_I_metagenome_CYPBV46TF-VT-10 |
| AACTCTATCCTCTTGGTTGACAGTTAAAGTAATGAAAT |
| >CRISPR_I_metagenome_CYPC933TR-VT-1 |
| GATCCCGCTGCCACCCCACAGCGTTAGCGAGGGGATC |
| >CRISPR_I_metagenome_CYPC933TR-VT-2 |
| GCGAAGAGCATTCGCCTGTCCGTGGCTGAAGCCCTCGGGCTTCC |
| >CRISPR_I_metagenome_CYPC933TR-VT-3 |
| TAAATCACCCGCACACCTCGCGGTGTGGGTTCAATACGG |
| >CRISPR_I_metagenome_CYPC933TR-VT-4 |
| GTAAACGCGAAGAATATTCGCATGACGGTGTCTGAAGC |
| >CRISPR_I_metagenome_CYPC933TR-VT-5 |
| TTTTGCCTAAGGACATGCTAGCCCGTGCGGGGCTGCT |
| >CRISPR_I_metagenome_CYPC933TR-VT-6 |
| CTATCTGGAAACTCCCTTGTACGAGCTGAGTGGT |
| >CRISPR_I_metagenome_CYPC933TR-VT-7 |
| TCACCACATTCTACAAAGAGTGGAGCAAGTTGATGTTAAC |
| >CRISPR_I_metagenome_CYPC933TR-VT-8 |
| CACGACAGTTGCAGTGGACAAGGCTTTGGACACCTTGCG |
| >CRISPR_I_metagenome_CYPC933TR-VT-9 |
| AGCAAAAAGTTCTACGCCGGACCAAGTTTCCCGGAGCA |
| >CRISPR_I_metagenome_CYPC933TR-VT-10 |
| ATCCGCAAAGCTTTGCGGATGGTTCAGCTCATCTAAAC |
| >CRISPR_I_metagenome_CYPC933TR-VT-11 |
| GCGATCGTTGAAGAAGAATAACTGATTGTTGGGAGTAG |
| >CRISPR_I_metagenome_CYPCF80TF-VT-2 |
| CTCAGTAGGATAGCGCTCCCTTAGGCACAGCACACACGAATACGTGTGGTCTTTGTCTGAGAACAACA |
| >CRISPR_I_metagenome_CYPCF80TF-VT-3 |
| CTCAGTAGGATAGCGCTCCCTTAGGCACGTAGAAAA |
| >CRISPR_I_metagenome_CYPCF80TF-VT-4 |
| GGCCAATCTGGACTAGCAAGCCCGTAGCTAGCTCGAAAA |
| >CRISPR_I_metagenome_CYPCF80TF-VT-5 |
| CACACCTGGATCTGTGCGGGCTTTATCTTGGCCGACAGGCTG |
| >CRISPR_I_metagenome_CYPCF80TF-VT-6 |
| GGCACATGCCAGTCGTAGCGGTCCTCAATCATCCAGAAACC |
| >CRISPR_I_metagenome_CYPCF80TF-VT-7 |
| ATTGTGCAGTATGGATACTGCACCGGAGGGATAGGACCGTTA |
| >CRISPR_I_metagenome_CYPCF80TF-VT-8 |
| CCTTTGTAAATATTGAGCTCAGTCTGGAGCGAGACCCA |
| >CRISPR_I_metagenome_CYPCF80TF-VT-9 |
| TTGTCCAGGACTCCGACTTCCTCTAGGTACCACCCGTC |
| >CRISPR_I_metagenome_CYPEU56TF-VT-1 |
| TATACCGAGCCTATTAACAAAGAGGTAGAGCAGGTAT |
| >CRISPR_I_metagenome_CYPEU56TF-VT-2 |
| AATTCGCGGTAGCTACCCCGGTCGAGCCAGTTACTT |
| >CRISPR_I_metagenome_CYPEU56TF-VT-3 |
| CTTCTGCCAGGTACCCTTTGATGGGCCCTCCATCCGGAATC |
| >CRISPR_I_metagenome_CYPEU56TF-VT-4 |
| ACAGGGGGCACGGAAACCCACCGAGCAGCTCATTTTC |
| >CRISPR_I_metagenome_CYPEU56TF-VT-5 |
| CTCCAAATTCTGTAAGCACAGAAACGGGGGGTGGTACCT |
| >CRISPR_I_metagenome_CYPEU56TF-VT-6 |
| GCCAGTGGTAAAGTGCTGGCCGTTGATTGTTAGCTCTGCTTC |
| >CRISPR_I_metagenome_CYPF150TR-VT-1 |
| GTGGGCAGTCAAAGATCGGAAGATCTACCTCGCCCCCAA |
| >CRISPR_I_metagenome_CYPF150TR-VT-2 |
| GCTAAAGAGTTCGTTGAACGAACTCCCCAGGGTTTCCAGTT |
| >CRISPR_I_metagenome_CYPF150TR-VT-3 |
| GCCCTTCGCTCAGTTGATAAGGCTGCAGAAATTTGTAGTCGTAAC |
| >CRISPR_I_metagenome_CYPF150TR-VT-4 |
| CTGCTATTGCGGGGATCCGGGTTGCTTTCAGCGTGTAACA |
| >CRISPR_I_metagenome_CYPF150TR-VT-5 |
| GCTACATAAAGTACCCCCCATCGTGGGCAGTGGCGGGT |
| >CRISPR_I_metagenome_CYPF150TR-VT-6 |
| ATCTACGGGAGAATGGCTGACTGCCTACACCAATG |
| >CRISPR_I_metagenome_CYPF150TR-VT-7 |
| CAGTGGACATATAATTTTTAGCGATATATCCACTATTTCCA |
| >CRISPR_I_metagenome_CYPF247TF-VT-1 |
| TCACTTCTCTTTGCTCATTACTCGCTTCTCTCTA |
| >CRISPR_I_metagenome_CYPF247TF-VT-2 |
| ATCCCGCTGCCACCCCACAGCGTTAGCGAGGGGATC |
| >CRISPR_I_metagenome_CYPF247TF-VT-3 |
| CGCCGAAAAACTGGTGGAGAACTTTTTCTCCTCGGCCCT |
| >CRISPR_I_metagenome_CYPF247TF-VT-4 |
| CTCCTTTTTATTTTTTGAAAACTTACTCTCTTAGGAGGTA |
| >CRISPR_I_metagenome_CYPF247TF-VT-5 |
| CCTTCTCTCCTCACCAATGGAGTGGACCAGGCTCCAC |
| >CRISPR_I_metagenome_CYPF247TF-VT-6 |
| TCGAGGGTCAACGCAAGACCTTAAAGCTGACTGA |
| >CRISPR_I_metagenome_CYPGU34TR-VT-1 |
| CTCACTTCTCTTTGCTCATTACTCGCTTCTCTCTA |
| >CRISPR_I_metagenome_CYPGU34TR-VT-2 |
| GTGAGTTTCGCCTCAAGCCCACATGGCTTCCTCAACTCGACGG |
| >CRISPR_I_metagenome_CYPGU34TR-VT-3 |
| CTGTAATAAATCACGGGATGCGGTCCCGACTAATTC |
| >CRISPR_I_metagenome_CYPGU34TR-VT-4 |
| TCCGTGTGTCGTTTTACGTACCAGATCCCGACTGGT |
| >CRISPR_I_metagenome_CYPGU34TR-VT-5 |
| TGAGCGGGTAATCAGGGAGCGTATGCCCGGCTCTTACTG |
| >CRISPR_I_metagenome_CYPGU34TR-VT-6 |
| ACCGGTTAGGCACATTTTCAATAAGGAGTCCGAAATGCGTAAGCG |
| >CRISPR_I_metagenome_CYPHK58TF-VT-1 |
| GCCGCAGTCCGTCGACACCTGCCCCTATTCGGGGTCAGTG |
| >CRISPR_I_metagenome_CYPHK58TF-VT-2 |
| ATTGGTCTTTCAAAGCTCATATGTACCTCCTTAGGTAGGAA |
| >CRISPR_I_metagenome_CYPJW61TR-VT-1 |
| CCAGAGGACGGAGGGTTTGGGAAGTTTTGTCCTTACGC |
| >CRISPR_I_metagenome_CYPJW61TR-VT-2 |
| ATCTCGAGCGAGACAAGTCTCGGAAGTCCTAGAGCCTCC |
| >CRISPR_I_metagenome_CYPJW61TR-VT-3 |
| GTCCATGGCGGACAGGTTAGCTTGCATATTCAATTGTCC |
| >CRISPR_I_metagenome_CYPJW61TR-VT-4 |
| GAATTAGTCGGAGCCGCTTCCCGAGATTTATTACAG |
| >CRISPR_I_metagenome_CYPJW61TR-VT-5 |
| GCCCCGCACGAACCAGCATGTCTTTGGGTAGAATCACT |
| >CRISPR_I_metagenome_CYPJW61TR-VT-6 |
| ATTGTCGGGAGGATTTCTTTCGGTACCGGACGTTCACAG |
| >CRISPR_I_metagenome_CYPJW61TR-VT-7 |
| TCAGCTTCGCGCCGATTCAACAGGTACTTAGCCCGTTCTTGAGC |
| >CRISPR_I_metagenome_CYPLL02TR-VT-1 |
| GGTCGGAGAGCCAATCCATCATTGGCTCAAAACTAGAATT |
| >CRISPR_I_metagenome_CYPLL02TR-VT-2 |
| AAGAGACAATCTGCCTAGGCATACGGAAGTCCAAAGTG |
| >CRISPR_I_metagenome_CYPLL02TR-VT-3 |
| AAGATGCGAACGGCTTCCCAGTCGTCCATAGCAGACTCAA |
| >CRISPR_I_metagenome_CYPLL02TR-VT-4 |
| TCCCCAGGTGATCACCGCGCCTTCCGGAACATCTGCG |
| >CRISPR_I_metagenome_CYPLL02TR-VT-5 |
| CTTACTATGCGAGTTAGTCCTCATCGTCGGACCAAAAC |
| >CRISPR_I_metagenome_CYPLL02TR-VT-6 |
| AAGCCACCCAGGACATAGTAGTGGGGGTGGACCCCCTCGTAG |
| >CRISPR_I_metagenome_CYPLL02TR-VT-7 |
| TAGAGAGAAGCGAGTAATGAGCAAAGAGAAGTGAG |
| >CRISPR_I_metagenome_CYPM809TR-VT-1 |
| CAGACCTGGGGAGTATGATAGTACTTCCAACTATTCTCCAGG |
| >CRISPR_I_metagenome_CYPM809TR-VT-2 |
| ACTCACCTCCCCGAGTACCCCAGCAAGATACCGGATATACT |
| >CRISPR_I_metagenome_CYPM809TR-VT-3 |
| GGATTTAATAGAACGAAAAATCCGTAGAGGAATGCGTAAG |
| >CRISPR_I_metagenome_CYPM809TR-VT-4 |
| CTGTAGATTTGTAATGCCAATGCCCGCGCACGGCGTTTTGCG |
| >CRISPR_I_metagenome_CYPM809TR-VT-5 |
| TTCGGAGTCCAACCCCGGTAGAACGGGGTTGCGCCAAGCCTC |
| >CRISPR_I_metagenome_CYPM809TR-VT-6 |
| GAGATTGACTTGCCCTGGGTTGGGCCGGCCAGTTGTAAAGA |
| >CRISPR_I_metagenome_CYPM809TR-VT-7 |
| ACCACGAGGCGTGCGTTGAATACGGTTGCGGAGATGGGGGA |
| >CRISPR_I_metagenome_CYPM809TR-VT-8 |
| TTCGCCTGACCTGGGATGTACTGGTATTGGATCAC |
| >CRISPR_I_metagenome_YMBEK03TR-VT-1 |
| TTGATGGGATTCGGCTCGTCAGGAGGTTTCTCGTAAC |
| >CRISPR_I_metagenome_YMBEK03TR-VT-2 |
| ATTCTCTGCCGCAGTGAAAACGATGCTAGAACTTTCCTAG |
| >CRISPR_I_metagenome_YMIAV82TF-VT-1 |
| AGTCCGTAGTATTTTTTATCATCTAGTGGAAAAGGT |
| >CRISPR_I_metagenome_YMIAV82TF-VT-2 |
| TGTTAAGACTCTGACTGATACACAACTTGCTGTATACCTGAG |
| >CRISPR_I_metagenome_YMIAV82TF-VT-3 |
| GTATAGAAAGCACTGTTCGACTTAGTGGCCGAACCATAAGC |
| >CRISPR_I_metagenome_YMIAV82TF-VT-4 |
| TTTAGGCAGTGAGAGACAAGTCTGCCTATTCAAAAAATAA |
| >CRISPR_I_metagenome_YMIAV82TF-VT-5 |
| TTGCTCCAAATCGGAGGTCAATATGTCTATTCCTAAAACTTT |
| >CRISPR_I_metagenome_YMIAV82TF-VT-6 |
| GGAAGAATCTATAAGTCAAAAGAGAAGCTGTCGAATGAT |
| >CRISPR_I_metagenome_YMIAV82TF-VT-7 |
| TAGGTTACTGGGAACTCCACCAAGGTTATGCTGAGCG |
| >CRISPR_I_metagenome_YMIAV82TF-VT-8 |
| CCCGATTTCCTCGCTCGGTTTGGGGTCGGCGATGTGGAC |
| >CRISPR_I_metagenome_YMIAV82TF-VT-9 |
| CTACGACCCCAGTTACACCGGGGATGGCGTTGAACTT |
| >CRISPR_I_metagenome_YMIAV82TF-VT-10 |
| GTGGTTTAGGGACCATCTGGGGCGTAAGAACGCTATCAACC |
| >CRISPR_I_metagenome_YMIAV82TR-VT-1 |
| GCTCAGCGCGCCGGGTACCGTTCTGACCAGTGCCGA |
| >CRISPR_I_metagenome_YMIAV82TR-VT-2 |
| ATTACTGCTTCGAATTCGCAAGGAGTGCTGAGAATCCGC |
| >CRISPR_I_metagenome_YMIAV82TR-VT-3 |
| TCCTGTGTGAAGTCTTCGGAAACGACTTCGTAGTGGT |
| >CRISPR_I_metagenome_YMIAV82TR-VT-4 |
| CCCCACACACCCAGGAGATAATCCTGGGTGTTGTTTATCCCTAGTG |
| >CRISPR_I_metagenome_YMIAV82TR-VT-5 |
| ATGACTAAACTTCGCATAGAGCGTGGGGAGTACCGAG |
| >CRISPR_I_metagenome_YMIAV82TR-VT-6 |
| CCCACATACGTGAGTACAAGCCTTTTCCCGTTGGGCC |
| >CRISPR_I_metagenome_YMJAS63TF-VT-1 |
| CCTGGGTGTCTAGGGGGAGGTTAGGGAATTACTTTA |
|  |
|  |
|  |
|  |
| >CRISPR_IID_OS-B_SP1 |
| GCTTACGTGGCGTGGCATAGAAGTACAACTCATCAG |
| >CRISPR_IID_OS-B_SP2 |
| GCTTTTATTGCAGCGTGTGAGAATAGCAGGTATCATGGTAA |
| >CRISPR_IID_OS-B_SP3 |
| CCCTGGCCAGAGCTATCGAGTTCACCCGCTGGCTGCAGA |
| >CRISPR_IID_OS-B_SP4 |
| TTGCCGGGGAAAATGTAGGCGAACGCCATCAGGGCC |
| >CRISPR_IID_OS-B_SP5 |
| TTTTGGGCTCTCGGGTAATCATGACTACCCCGTCTAACT |
| >CRISPR_IID_OS-B_SP6 |
| TTTACCAATAGTACCTACTGGTCTAGATTGTTTACCCCAA |
| >CRISPR_IID_OS-B_SP7 |
| GCTATCCAGCAAAGTTACCAGTTTCTCCATTTCTTC |
| >CRISPR_IID_OS-B_SP8 |
| AAGATCTGCTTCTCTGCACCACTAGCTCGCTTGGCTG |
| >CRISPR_IID_OS-B_SP9 |
| TCATTGTTTCCCCCTTTCATGCGTGCGTGTGGATAAGTT |
| >CRISPR_IID_OS-B_SP10 |
| GCCCAATATAAATGCCTCTGCTAAAGCCCTCGACTTTGCCATT |
| >CRISPR_IID_OS-B_SP11 |
| AGATGGTGGTGAACCTTGCATGGATCCGCAGTGAGGTTAA |
| >CRISPR_IID_OS-B_SP12 |
| TTGGCAATTAGCAAGTGAACCAATTCAGGATGAACATC |
| >CRISPR_IID_OS-B_SP13 |
| TACCAAACTATCGGAAGAGGGGAGGGATGGCAGTAGTTAG |
| >CRISPR_IID_OS-B_SP14 |
| AGAACTACCTCTGGACTAGCTTCTTTGGTTGCTTT |
| >CRISPR_IID_OS-B_SP15 |
| CAGAAGACGAAGTCTAGAAACAAAACGAGAGGAGTGTA |
| >CRISPR_IID_OS-B_SP16 |
| TCTTGGTACTCGATCTCGGCCCCTTCTAGCCCATCTG |
| >CRISPR_IID_OS-B_SP17 |
| CTTTCTCCGAATGTCCTTTCGGACTTGCGCGTTAAAGCGCTTACG |
| >CRISPR_IID_OS-B_SP18 |
| TGTCTCTGAAATGTTGAAGAATCAGAGACTTTCCAG |
| >CRISPR_IID_OS-B_SP19 |
| AAGGAACCGTCGAAGCCGGCGAACATCTCGATTTCGTCC |
| >CRISPR_IID_OS-B_SP20 |
| CCCTCTTGAACGGTCTGCGAGCTGCCGCTCGTGTGGAGAGC |
| >CRISPR_IID_OS-B_SP21 |
| AGGTGAACTGGTCTTTTTCTCTCACCTTGTAGGTGAGGTTGCTTGTT |
| >CRISPR_IID_OS-B_SP22 |
| GTTGCATTCTTGATTGGTCCCAGAGGAGTATCCTGCC |
| >CRISPR_IID_OS-B_SP23 |
| TACGTTGAATCTCAGGACGTTGAAACTCTTTACATATACGAT |
| >CRISPR_IID_OS-B_SP24 |
| TTTGACACTAAACACTCGCGGATAAACCGCATGGCCTGCA |
| >CRISPR_IID_OS-B_SP25 |
| AAGGGAGGAGTCTGTTATGAACGTTATGAAGTTTATCGG |
| >CRISPR_IID_OS-B_SP26 |
| ACGCGCACGGCGTTTTGCACGATGCGTAAGCATAACCAACCTC |
| >CRISPR_IID_OS-B_SP27 |
| TGCCGAACACGGCCTGGTAGCAGTTTTCTGCTACTTC |
| >CRISPR_IID_OS-B_SP28 |
| TTCCCTCAGGCTATTGCTAACCAGACGTGCCCGAACA |
| >CRISPR_IID_OS-B_SP29 |
| AGCATCTAGCCAGCTCACTTTTGCGAGTATCCGGGCA |
| >CRISPR_IID_OS-B_SP30 |
| TAAGCTTCTTGGTCCCTGGATCTACTACTCCAA |
| >CRISPR_IID_OS-B_SP31 |
| AATCCAGATAATTTCGTTATTCTCGTTGATTGCATACAACG |
| >CRISPR_IID_OS-B_SP32 |
| AACCAGTCCTGGCATTCGCAGGTGAACTGGTCTTTTTCC |
| >CRISPR_IID_OS-B_SP33 |
| ACCAAAAGCTCCCTGGCACTTTGCCGGCGAAACTCGA |
| >CRISPR_IID_OS-B_SP34 |
| CGCAACTCCAAGACAATCTCATACAGGTCATCGTAAA |
| >CRISPR_IIG_OS-B_SP1 |
| GCAATTGCAGGGGTAGAACACCCCCGCATCGTGGAT |
| >CRISPR_IIG_OS-B_SP2 |
| TTGTTCTTTGGTATACTTGGCGTCTAAGTCACTGAGGT |
| >CRISPR_IIG_OS-B_SP3 |
| AGTCTTACCCTCCACCGACTGGTGGTTCTGCTCGCCGAA |
| >CRISPR_IIG_OS-B_SP4 |
| ATCCTGGACCCGGAATACGACGACGAGCCCGTCATTTTTG |
| >CRISPR_IIG_OS-B_SP5 |
| GTAAATCTTTGGGCTCCAAAGTGCCCCCATTACCCACCC |
| >CRISPR_IIG_OS-B_SP6 |
| GTAGAGCTATTAAAGTAACAGCTACAACCTCTCCTGGAA |
| >CRISPR_IIG_OS-B_SP7 |
| CCTTGATTACGGCCTCGATTTGGCTAACCCAACGACGTGCA |
| >CRISPR_IIG_OS-B_SP8 |
| AAAATATAGGTATGTCAATATAGATACAACCATTTGAA |
| >CRISPR_IIG_OS-B_SP9 |
| ATGAGGTGTATAGGCCAAACTCGTTCGATCTATCCTCGCGGG |
| >CRISPR_IIG_OS-B_SP10 |
| TACCACAAGGTGTATACCAATGAAGTAGAGTACCTCGAGC |
| >CRISPR_IIG_OS-B_SP11 |
| ACAAGACATGACTAGCCTCCAAAGGAGTAGATTTTCAA |
| >CRISPR_IIG_OS-B_SP12 |
| ACTTTTAAAACTTTTTTAAAAGATCATCTTAGCTGACCCTT |
| >CRISPR_IIG_OS-B_SP13 |
| CTCTAAGTGCAACCAACTTGCTATATTCCCGTTGTAGGTCT |
| >CRISPR_IIG_OS-B_SP14 |
| ACTGTTCCAAATCAGGAACGTAAAACGAGACGGAAATGTATCC |
| >CRISPR_IIG_OS-B_SP15 |
| TCACCGAGGTTTGAAGATTGGTGTCTCGGTTATTCAGACAG |
| >CRISPR_IIG_OS-B_SP16 |
| TCATCGGTCTCACGCAGACTATTGGTAACCAGCCGT |
| >CRISPR_IIG_OS-B_SP17 |
| CTCAAGGGCGGCTTTATAGCCGTTTCTTTCTACATCC |
| >CRISPR_IIG_OS-B_SP18 |
| AGCCAGCTCTTAGCCAACTGAATTTGTTTGTCATTGAGTT |
| >CRISPR_IIG_OS-B_SP19 |
| CATTGCACAAAGCCCCCGGCGGAGAAGATTTGCATCGC |
| >CRISPR_IIG_OS-B_SP20 |
| TAGATTAGCTGGACCATCCGGAGAGCTTCCCGGATGGT |
| >CRISPR_IIG_OS-B_SP21 |
| AGGATGACTTTGGAGAAATAGCTCAGCTATTTCCAT |
| >CRISPR_IIG_OS-B_SP22 |
| TGTACTACAGTCCTGACAAAGGGCTGTACGAAGGGCCAACGGT |
| >CRISPR_IIG_OS-B_SP23 |
| TTTTTGAAGGAACAGCCAAACCTACAGTGAATTTGCA |
| >CRISPR_IIG_OS-B_SP24 |
| GACGGATCCCTCGTCTCTATATTCTTCCAGCTCTTCTAA |
| >CRISPR_IIG_OS-B_SP25 |
| TTGTTGTCTTGGTTGATCTGGAAAAGTAAGAGGGT |
| >CRISPR_IIG_OS-B_SP26 |
| GTTAGTCCCTGAATAAGGGAGGAGGATGGCATGAATGCAGCTCTA |
| >CRISPR_IIG_OS-B_SP27 |
| TAACGTGTTGAAAGCCGGATGAATCTCCTTCTTCAACA |
| >CRISPR_IIG_OS-B_SP28 |
| ACGAGTACGATACGTTCCATAACAAACCTCCCTTTC |
| >CRISPR_IIG_OS-B_SP29 |
| GATCAAGTCTAACCCAATTCGGGTAGACCGACTGTGTTGCAAG |
| >CRISPR_IIG_OS-B_SP30 |
| TTGGGGATTAGGATCTTCCCTTCCAGGAAGACTTTTTT |
| >CRISPR_IIF_OS-B_SP1 |
| GTATGTCCTAAAACTACCTCGGGGTTGCAGCCGTACTGC |
| >CRISPR_IIF_OS-B_SP2 |
| GTATGTCCTAAAACTACCTCGGGGTTGCAGCCGTACTGC |
| >CRISPR_IIF_OS-B_SP3 |
| CAAGATCCCGCAAAAGCGCGCATCTCTCGGCACGAATGAT |
| >CRISPR_IIF_OS-B_SP4 |
| TACATACTACTGGATATACGGACACCGCCGTGTAAG |
| >CRISPR_IIF_OS-B_SP5 |
| TTGGATCCAGCAAAATCCGCAAAACGGATTGACTAGA |
| >CRISPR_IIF_OS-B_SP6 |
| TGGAATGCCAGTGTCATGCTCTGGCAACGTAGTTTTGG |
| >CRISPR_IIF_OS-B_SP7 |
| TCCTTCACTGACCATACGGTCAGCTCACGCAACACCGACTCCCG |
| >CRISPR_IIF_OS-B_SP8 |
| CTCCGTCTGAGTCAACCGAATACCGCTATAGTCCTTATTCGGGG |
| >CRISPR_IIF_OS-B_SP9 |
| AGGTTCAGTCTTTCCCTGCCTTAGGAGGAAACTATG |
| >CRISPR_IIF_OS-B_SP10 |
| AGCTCACTGATATCCAGCGCGAGCAAATTCGTGCGGCGAA |
| >CRISPR_IIF_OS-B_SP11 |
| GCTCGACAACCTCACATGGGAAGCAATTCCCGGGGGAGTGGG |
| >CRISPR_IIF_OS-B_SP12 |
| GCTACCCTCGTGCGTACACAAAAGCCGCCCATTTTGGC |
| >CRISPR_IIF_OS-B_SP13 |
| TCGGGCTAGAACAGGCAACGGAGGAAGACATAAAAACCATCA |
| >CRISPR_IIF_OS-B_SP14 |
| AGATGTTTGTTACCGTCAATGTAGCAACGAACTTTTTCAG |
| >CRISPR_IIF_OS-B_SP15 |
| GGGAGGAATCATCCAGTAATAGCCGACTTTCTTTTT |
| >CRISPR_IIF_OS-B_SP16 |
| ATTAAAGGGAGGGCTATATGAAGGAACCGGTCATCAA |
| >CRISPR_IIF_OS-B_SP17 |
| AAACCTCCTGAATCTCCAGATCACCCCAAAACTGGGCTAGAAGGT |
| >CRISPR_IIA_OS-B_SP1 |
| CCCGCCTTATTGGCGGGGGATTTTTTGTGGCTATAGG |
| >CRISPR_IIA_OS-B_SP2 |
| AGCTTCGATCCAGCTCACGAGAGCGAGGATACGAGCAACAT |
| >CRISPR_IIA_OS-B_SP3 |
| GGAGTTGGAATGGCAACGCAGGCTAGCAGGGTGTCAA |
| >CRISPR_IIA_OS-B_SP4 |
| GGAGTTGGAATGGCAACGCAGGCTAGCAGGGTGTCAA |
| >CRISPR_IIA_OS-B_SP5 |
| ACATATTGGCGGCAGGGCTTGCCGACCTTAATGGGCAGC |
| >CRISPR_IIA_OS-B_SP6 |
| GATATAGGCATGATTTCTTCCTAAGATAGGAAA |
| >CRISPR_IIA_OS-B_SP7 |
| GTTCCATATCAACTCCTCCCTTATCGGGGAATAAAGAACA |
| >CRISPR_IIA_OS-B_SP8 |
| ACCCAGCGTTACAGGCCTTTTCCACGGCTGCTGCCGCT |
| >CRISPR_IIA_OS-B_SP9 |
| ATGGTACTGGCAAACCTGGAACGGTTTGCCAATCAA |
| >CRISPR_IIA_OS-B_SP10 |
| CCAAGCGAAAGGTGCCGAGGAACTCTCAGAAGTGGAC |
| >CRISPR_IIA_OS-B_SP11 |
| CTACCAACCTGGGTACCCGCGCTCCCGGAGACTCAACCGT |
| >CRISPR_IIA_OS-B_SP12 |
| TCGGTGTACCAGGTCACAATTTTCCGTCACCTAGAAA |
| >CRISPR_IIA_OS-B_SP13 |
| GGTCAGCTGGATAGAAGCTGGCCGGTTCATCCGATCTC |
| >CRISPR_IIA_OS-B_SP14 |
| TTAATCGTCTACACTACATGATCCGGCTCCTCGTCACCGCACGGCA |
| >CRISPR_IIA_OS-B_SP15 |
| GGGGGAGGTTGGTTGGGAGGTTGGTGGGAGTTAGACT |
| >CRISPR_IIA_OS-A_SP1 |
| ACTGCTAAAACCTTTGCCGCTCTCGGAATGCCGGGAGCG |
| >CRISPR_IIA_OS-A_SP2 |
| TCAGGGGATCCAGATCATCCCTGGAACACAGCAGGGGGAGTTTCCAAA |
| >CRISPR_IIA_OS-A_SP3 |
| AATATTGCTCACCGTAGGTGGCATGGAAGATCCAAGGAC |
| >CRISPR_IIA_OS-A_SP4 |
| CCGATCTTCTTGGATCGGATTCCGGCTGGGAGTCTCAAAGGG |
| >CRISPR_IIA_OS-A_SP5 |
| TTGCAGTGATGCGCAAGGCGCGCCAAGCTGCCAAAGAT |
| >CRISPR_IIA_OS-A_SP6 |
| GTGTAAAATGAATCGCGCTTTCCTCCGTCTGTTGGTCAAGCGT |
| >CRISPR_IIA_OS-A_SP7 |
| CTGAGAATACTCCAGGGGGAGCAACCCCTAGCCTAATC |
| >CRISPR_IIA_OS-A_SP8 |
| TTCGTACCTCGTTTAAGGGAGTTTGTGCCCATGAAC |
| >CRISPR_IIA_OS-A_SP9 |
| CAAATGGGCGGTGATTTCGACGGTGACCGCGTAAGCGT |
| >CRISPR_IIA_OS-A_SP10 |
| GCTTTGGTTGGATGACAGAAATGATTCCGGGGCCTTATC |
| >CRISPR_IIC_OS-A_SP1 |
| CATCCGGGATGTTGCCCGGATACTCGGGCGGGTCCATC |
| >CRISPR_IID_OS-A_SP1 |
| AATGGAGTTACGTGGTTCCAGAATAAGCGGCTGGAGA |
| >CRISPR_IID_OS-A_SP2 |
| AAGCAAACAACTAACTTTCGCAAGAACTTGGCGTTCATAGTAAT |
| >CRISPR_IID_OS-A_SP3 |
| TCCCAAGGGTACTACAGTTACTATTTCGCAGTTGCGCAAGG |
| >CRISPR_IID_OS-A_SP4 |
| TTTGCTGGGTCTATCGAAACAGATTCACTGGATAAAC |
| >CRISPR_IID_OS-A_SP5 |
| TGTATCTGGGCATACGGTGGGTGGTACATTTCGGAGC |
| >CRISPR_IID_OS-A_SP6 |
| CTCATAGTGCGGGATGTAGGCGGTAAGCGCTTCTACT |
| >CRISPR_IID_OS-A_SP7 |
| CCTTTTTTAACAACTTCCTTAACTAGAGCAGATGTTTACA |
| >CRISPR_IID_OS-A_SP8 |
| AGCATTACTGAGCTGCGCAAAGCGCAAAAGCGTGAGCG |
| >CRISPR_IID_OS-A_SP9 |
| TCTAAACAAAGGAGTCCGTATGAACACATTCAAGATT |
| >CRISPR_IID_OS-A_SP10 |
| TGATGGTATACCCAGTCCTGACTGGGCTACATCCTGCC |
| >CRISPR_IID_OS-A_SP11 |
| TTCTGGCGGGATCACCCACACACCCCTGTACTACA |
| >CRISPR_IID_OS-A_SP12 |
| CCGATTCGTTCGCAAGCGACTGGTTACTAACAGTCTTTAC |
| >CRISPR_IID_OS-A_SP13 |
| ATTAAGTATAAGACAGAGCAGCATCCTATTTTTCGCACTAATA |
| >CRISPR_IID_OS-A_SP14 |
| TCGACACGGCCGCCACGAATAAACCAAGGACTCAG |
| >CRISPR_IID_OS-A_SP15 |
| TTACCGTCCAGTACATATACATCCCGCACTTCAAGAAGAG |
| >CRISPR_IID_OS-A_SP16 |
| ATCCTGCTGATATGGTAGAAGGCTTGGTAGCTCCTCAAT |
| >CRISPR_IIB_OS-A_SP1 |
| TATAACGCTCGGCAGATCGCTGGGCGTTGAGAGCC |
| >CRISPR_IIB_OS-A_SP2 |
| GCAATCGGGCGAGCGTTTGGATAGTCGGCAACTTCGCTGT |
| >CRISPR_IIB_OS-A_SP3 |
| TTTGCCGGTAGCTTTGGACTCATGGTTTCCTCCTTTCTGTAG |
| >CRISPR_IIB_OS-A_SP4 |
| TTTAGCCATCCATCGTATTCCTCAGGAATACTAGGGC |
| >CRISPR_IIB_OS-A_SP5 |
| CTCAACGATGGCATAACGGAGCTCATAGTAATCTCCGCAA |
| >CRISPR_IIB_OS-A_SP6 |
| GCCCAGGGAGTTTAAGAACTCCCGGGCTTGCTTTAGCCACGCGTCCTCA |
| >CRISPR_II_metagenome_CYPF327TF-VT-1 |
| TTTCTACACGCCTAAGGGAGCACATTCCTAGTGGGCTGACG |
| >CRISPR_II_metagenome_CYPKX69TF-VT-1 |
| AGGCACAATCTAGCTGCGCTTGGAACGCAGCATCTAGAATGTTTCCAA |
| >CRISPR_II_metagenome_CYOBD48TF-VT-1 |
| TTTCATGCTGGGCTTTGCCCAGTGATGTGTTGTTTCGGAGGTC |
| >CRISPR_II_metagenome_CYPB048TR-VT-1 |
| TTCATGCTGGGCTTTGCCCAGTGATGTGTTGTTTCGGAGGTC |
| >CRISPR_II_metagenome_CYPB460TF-VT-1 |
| ACGACGCGAGGGCGCGGGGGCCTCACGCGCCGGCGC |
| >CRISPR_II_metagenome_CYPBR81TR-VT-1 |
| CAGCTCTGGATCAGGATGCCGGTAGTGGTACACCCAAGTT |
| >CRISPR_II_metagenome_CYPBR81TR-VT-2 |
| CTTTCGACCTTAGCCGCCACCGGCTGGTGGTGCTCGCCGAACTGGCA |
| >CRISPR_II_metagenome_CYPDD55TF-VT-1 |
| CTATTTAAGAATTGCCCAAGAATCTTTCTCCCAATTG |
| >CRISPR_II_metagenome_CYPDZ43TR-VT-1 |
| ATTCAAACCATTTTCTTCCCTATGAATATAAGTTGTATAGAGTTC |
| >CRISPR_II_metagenome_CYPDZ43TR-VT-2 |
| ATTCCACAACTCTGACTTGGTTGTTGGACAATGATCCAGTGT |
| >CRISPR_II_metagenome_CYPE865TR-VT-1 |
| ACTCCTGCTCGTCTGCTATCATGACTCTCGTAATAATGGGAAAGTTCCCCC |
| >CRISPR_II_metagenome_CYPE865TR-VT-2 |
| GGGCTGGGAGCCGACATCACTACGCCATCGTTCAGCTTGTTCCCCC |
| >CRISPR_II_metagenome_CYPEK54TR-VT-1 |
| CGCGAAAGCCTTATGCCATAAGGGTTAGTGACATCAG |
| >CRISPR_II_metagenome_CYPIX94TF-VT-1 |
| CTTGCCAGTACGCTCCAAACACCGGCTCAGGATGGGGAT |
| >CRISPR_II_metagenome_CYPKD46TR-VT-1 |
| GAGTGCAATTTGTTTGTGGTTCATTTTATCCTCCTT |
| >CRISPR_II_metagenome_YMBB565TF-VT-1 |
| CTCAACGATGGCATAACGGAGCTCATAGTAATCTCCGCAAT |
| >CRISPR_II_metagenome_YMBB565TF-VT-2 |
| GCCCAGGGAGTTTAAGAACTCCCGGGCTTGCTTTAGCCACGCGTCCTCAA |
| >CRISPR_II_metagenome_YMIAX35TR-VT-1 |
| CTTGCCAGTACGCTCCAAACACCGGCTCAGGATGGGGAT |
| >CRISPR_II_metagenome_CYPAA48TF-VT-1 |
| TAGTATAAACAGGCTTACGCCTGTTTTAGTTGTTAA |
| >CRISPR_II_metagenome_CYPAA48TF-VT-2 |
| TATGGATCAAATGGCATAACAATCATATATTGTTCC |
| >CRISPR_II_metagenome_CYPC672TF-VT-1 |
| CTTCTTGAGGGATTCCCTTACGACATGGACGGAGC |
| >CRISPR_II_metagenome_CYPC672TF-VT-2 |
| AATCTAACGTTATCGAAGTCCTCAAGAAAGAAGTCCA |
| >CRISPR_II_metagenome_CYPCW50TF-VT-1 |
| TTCGCTCTATGCGCAGTTTGACAATACTGCGAAGACACTTGG |
| >CRISPR_II_metagenome_CYPCW50TF-VT-2 |
| AACAACATTCAGGGGTACTTTGACTTCCATAACTAACC |
| >CRISPR_II_metagenome_CYPFC31TF-VT-1 |
| ACCCCGCCTTATTGGCGGGGGATTTTTTGTGGCTATAGG |
| >CRISPR_II_metagenome_CYPFC31TF-VT-2 |
| ACTGCTCTTTTCAGAGCGTAGGGTGCTATTGGCGTATT |
| >CRISPR_II_metagenome_CYPFC31TF-VT-3 |
| ACTGGTCCCAGTGACGTCTAATGCTGCTGATAAAGGCGGTA |
| >CRISPR_II_metagenome_CYPG005TF-VT-1 |
| AACGTCAGTATTACTAGGCTAAGCTCAACTATGGCAGTAAATAGCGTTTCTAATT |
| >CRISPR_II_metagenome_CYPG005TF-VT-2 |
| AACCACAACTTCAGTGAACAACAGTACCAAATCGTCGAAATGTTTCCAATC |
| >CRISPR_II_metagenome_CYPI813TR-VT-1 |
| TTGATCGATGAGGATCGGATCCCGCCAGAGTTTGAGATTTCCAATGTCC |
| >CRISPR_II_metagenome_CYPI813TR-VT-2 |
| GCTGCACAGGCGCGTGAAGTTTGGGAGCACCTGGCGGTTGTTTCCAATTTCT |
| >CRISPR_II_metagenome_CYPI813TR-VT-3 |
| ATGATGCGGAAGGCCCGCAAGGCTGCTGAACAGCTGCGTTTCCAATTTCT |
| >CRISPR_II_metagenome_CYPIP72TF-VT-1 |
| TTATTAGCATCGTTCGCTATTGTCATAGATGCAGGTAC |
| >CRISPR_II_metagenome_CYPIP72TF-VT-2 |
| AATTCTCATCCCACCTTAAGCACTCGTAACTGGAACA |
| >CRISPR_II_metagenome_CYPIP91TR-VT-1 |
| TTGGAAACACGGGTGCTACAATGACAACACGCATTCCAGGAGT |
| >CRISPR_II_metagenome_CYPIP91TR-VT-2 |
| CTGGAAACATCACTCGTCCTTTGCCCAAAATCCTTGTCGAAAAG |
| >CRISPR_II_metagenome_CYPL122TF-VT-1 |
| TTTAATAAGACCGTGAGAAAGAACCTCCATTCAAAAGT |
| >CRISPR_II_metagenome_CYPL122TF-VT-2 |
| GGGAAACGTGGGACATAACTATAAAGTAGACTAACCCC |
| >CRISPR_II_metagenome_GYRAT76TR-VT-1 |
| AATTGGAAACAAGGTTGATGCGGAGACCTTATATTTCGCATTCAAA |
| >CRISPR_II_metagenome_GYRAT76TR-VT-2 |
| AATTGGAAACGTGTGATGGGGGAGGAACTAGATGGTACTACCCATTATGGC |
| >CRISPR_II_metagenome_GYRAT76TR-VT-3 |
| AATTGGAAACGCCCAGGGAGTTTAAGAACTCCCGGGCTTGCTTTAGCCACGCGTCCTCAA |
| >CRISPR_II_metagenome_YMBE750TF-VT-1 |
| CACGGATGACCCCACCATCCAGTGTGAAGTAGAAGGCG |
| >CRISPR_II_metagenome_YMBE750TF-VT-2 |
| CATGTTCCTCCTTTCCCCAGCAGGGGGCACTGGTCAA |
| >CRISPR_II_metagenome_YMBEE53TR-VT-1 |
| TTTCATGCTGGGCTTTGCCCAGTGATGTGTTGTTTCGGAGGTC |
| >CRISPR_II_metagenome_YMBEE53TR-VT-2 |
| CCAGACGTTTTACGTGGTGGAAGTCTCCACCCGAGGCGGG |
| >CRISPR_II_metagenome_CYOBH37TR-VT-1 |
| AGTTACTGTCAGCTAGTGTAACCTCCACCTAACTCTC |
| >CRISPR_II_metagenome_CYOBH37TR-VT-2 |
| TGGGGAGCAAGATAGGCTTTCCTATCTTTTACTGCC |
| >CRISPR_II_metagenome_CYOBH37TR-VT-3 |
| TAAATTGTACGACTATTGAGCATTCTGTGTGAAGTTTCAAAT |
| >CRISPR_II_metagenome_CYOBH37TR-VT-4 |
| CTGGGCGTTTCGGCTCCACCTTCCACGGGTAAGGTTGGGTC |
| >CRISPR_II_metagenome_CYOBH37TR-VT-5 |
| GAGTCTTTAGAACTTTGGTAAGTTCTTTCGACTTCCTT |
| >CRISPR_II_metagenome_CYOBH37TR-VT-6 |
| TTGAGGATGTCCTTCAGTCGGGAGTCCGACTCTCGTT |
| >CRISPR_II_metagenome_CYOBH37TR-VT-7 |
| CGAGTCGCATATGCTCGATCTCTATGATTGGGACGAGTACCGCAG |
| >CRISPR_II_metagenome_CYOBH37TR-VT-8 |
| GGGATTAACTCGGTTTGTTCGCATTGTGCCCTCCTT |
| >CRISPR_II_metagenome_CYOBH37TR-VT-9 |
| GGGACAGTGGCTTTCACTCCGAAGTGTTTGAGCTGCTT |
| >CRISPR_II_metagenome_CYOBH37TR-VT-10 |
| TCAGTATTCCCTGCCAATTAGGAGGTTGTTGTGACCAAGCC |
| >CRISPR_II_metagenome_CYOBH37TR-VT-11 |
| TGGGGGAGGGCTAGATTAGACAACCTGTAGCCCAATCGG |
| >CRISPR_II_metagenome_CYOC015TF-VT-1 |
| TGGTTAATCCTAGAGGAGTACTGGACCGATCTTGAA |
| >CRISPR_II_metagenome_CYOC015TF-VT-2 |
| AACAGTCAATCCAGTTTGCATAGTAGCAAACTGCAACTACATTAC |
| >CRISPR_II_metagenome_CYOC015TF-VT-3 |
| AAGGTACAGGACGTTGGCTGAGATCGGGTTTATTTACG |
| >CRISPR_II_metagenome_CYOC015TF-VT-4 |
| GGTGCTTTTCCTCCCTGTTTAAGGGTGTAGTTTGTCCCTGAA |
| >CRISPR_II_metagenome_CYOC015TF-VT-5 |
| CATAAAGGCACAATCTAGCTGCGCTTGGAACGCAG |
| >CRISPR_II_metagenome_CYOC015TF-VT-6 |
| TCCCTTGTGTGGGCAGAAAGAACACCCAGGAGAAATACCTGGG |
| >CRISPR_II_metagenome_CYOC015TF-VT-7 |
| TTAAATCTAACTCAGTTCCATAGGTGTAGAGTTATCTT |
| >CRISPR_II_metagenome_CYOC015TF-VT-8 |
| GCGAAGGTGCAGTCCTACCAACACGGCAACCGGCGT |
| >CRISPR_II_metagenome_CYOC015TF-VT-9 |
| ACTACTACCGCAGCTTTTCCTGGAGCTATTGGCTTTGATA |
| >CRISPR_II_metagenome_CYOC015TF-VT-10 |
| CCCTGTGAAGTAGACGAGGATGAGGCACTTGCGGAATTGAAAG |
| >CRISPR_II_metagenome_CYOC015TF-VT-11 |
| TTCAAGTAGAGACGCCGGCTACCGTAAATGTATAGA |
| >CRISPR_II_metagenome_CYOC015TR-VT-1 |
| TACACCATTTGGTAGGGGTTGGAGGTTAGTATGTAGAG |
| >CRISPR_II_metagenome_CYOC015TR-VT-2 |
| TCTTCAGGGTTCAGGTAAATGTCGTTACCTTCGGTGA |
| >CRISPR_II_metagenome_CYOC015TR-VT-3 |
| ATGGTACCCAAATCCGCCTTGGGCGGATGAGTTGTA |
| >CRISPR_II_metagenome_CYOC015TR-VT-4 |
| ATTCAACACAACGAGTGGGCATGCCCTGGCATGCTAAT |
| >CRISPR_II_metagenome_CYOC015TR-VT-5 |
| TAGCAGTAATATGGAATTAGTTGTATACTCAATACTCATAA |
| >CRISPR_II_metagenome_CYOC015TR-VT-6 |
| TTCCACTTAGCAAGTTTAGCATACTCTCGTTCTACATCAT |
| >CRISPR_II_metagenome_CYOC015TR-VT-7 |
| TTGTATGGTGAGGTCTATGTACGAAGAATTAAGTAAAG |
| >CRISPR_II_metagenome_CYOC015TR-VT-8 |
| CTAGAGGAGTACCTATCCGAGTGTTCGCTCACAAAGCAG |
| >CRISPR_II_metagenome_CYOC015TR-VT-9 |
| AATATACAATCCGCAACTAAAAATAAATTTGGTTCTA |
| >CRISPR_II_metagenome_CYOC015TR-VT-10 |
| AAGGCAAGAAAGCACCTATGTGCATTCCCCATGCTTCTAG |
| >CRISPR_II_metagenome_CYOC015TR-VT-11 |
| ATACACCCAAACCAATCCTGCTAGGCAGACACCTTGTA |
| >CRISPR_II_metagenome_CYOCB32TF-VT-1 |
| CGTAGTAGTCATGTAATCCTCCTGTAGGCAGGGAAT |
| >CRISPR_II_metagenome_CYOCB32TF-VT-2 |
| CATAACTACCTCCCTTGTTACGGGATAAAGAACACCCAG |
| >CRISPR_II_metagenome_CYOCB32TF-VT-3 |
| TCCTGGGTGTTTTGGAGGGGGAGGTCAAACTACTCC |
| >CRISPR_II_metagenome_CYOCB32TF-VT-4 |
| TTCTTTCTTATCCCGAAAAGGGAGTGCGGCCTATGGAAG |
| >CRISPR_II_metagenome_CYOCB32TF-VT-5 |
| TTGACCATCGGATGCGCATAACTACCTCCCTTCTTCAG |
| >CRISPR_II_metagenome_CYOCB32TF-VT-6 |
| TTATATTAGCTATTGGCCCACTCAAAATCGGGCACACGGA |
| >CRISPR_II_metagenome_CYOCB32TF-VT-7 |
| GCGATAGCCGGACCCACTTCGGTGGTCCAGAAACTAAGCAA |
| >CRISPR_II_metagenome_CYOCB32TF-VT-8 |
| GCCAAATTTCCTTAGGTATCTCTCTCTCACAGTCAA |
| >CRISPR_II_metagenome_CYOCB32TF-VT-9 |
| AACCGAAAGTTTTGGCCAATTGACGCAGGAATTTAAT |
| >CRISPR_II_metagenome_CYOCB32TF-VT-10 |
| ACTAGTTATTAGCGGCTATTGGCCCATTCGTAATCGGGCA |
| >CRISPR_II_metagenome_CYPCN89TR-VT-1 |
| TACATTGGCGGGTGGTTAATCCTAGAGGAGTACTGGA |
| >CRISPR_II_metagenome_CYPCN89TR-VT-2 |
| CCCTGCCTATAGGAGGTTTCAATGGCTATCTTCTACGGAA |
| >CRISPR_II_metagenome_CYPCN89TR-VT-3 |
| TATATAAGGAGTGCCTGAGGTAGGCACTCCTTATATTTT |
| >CRISPR_II_metagenome_CYPCN89TR-VT-4 |
| CCACCGTAACCAACGGTGGACGCGCCTCACAAAGAGGTGC |
| >CRISPR_II_metagenome_CYPCN89TR-VT-5 |
| TTCCTCAATGAGGACTTCCTCAGGCACTGCACACACCGTG |
| >CRISPR_II_metagenome_CYPCN89TR-VT-6 |
| TCCAGCATTTTTTTACAGAGGAGTATTAAAACAATTTT |
| >CRISPR_II_metagenome_CYPCN89TR-VT-7 |
| ACTTGTGACCGACCGCTTCAGGTTTGTGTAGGTTT |
| >CRISPR_II_metagenome_CYPCN89TR-VT-8 |
| ACCAACCGACCGATGCTCGCGGCTACTTCACTCGG |
| >CRISPR_II_metagenome_CYPCN89TR-VT-9 |
| TTTGGCAACAGCGTAGGAATTTGCTCTGGCTTAGCCTCAGTCA |
| >CRISPR_II_metagenome_CYPCN89TR-VT-10 |
| TACAAGACTTAGTTATAAGATCAATCCAACAATTGATTTAT |
| >CRISPR_II_metagenome_CYPEG04TR-VT-1 |
| AATATTGCTTTGACCCAGATGCTGCTGAACCGATC |
| >CRISPR_II_metagenome_CYPEG04TR-VT-2 |
| CCTCATGAGAAGAACACACCCGTCGACGCCGAGATTCTCGGCAA |
| >CRISPR_II_metagenome_CYPEG04TR-VT-3 |
| TAGTTATCCCACGAACCGTACAGCGTGATATGCATG |
| >CRISPR_II_metagenome_CYPEG04TR-VT-4 |
| GCCAGGAGTTTTGCGATAGCACGCAGATGCGCGCACTTG |
| >CRISPR_II_metagenome_CYPEG04TR-VT-5 |
| ACGAAGGTACATGCGCTTGCTACCATAAATATAACGAATCCC |
| >CRISPR_II_metagenome_CYPEG04TR-VT-6 |
| TTTCACCGGTATAGGAACGTGGGAGACTTCCTTCAGGCC |
| >CRISPR_II_metagenome_CYPEG04TR-VT-7 |
| TTTCACCGGTATAGGAACGTGGGAGACTTCCTTCAGGCC |
| >CRISPR_II_metagenome_CYPEG04TR-VT-8 |
| TTTCACCGGTATAGGAACGTGGGAGACTTCCTTCAGGCC |
| >CRISPR_II_metagenome_CYPEG04TR-VT-9 |
| TCCCGTACCACCAGAGGCTTAGGTTCGACAACTGGCTTGGA |
| >CRISPR_II_metagenome_CYPEG04TR-VT-10 |
| AGCAATATTCAACGACGATACTAGACCGGCCTACAC |
| >CRISPR_II_metagenome_CYPEG04TR-VT-11 |
| CATTACCGCAAACCGTTGAGCGGAGAACGTACCGTAGTCTA |
| >CRISPR_II_metagenome_CYPFX41TF-VT-1 |
| TTGTTCTACACGGGCTTGCTGTTCGATGCGCTGCCTTTCAA |
| >CRISPR_II_metagenome_CYPFX41TF-VT-2 |
| GAGAAGGTGGTAGAAGACCGCGTAATACTGCTCGGCG |
| >CRISPR_II_metagenome_CYPFX41TF-VT-3 |
| GATGCAATGTTTGTAAGAGCTTTCCAAGACTGCTTTAGA |
| >CRISPR_II_metagenome_CYPFX41TF-VT-4 |
| TCTATTGTTTACTACAGCTGATCGGGAGGTAGCAGA |
| >CRISPR_II_metagenome_CYPFX41TF-VT-5 |
| CAAAGCTGCTATGATTGCAGATAGGGAAGTTAGAATCTTACCTCG |
| >CRISPR_II_metagenome_CYPFX41TF-VT-6 |
| AGCCAACGCACGGGCGCGTTTCCGTTGGCGATTCGT |
| >CRISPR_II_metagenome_CYPFX41TF-VT-7 |
| TGGTATAGCGCATATATTCCTCCCTTTCGGGTAGAGAAAGAAC |
| >CRISPR_II_metagenome_CYPFX41TF-VT-8 |
| AAGTCTTTAAAGAAGAAGACTGTCTCACTCTCCAGGAA |
| >CRISPR_II_metagenome_CYPFX41TF-VT-9 |
| ATCTTCCAGGACGGCTTCATCTGCGGTGAGACACGTGTAAA |
| >CRISPR_II_metagenome_CYPFX41TF-VT-10 |
| TACTCCACATCGTGGAAATCTCTGTTAAGGGCGGGTTCAAAT |
| >CRISPR_II_metagenome_YMAAP30TF-VT-1 |
| CCCGGGATTTTCACCCCGCCATAATATGGATAGTTCCA |
| >CRISPR_II_metagenome_YMAAP30TF-VT-2 |
| GAGTAAATCTGCTGGGCCAGAGCTTCTGCGCGACGACG |
| >CRISPR_II_metagenome_YMAAP30TF-VT-3 |
| CTGAATCGCAACGGGGTGTGGGTAATCCCGCCGGTAA |
| >CRISPR_II_metagenome_YMAAP30TF-VT-4 |
| TTCCACGTAGAATGCAATTGCGGAAGTACTCCTTTTTGT |
| >CRISPR_II_metagenome_YMAAP30TF-VT-5 |
| GGTTACCAGGTAATCACCCAGCGCCCTTTCCCCCATT |
| >CRISPR_II_metagenome_YMAAP30TF-VT-6 |
| GAGTGAAGCTAACTCCATCCGGGAGTTCCGTTATTTT |
| >CRISPR_II_metagenome_YMAAP30TF-VT-7 |
| TCGGTGATTTGAACACGCTTTTTGAAGTTCGGGATATAGAT |
| >CRISPR_II_metagenome_YMAAP30TF-VT-8 |
| TCCAGAGCCCCAAACCTGGGGGTACCCAGATTAAGTATTC |
| >CRISPR_II_metagenome_YMAAP30TF-VT-9 |
| TTCAAGCTCTCGGTGTGAACCTCGAGCTCCCCGGA |
| >CRISPR_II_metagenome_YMAAP30TF-VT-10 |
| AATTGTTGTTCTAACCCAACCGCAGCCTTGTTTATCAAGACT |
| >CRISPR_II_metagenome_YMAAP30TF-VT-11 |
| AACCATAGGCCCCGGGGTGTATACTCAACGAAGTCCC |
| >CRISPR_II_metagenome_YMABI04TR-VT-1 |
| GCCACCGCCCATTCGAAGTGAGGTACTCCCTCAAA |
| >CRISPR_II_metagenome_YMABI04TR-VT-2 |
| AATGATCCCGATATTAAGTTACTTATTACTAATACAGAG |
| >CRISPR_II_metagenome_YMABI04TR-VT-3 |
| GCCACCGCCCATTCGAAGTGAGGTACTCCCTCAAA |
| >CRISPR_II_metagenome_YMABI04TR-VT-4 |
| AATGATCCCGATATTAAGTTACTTATTACTAATACAGAG |
| >CRISPR_II_metagenome_YMABI04TR-VT-5 |
| AATCAGCCCTTTAGCTGGGCTGAGGTTGAACACCCACTCGTGA |
| >CRISPR_II_metagenome_YMABI04TR-VT-6 |
| TATCTCCTGGGTGTTTTGGTTGGGGGAGAGCGTTAGAGG |
| >CRISPR_II_metagenome_YMABI04TR-VT-7 |
| GTGAATAATTGTGACTACGAGCGCTGCCAGTGGCG |
| >CRISPR_II_metagenome_YMABI04TR-VT-8 |
| CCCAGTCGATAGGAGCAGAGGCTTTCTGCTCCCACTCT |
| >CRISPR_II_metagenome_YMABI04TR-VT-9 |
| TTTCATACGGATTATAGTAGTATCTGGTTACCTGGGG |
| >CRISPR_II_metagenome_YMABI04TR-VT-10 |
| TTTCACCTGGGTGTTGGTGGGAGAGGTTATGTTAGATCGTTACT |
| >CRISPR_II_metagenome_YMABI04TR-VT-11 |
| GTGGTCCGAATTGACCACGGGCAAATGTGCCCACTGAGAG |
| >CRISPR_II_metagenome_YMJAG38TR-VT-1 |
| CCGGTATTGGTCTCCGTAGATGTCCCAGTCGTAGAAGTCGA |
| >CRISPR_II_metagenome_YMJAG38TR-VT-2 |
| TTCAGGCGAAACTGAACGGGGCAGCCCACGAACACTTTCAG |
| >CRISPR_II_metagenome_CYPC946TR-VT-1 |
| CATCGAGCCAGGCTAGCCAAATAGGCCTAGCCTA |
| >CRISPR_II_metagenome_CYPC946TR-VT-2 |
| CAGTTGGAGTCACCGAGGGTAGCGATATCTACCTG |
| >CRISPR_II_metagenome_CYPC946TR-VT-3 |
| AAAGCGCAGCGCGCTGAGCGTCGGCGGAAAAAGTTC |
| >CRISPR_II_metagenome_CYPFR06TF-VT-1 |
| ACGCATTACTGCGGCGGAAGGTGAGCCCCAAGTTC |
| >CRISPR_II_metagenome_CYPFR06TF-VT-2 |
| TAGAACCATCAGGAATGGTAGCCCGTGTCGATAGTAAG |
| >CRISPR_II_metagenome_CYPFR06TF-VT-3 |
| GAATATGAACAGCACTCCGGCTGTGGCCGACAGCTTCTACCTACT |
| >CRISPR_II_metagenome_CYPG116TF-VT-1 |
| GACGCGTCTAACGAAACGTTGTCCATTACTGTAAACATACCA |
| >CRISPR_II_metagenome_CYPG116TF-VT-2 |
| GGTTGTGATGTTTACGCATCGTCAGCAAAAACGAATGA |
| >CRISPR_II_metagenome_CYPG116TF-VT-3 |
| AGCGCTGATTTGTTTGGCTAGAGCGAAGGCTCTTTTCT |
| >CRISPR_II_metagenome_CYPGC56TF-VT-1 |
| ATGTAGTCGCTACCAAGGACTCGCAAAAAGTACCAGATCGA |
| >CRISPR_II_metagenome_CYPGC56TF-VT-2 |
| CGTTGCCGCAGAGCCTCAATTTTCAGCCACTCACGAGCGGCCT |
| >CRISPR_II_metagenome_CYPGC56TF-VT-3 |
| TGACACCGTTACACCCGCACCTTTGGGGATTTGAATCCT |
| >CRISPR_II_metagenome_CYPJG56TR-VT-1 |
| GAACACTACGTTCATCAATTTGACACCACACGAAATCAATGAAGT |
| >CRISPR_II_metagenome_CYPJG56TR-VT-2 |
| GAACGCCGTACGCAAAGCGATGGTGAAGCGGTCCAAAGCAC |
| >CRISPR_II_metagenome_CYPJG56TR-VT-3 |
| GAACTTAAAGCCTGGCAAGGACTTTGGACGCATTCTAGAT |
| >CRISPR_II_metagenome_CYPJP15TF-VT-1 |
| GTGATATAGCTCTTTACCCGCTGCTTGCGCTCGATAGCC |
| >CRISPR_II_metagenome_CYPJP15TF-VT-2 |
| TCCTTGAGTGTCTTGAAGCCTAGGTGGAACAAAACTCTG |
| >CRISPR_II_metagenome_CYPJP15TF-VT-3 |
| TCCCTAGGACGTCTTTGGATCCCTTGAGCTTTCCTTGA |
| >CRISPR_II_metagenome_CYPK760TF-VT-1 |
| TTCTTGCCATTGAGGTTCTTAACGACCAATCGAGGTGGCTC |
| >CRISPR_II_metagenome_CYPK760TF-VT-2 |
| TTAATTTGCGACCGCCAACACCATCGCTGGTCCACACTT |
| >CRISPR_II_metagenome_CYPK760TF-VT-3 |
| GAGTTTTTCAACACTGTAATACGCTGCAATCGCAAC |
| >CRISPR_II_metagenome_CYPKX50TR-VT-1 |
| GGGAGTGGAGCCAACACCCCGCCGATGCCCTGGTAGTC |
| >CRISPR_II_metagenome_CYPKX50TR-VT-2 |
| CCCCGTCACCGCCAACCAGGGCATCTGTTCCAGTTCTT |
| >CRISPR_II_metagenome_CYPKX50TR-VT-3 |
| TACTTAAAGCCACCCTTGACCGATACCTCTACAATGT |
| >CRISPR_II_metagenome_YMAAA56TR-VT-1 |
| TTGAGGACGCGTGGCTAAAGCAAGCCCGGGAGTTCTTAAACTCCCTGGGC |
| >CRISPR_II_metagenome_YMAAA56TR-VT-2 |
| GTTATGGCTGGTGGCAGTTGCTGCCATCACCAAGGGATCCT |
| >CRISPR_II_metagenome_YMAAA56TR-VT-3 |
| TACTAATCTGATTGAGAATGTACAGACGTTTTTTAA |
| >CRISPR_II_metagenome_YMAAA56TR-VT-4 |
| CACAAGGTCAAAGCGGCAGTAGAAGAGCTTCGTAAGAGACTCCA |
| >CRISPR_II_metagenome_YMABK08TR-VT-1 |
| ATACTTCATACCAAACCTCCCTTACGGGGTAAGAAAGAA |
| >CRISPR_II_metagenome_YMABK08TR-VT-2 |
| TTACAACCCCTATGAAAAGGGGGAGTTTATCCAGCATACA |
| >CRISPR_II_metagenome_YMABK08TR-VT-3 |
| GAGGAGGGGTGGCTCCCACCTGAGTGGAGAGATCAT |
| >CRISPR_II_metagenome_YMABK08TR-VT-4 |
| TATTCTATTGTAAACTAGAGGATCTCACACAAGATGGGT |
| >CRISPR_II_metagenome_YMABK08TR-VT-5 |
| GCGGATCTGCATGATGGGGTGGGCAATCGAAGCTG |
| >CRISPR_II_metagenome_YMABK08TR-VT-6 |
| TTTTTTGACTTGACCCAGAGGGGTCGGCTACCTGCCCGAGAG |
| >CRISPR_II_metagenome_YMABK08TR-VT-7 |
| CTTCCTGTTTTCTATCTTAGATAGGTTATTTATTC |
| >CRISPR_II_metagenome_YMABK08TR-VT-8 |
| AAGGGAGGTTATATGTACAAGTTGGCTGGTCGTAAT |
| >CRISPR_II_metagenome_YMABK08TR-VT-9 |
| CGTAGTCTCCCAGATCCTGTTCAAAACGGAGTCACTGGATAACTT |
| >CRISPR_II_metagenome_YMABK08TR-VT-10 |
| GGTGAACACCCTATCTAAATGGACCACACCATACTGGCAC |
| >CRISPR_II_metagenome_YMACF06TF-VT-1 |
| CAGGCAGCGGATCCAGACTACGGCTGGACCGTTGAC |
| >CRISPR_II_metagenome_YMACF06TF-VT-2 |
| TTGTCGTAAAATACCCCCTGTACTTAGTTCAACTGAT |
| >CRISPR_II_metagenome_YMACF06TF-VT-3 |
| TATTAAGGAAGATATCTAACCTCCCACCTAAA |
| >CRISPR_II_metagenome_YMACF06TF-VT-4 |
| GGTTTGTTGTTCCCTGCCTTAGGAGGAAACTATGTCCACTA |
| >CRISPR_II_metagenome_YMACF06TF-VT-5 |
| GCCAAGCAAATTTACGATGCGGGGGTTCTCATCCCCTGCAA |
| >CRISPR_II_metagenome_YMACF06TF-VT-6 |
| AGTGCAGACGATTAAACTCGTCACACACTGCCATGAGTT |
| >CRISPR_II_metagenome_YMACF06TF-VT-7 |
| AATGAAATGAACTGTGTACAGCTCGATGAAGATACCTTC |
| >CRISPR_II_metagenome_YMACF06TF-VT-8 |
| ATGGTGAGTATTGTCAAAGACTAACTTTCATCAAAGGC |
| >CRISPR_II_metagenome_YMACF06TF-VT-9 |
| TACCATCTGTTCCTACTGCTGGTGGTGGAATTGCAT |
| >CRISPR_II_metagenome_YMBDR75TR-VT-1 |
| GGTTAACCCAACTGGGGTGAAGTTGTAGAGGATAACTTTG |
| >CRISPR_II_metagenome_YMBDR75TR-VT-2 |
| GTCAAAACAGGTTCCATAACAACCTCCCTTTACGGAGT |
| >CRISPR_II_metagenome_YMBDR75TR-VT-3 |
| ACTCTCTTCGAGGACCAACCAGCGATTCTTATAAGGAATC |
| >CRISPR_II_metagenome_YMBDR75TR-VT-4 |
| TCATAACTAACCTCCAAAGGAGTAAAGTTTTCAAAAATAAAA |
| >CRISPR_II_metagenome_YMBDR75TR-VT-5 |
| ACAGTTTTCTTCTACAGAGGAAACTGGGTGATCTTCCAAGAC |
| >CRISPR_II_metagenome_YMBDR75TR-VT-6 |
| TTTTTCAGTGAAGTTCGGGACGTATTGGTAGCGAA |
| >CRISPR_II_metagenome_YMBDR75TR-VT-7 |
| TGGAAGAAAAGCGCGGGAACACCGTGGTCCAATACCAATA |
| >CRISPR_II_metagenome_YMBDR75TR-VT-8 |
| CAGAGGAAAACAAAGCTTATATGGATCAAAAAAAAGAA |
| >CRISPR_II_metagenome_YMBDR75TR-VT-9 |
| AAGAAAGTTAATTCAAATTATTCCTCCCGCTGAGCC |
| >CRISPR_II_metagenome_YMBDR75TR-VT-10 |
| CCTCAAAGAACAGCGGAAGCAGATGGAAGCCGCTCTGAA |
| >CRISPR_II_metagenome_YMBDT21TF-VT-1 |
| TTCAGGGGATCCAGATCATCCCTGGAACAAAGCAGGGGGAGTTTCCAAA |
| >CRISPR_II_metagenome_YMBDT21TF-VT-2 |
| TCGGAAGGAGAAGCCTTGTCGGCAGGCTTCTCCTC |
| >CRISPR_II_metagenome_YMBDT21TF-VT-3 |
| GTACTTGGCTAAGGGCAGAGGCTCTGCGGAAACGCAA |
| >CRISPR_II_metagenome_YMBDT21TF-VT-4 |
| TTAATCACAGTCAAACAACAGAATAAGTCCATCTGGCATTTCTT |
| >CRISPR_II_metagenome_YMBDT21TF-VT-5 |
| TTACACTAATCGCACCTTCTGGACCCAACACTTATTTC |
| >CRISPR_II_metagenome_YMBDT21TF-VT-6 |
| TTGAATGAAGTTCCCTTTTGTAAAAGGGTTGTAGCA |
| >CRISPR_II_metagenome_YMBDT21TF-VT-7 |
| CGCTCTGCCCTTAGGATTTGCTCTTTTGTGTACTTCATAACT |
| >CRISPR_II_metagenome_YMBDT21TF-VT-8 |
| AGTAAAAATCTCTACCTACGTCACTGCTACATGAAGTA |
| >CRISPR_II_metagenome_YMBDT21TF-VT-9 |
| GGTATAGATGTATAGCCTAATAGGGATAAAACTGTAT |
| >CRISPR_II_metagenome_YMBDT21TF-VT-10 |
| CAGAATGTCCACCAAGCGAGTTGGTTCCAATTAACA |
| >CRISPR_II_metagenome_YMBDT21TF-VT-11 |
| TGAACTAACCTCCCCCAAGACACCAGGTTTTCAT |
| >CRISPR_II_metagenome_YMIAI61TR-VT-1 |
| TAGAAGTTAACCCCGAGGAGAATAAAAACATTCTCTCTA |
| >CRISPR_II_metagenome_YMIAI61TR-VT-2 |
| CTCCAACTAGGCAGTACTAAACAAACTAACCTACTCTAAAAA |
| >CRISPR_II_metagenome_YMIAI61TR-VT-3 |
| ACCAGTCGAGCTGGTCTTGTGTAAGACCAGTATATTGGGT |
| >CRISPR_II_metagenome_YMIAI61TR-VT-4 |
| ACCAAACTTAGACGCGATTTCAACTAGTCGATCCC |
| >CRISPR_II_metagenome_YMIAI61TR-VT-5 |
| TGGTAAATTTGGGAAGCTAACGCATGAGCGCGGCGC |
| >CRISPR_II_metagenome_YMIAI61TR-VT-6 |
| ACTTATCAGGATCGAAATATTGTGGAGCGTACTTTACA |
| >CRISPR_II_metagenome_YMIAI61TR-VT-7 |
| TGGATTGGCTTGTTAGGCCTAATTTTAGGCCCTTTGGCAA |
| >CRISPR_II_metagenome_YMIAI61TR-VT-8 |
| ATCCATATAGACTCCTCCCTTGTGTGGGATGAAAGAA |
| >CRISPR_II_metagenome_YMIAI61TR-VT-9 |
| TGGAGGTTTATCTACTGCCTAAAGTTGAGCTGTACACT |
| >CRISPR_II_metagenome_YMIAI61TR-VT-10 |
| GAGAAAGACTTTAAGTAATCTGAGAGCTTGCATGAGGG |
| >CRISPR_II_metagenome_YMIAS32TR-VT-1 |
| TTCTACGTTCCCGAGGCTGAAGATTAAGCAGTAACCTCCC |
| >CRISPR_II_metagenome_YMIAS32TR-VT-2 |
| CCTTGATCAAGTATGCGTACTTGGTAACTCGGTATGAG |
| >CRISPR_II_metagenome_YMIAS32TR-VT-3 |
| TTCTACGTTCCCGAGGCTGAAGATTAAGCAGTAACCTCCC |
| >CRISPR_II_metagenome_YMIAS32TR-VT-4 |
| GATGTGGACATTAAGGCCGCGTCGGCACTTATCGA |
| >CRISPR_II_metagenome_YMIAS32TR-VT-5 |
| ATCGTTTGGGGTCGCGGGTACTGGAAGATTAGCTGGT |
| >CRISPR_II_metagenome_YMIAS32TR-VT-6 |
| ATGAGGAGCTTAAAGAGCTGAAAGCTAAACTGGGGATCTA |
| >CRISPR_II_metagenome_YMIAS32TR-VT-7 |
| CTGGGTCCGACTGACTCGTCGGTGCCCGTACCTCTA |
| >CRISPR_II_metagenome_YMIAS32TR-VT-8 |
| CGCCAGCAGACGGAGGAGCTAATCAGAAGGTTGGAGT |
| >CRISPR_II_metagenome_YMIAS32TR-VT-9 |
| ACCAATCTCTAAACCTCCCCCAACCAACAGCACCCAGGTT |
| >CRISPR_II_metagenome_YMIAS32TR-VT-10 |
| GGTACAATCGCTGACTTGTTAGACCTTGCAAGTCGA |
| >CRISPR_II_metagenome_YMJAO09TR-VT-1 |
| CTGCGGAAGCCCTGCGTAAGCAGGTGGAAGCAATGCGCCAA |
| >CRISPR_II_metagenome_YMJAO09TR-VT-2 |
| ATTCGAGCGGCAAAAGCCGCGTACCGGGCTGCCAT |
| >CRISPR_II_metagenome_YMJAO09TR-VT-3 |
| ACACACCCAGGTGAAATACCTGGGTGTTCTTTATCCCTAGTGAAGGGA |
| >CRISPR_II_metagenome_YMJAO09TR-VT-4 |
| ATGCTGCTTGACCTCAAGCTCAGATATAGCATCAGCATGCGA |
| >CRISPR_II_metagenome_YMJAO09TR-VT-5 |
| AAGGGAGGTAGTATGGTGAAATATACCAAAGAGCAAATT |
| >CRISPR_II_metagenome_YMJAO09TR-VT-6 |
| AGGTCCTTCAGCCGAATCCCCTCGATCTTTCTTCTAGGG |
| >CRISPR_II_metagenome_YMJAO09TR-VT-7 |
| TTGCGACTACATCAGGGATGTCGCAAAAATCCTGGGCATGGT |
| >CRISPR_II_metagenome_YMJAO09TR-VT-8 |
| TTTTGGTTCCGCAAGTAAGGGGGTGGGGCCCCTTTAA |
| >CRISPR_II_metagenome_YMJAO09TR-VT-9 |
| ACATTTACACAAGGCCAGGTGAACTGGCTACTTCGCAA |
| >CRISPR_II_metagenome_YMJAO09TR-VT-10 |
| GAGTACGTCAAGTCCCTGACTGATGTCCAGCTTGCTGT |
| >CRISPR_II_metagenome_YMJAO09TR-VT-11 |
| TCGAAAATAAAAAGGGGTAAGTCCCACACAAGTTGCTTCCT |
| >CRISPR_II_metagenome_YMJAY30TF-VT-1 |
| AGAAAAAGTCTTTGGTTGTTGTCGTGTAGTTTGGAA |
| >CRISPR_II_metagenome_YMJAY30TF-VT-2 |
| TTTGATCTCCATAGTGTACCTCCCTTCTTCAGGGATAAA |
| >CRISPR_II_metagenome_YMJAY30TF-VT-3 |
| TAAGAATGTACAACAAGTTAGTGGAACACATCAACTA |
| >CRISPR_II_metagenome_YMJAY30TF-VT-4 |
| CGCTCTGCATAGTTACGGATGACCCAGGGACCCAACA |
| >CRISPR_II_metagenome_YMJAY30TF-VT-5 |
| AAGCTGGATCCTAACCAGCTTCAGCAGCTAAACCAACTTAA |
| >CRISPR_II_metagenome_YMJAY30TF-VT-6 |
| GGGAAATTAGAGGCCTCTCAGACAACATTTGAGAGG |
| >CRISPR_II_metagenome_YMJAY30TF-VT-7 |
| TTGCACCAGTCAATCTACAATCGGATTGGCTACGTGCTAT |
| >CRISPR_II_metagenome_YMJAY30TF-VT-8 |
| CTCCCGAGCTCGGTGTACCAGGTCACGGTTTTCAAGCAGTTT |
| >CRISPR_II_metagenome_YMJAY30TF-VT-9 |
| GCCTTCCTCTGCGCTTAAGTAATCCAAAAGTTTGTAC |
| >CRISPR_II_metagenome_YMJAY30TF-VT-10 |
| GTGCAGTTCCAGAGCGGAGGTTGGCTGGCAGGATAAC |
| >CRISPR_II_metagenome_YMJAY30TR-VT-1 |
| ACTCTGGCTCAGGCAGCCTCTAGAAGGGACTTTAC |
| >CRISPR_II_metagenome_YMJAY30TR-VT-2 |
| AGGCCAAGAGACGCAGGAAAACGCGGGTCTTCATACAGC |
| >CRISPR_II_metagenome_YMJAY30TR-VT-3 |
| GCTGTTCCTCAAAAACATCCCGGTCCTCTGGTTAAACGTGCCG |
| >CRISPR_II_metagenome_YMJAY30TR-VT-4 |
| ATGTTGTGGTGGGCTAAGTCCTGTGCAATAAACTAAA |
| >CRISPR_II_metagenome_YMJAY30TR-VT-5 |
| AAAGCTCATATTCTGTGCATAGTACAGGTGTTGGGTAGTTTACG |
| >CRISPR_II_metagenome_YMJAY30TR-VT-6 |
| CTTTGGCAGGAAACCCCAGCAGTGCGTCTCTTACACTG |
| >CRISPR_II_metagenome_YMJAY30TR-VT-7 |
| CAAACTGACCGACGAAATCATCCGCGAGGCATTGCG |
| >CRISPR_II_metagenome_YMJAY30TR-VT-8 |
| CCACTCTTTATTGGAGTGCGGGTTAACCCAGTAGGA |
| >CRISPR_II_metagenome_YMJAY30TR-VT-9 |
| TCTTGAAACTGTTGAGTTATGTAGGAGTATTGCAGATC |
| >CRISPR_II_metagenome_YMJAY30TR-VT-10 |
| TTCAGTTTGCTGATCTGGTTGGAATCCAGCTTAGTGCA |
| >CRISPR_II_metagenome_YMAAW47TF-VT-1 |
| CTTAATACTCTCGTCATCCATTTGTTCAGTGAAGG |
| >CRISPR_II_metagenome_YMAAW47TF-VT-2 |
| AGCCAAGTCTTGGCCAGGCTCACTTGAGCTTGATTCA |
| >CRISPR_II_metagenome_YMAAW47TF-VT-3 |
| TCCGGGGTTTTGTTGTCGATGACCGTGGACGCCGTGTCG |
| >CRISPR_II_metagenome_YMABW81TF-VT-1 |
| GGTGTTACAACCATGTAGTAACCGTCGACCCCACCCAACTT |
| >CRISPR_II_metagenome_YMABW81TF-VT-2 |
| TCCTGCTCTTTGGTCAGTTTCCGCGGCCCAGTCACTCCCACGT |
| >CRISPR_II_metagenome_YMABW81TF-VT-3 |
| ACCACACATGTCAAGACGAACAACCGCCATACAAACCTCCTG |
| >CRISPR_II_metagenome_YMABY66TF-VT-1 |
| TTGGAAACGACAGGAACCAGGAAATATAACAGATCCTTCGGATT |
| >CRISPR_II_metagenome_YMABY66TF-VT-2 |
| TTGGAAACACTCGGGAGGGCAGTTAACGATGGGGATCTCAATGGGGGAG |
| >CRISPR_II_metagenome_YMABY66TF-VT-3 |
| ATTAGAAGGACACCGAAAGGGGTCCATT |
| >CRISPR_II_metagenome_YMABY66TF-VT-4 |
| TTGGAAACTAGTATTCATACCACTCATCAGGGTGGTGAAAGACAATC |
| >CRISPR_II_metagenome_YMABY66TF-VT-5 |
| TTGGAAACGCCCAGGGAGTTTAAGAACTCCCGGGCTTGCTTTAGTCACGCGTCCTCAA |
| >CRISPR_II_metagenome_YMACD96TF-VT-1 |
| ACAATTCGAGAAGCTCCTCTAGGCCCATCCCGAAGTGGTCTTCGGCA |
| >CRISPR_II_metagenome_YMACD96TF-VT-2 |
| ACCGCAGGGCCACCGCTTCTTCTTGGCGCAGGTAGTAGCTC |
| >CRISPR_II_metagenome_YMACD96TF-VT-3 |
| ACTGCCACAGCCAGGCAGCCCCTTTGGGGCCGAAGTTAA |
| >CRISPR_II_metagenome_YMACD96TF-VT-4 |
| GCAAACGAAAGCCCCCGCCAGCGCGGGGGCT |
| >CRISPR_II_metagenome_YMBBC81TR-VT-1 |
| AACGATATCCCCCTGACCCCCTTACCCCCGCTTTCCCATCGT |
| >CRISPR_II_metagenome_YMBBC81TR-VT-2 |
| AACTTCTCCAGCCGCTTATTCTGGAACCACGTAACTCCATT |
| >CRISPR_II_metagenome_YMBBC81TR-VT-3 |
| AACGCCCAACAGCTCTTCCTTGATAAATCCTTCTCTAATAG |
| >CRISPR_II_metagenome_CYNA391TR-VT-1 |
| CTTTCGTCCACGCTTCGCGTATGACGTTTTCGGGCACCTT |
| >CRISPR_II_metagenome_CYNA391TR-VT-2 |
| TATTAGAGATACGGGCAGCGCTTAGTCATGCGCTTCCA |
| >CRISPR_II_metagenome_CYNA391TR-VT-3 |
| CACTAAGTATCCCATCTGGTGGTAGTCAATCCAGTCTGCAT |
| >CRISPR_II_metagenome_CYNA391TR-VT-4 |
| CTCCCTTTTCAGGGGATAAACAACACCCAGGATTAT |
| >CRISPR_II_metagenome_CYNA391TR-VT-5 |
| TTTTACGACGAAGTATCACTAGTTCGTCACTACCCCG |
| >CRISPR_II_metagenome_CYNA391TR-VT-6 |
| CCCAAGGAGGGCAGGGGAGCCCCTCGAAGATTTCATGCT |
| >CRISPR_II_metagenome_CYNA391TR-VT-7 |
| CTCGCACACCTGGATTTGCGAGGTCTTAATCTTGAC |
| >CRISPR_II_metagenome_CYNA391TR-VT-8 |
| GTCCGTAAACAAGCCCCCCTCTCGGGCGGGCATTTTCGA |
| >CRISPR_II_metagenome_CYNA391TR-VT-9 |
| CCGATGGGATTTCGGGGCAGTCCAGATGCTGCTCCCATGAT |
| >CRISPR_II_metagenome_CYNAA15TR-VT-1 |
| TTGTAGAGGTATGTAGCTACCTCTGTTCCCCCAAAGAACCT |
| >CRISPR_II_metagenome_CYNAA15TR-VT-2 |
| TTCTCTTCGTGAAGTGAGCAGATTTCGAGGTACTCAT |
| >CRISPR_II_metagenome_CYNAA15TR-VT-3 |
| CAATCTGATCCCACAAATCATTAAAGTGCTCTTCTAGACGAGG |
| >CRISPR_II_metagenome_CYNAA15TR-VT-4 |
| AAATAAAAAGGCTACCACTAGGGTAGCCTCTGAGGAAAGTG |
| >CRISPR_II_metagenome_CYNAM11TR-VT-1 |
| AGGGTACCTAGTTAGTACGAAGCAGTGATGCTCAAGTAGGT |
| >CRISPR_II_metagenome_CYNAM11TR-VT-2 |
| GTTTCTCAATGTTTGAGAACGATTAACTATAAAAATGCACTT |
| >CRISPR_II_metagenome_CYNAM11TR-VT-3 |
| TAATTGCAGCGTTTAAGCCTCTACACAAAAAACAATCCA |
| >CRISPR_II_metagenome_CYNAM11TR-VT-4 |
| TGGAGGTAATCATGTTGAACAATCGCCAGAAGCGGCGTGCTC |
| >CRISPR_II_metagenome_CYNAM11TR-VT-5 |
| AGGCTCTGCGAACCCGTAAGGCGGAGTGCCAGCCGAGCGAGT |
| >CRISPR_II_metagenome_CYNAM11TR-VT-6 |
| AGTCTATACCAGGCTAATCTAGCCGTCAATACCATATGCGC |
| >CRISPR_II_metagenome_CYNAM11TR-VT-7 |
| AAGCTAGGGGGAGTGCTTTACGCCTACCCCAACAA |
| >CRISPR_II_metagenome_CYNAM11TR-VT-8 |
| GAGAAAATCCGAGTGTACACCGACTGAGTTAGTCCCCATCAA |
| >CRISPR_II_metagenome_CYOAB48TF-VT-1 |
| TCCAGCATTTTTTTACAGAGGAGTATTAAAACAATTTT |
| >CRISPR_II_metagenome_CYOAB48TF-VT-2 |
| ACTTGTGACCGACCGCTTCAGGTTTGTGTAGGTTT |
| >CRISPR_II_metagenome_CYOAB48TF-VT-3 |
| ACCAACCGACCGATGCTCGCGGCTACTTCACTCGG |
| >CRISPR_II_metagenome_CYOAB48TF-VT-4 |
| TTTGGCAACAGCGTAGGAATTTGCTCTGGCTTAGCCTCAGTCA |
| >CRISPR_II_metagenome_CYOAB48TF-VT-5 |
| AATCTTTGCTAAGCCATTGCTTAACCAACTGGATTTGGGC |
| >CRISPR_II_metagenome_CYOAB48TF-VT-6 |
| GGAAGGTCTTGTGGAATAACTCCACTATCATGTAACGACC |
| >CRISPR_II_metagenome_CYOAB48TF-VT-7 |
| AGTCGAAGAGTTAGCACAACCGATAATGGCTTATCTGCCTTT |
| >CRISPR_II_metagenome_CYOAB48TF-VT-8 |
| TAGACATGCCGTTGCCCAGTCGGGCTGAGGGATGCCG |
| >CRISPR_II_metagenome_CYOAB48TF-VT-9 |
| CGCAGCTAAGATTCGGAGGGCATACCACAAGGACTT |
| >CRISPR_II_metagenome_CYOAB48TR-VT-1 |
| TAATTGCAGCGTTTAAGCCTCTACACAAAAAACAATCCA |
| >CRISPR_II_metagenome_CYOAB48TR-VT-2 |
| AACCTATATCCCGTGGTATCACGCGGCACGGCTACTAGACTT |
| >CRISPR_II_metagenome_CYOAB48TR-VT-3 |
| AACCTATATCCCGTGGTATCACGCGGCACGGCTACTAGACTT |
| >CRISPR_II_metagenome_CYOAB48TR-VT-4 |
| AGAACTATGGCTATGCTAACTGGTCGTCAGAAGCGTCGT |
| >CRISPR_II_metagenome_CYOAB48TR-VT-5 |
| TGCATACATGGCCGAAGAGGCCTATGCGGCTACGTTCGG |
| >CRISPR_II_metagenome_CYOAB48TR-VT-6 |
| AAGATGGGTAATTAGCTGGTAGTCAGCGATCCCCA |
| >CRISPR_II_metagenome_CYOAB48TR-VT-7 |
| TGCGACGAGTTCGAATCGCTGAGTAGAGCCTTGACCAT |
| >CRISPR_II_metagenome_CYOAB48TR-VT-8 |
| TGCGACGAGTTCGAATCGCTGAGTAGAGCCTTGACCAT |
| >CRISPR_II_metagenome_CYOAB48TR-VT-9 |
| TGCGACGAGTTCGAATCGCTGAGTAGAGCCTTGACCAT |
| >CRISPR_II_metagenome_CYOAB48TR-VT-10 |
| CGTCATGAGAACGGACGATGGGTCTCTTGCCTGAATAAAGAC |
| >CRISPR_II_metagenome_CYOAE25TF-VT-1 |
| TGGCCGTCCTGGAAGGCTTCGCCCTCCAAAGCCGGAAAGATT |
| >CRISPR_II_metagenome_CYOAE25TF-VT-2 |
| CTTTCTTGTACTCCTCGAAATCACTAAAAGTCAAAC |
| >CRISPR_II_metagenome_CYOAE25TF-VT-3 |
| ACATCCCTACGCACCCGCTGGTTGAACCGTTTGCGGGCCCGCTC |
| >CRISPR_II_metagenome_CYOAE25TF-VT-4 |
| TTACTGCCATAAATGTAGCGAATCCCCCTGTTAGT |
| >CRISPR_II_metagenome_CYOAE25TF-VT-5 |
| GCTCGGTACTGTAGCATTGAGTGGAGCTTCTCCCAGGTT |
| >CRISPR_II_metagenome_CYOAE25TF-VT-6 |
| CGTCAGACAACTGACCGGTGCCAGTATGGTACAGTTAATTGGT |
| >CRISPR_II_metagenome_CYOAE25TF-VT-7 |
| AACACCTCGATCAGGGCGCTTTTGGCCTTGACATCCGG |
| >CRISPR_II_metagenome_CYOAE25TF-VT-8 |
| TTGCTCGAGATGAGCCCCGGGCAGGGCCCACCGTTTGCA |
| >CRISPR_II_metagenome_CYOAE25TR-VT-1 |
| CAGCAACGCCAGGAAGAGGCGACTCGTCGTAAGACCGTTGC |
| >CRISPR_II_metagenome_CYOAE25TR-VT-2 |
| AGCCTTTAATGGGGTTAGTCAGTTATGATCCAACCTTCAC |
| >CRISPR_II_metagenome_CYOAE25TR-VT-3 |
| AAGATGAGTATCTTTCCGACTGCATTGTAGATCCAGA |
| >CRISPR_II_metagenome_CYOAE25TR-VT-4 |
| ACGAACAGTATTCGCGACGCATACGAAGTCCTGAA |
| >CRISPR_II_metagenome_CYOAE25TR-VT-5 |
| GTGAAATACCACTACAGTCCTTACCACAAAGGCAACTT |
| >CRISPR_II_metagenome_CYOAE25TR-VT-6 |
| TTAGAGGGAGAGAATGAATTACCAAAACCTAATCTTACTT |
| >CRISPR_II_metagenome_CYOAE25TR-VT-7 |
| GATTCGCGTTTCAACGGCCTGATCCCAGTGACATCGAA |
| >CRISPR_II_metagenome_CYOAX80TF-VT-1 |
| GCGATCCTGGTTGTATGCTACGGCGAACACGTCGTAGCAT |
| >CRISPR_II_metagenome_CYOAX80TF-VT-2 |
| AAAAAGGGGACGCCTCGGACAAGATTTGAACTTGTGAC |
| >CRISPR_II_metagenome_CYOAX80TF-VT-3 |
| AGGACTCCAGTTTAACTTTGCAGACTATAGTGAATATCT |
| >CRISPR_II_metagenome_CYOAX80TF-VT-4 |
| CAGGTTCTTCGGTCCCAGCGCTGCAAGTATGTCCGTGA |
| >CRISPR_II_metagenome_CYOAX80TF-VT-5 |
| CAAATGAAGTCCGCCTGTATATTTACAACCCCTTTTGTAG |
| >CRISPR_II_metagenome_CYOAX80TF-VT-6 |
| ACTTACCCCCGTTTTCAACATGAAGACGACAAAGTT |
| >CRISPR_II_metagenome_CYOAX80TF-VT-7 |
| TAATTAACTGGGAATCGATTCTAGAAACAAATTCCTC |
| >CRISPR_II_metagenome_CYOAX80TF-VT-8 |
| CGCCCAGGATCTGGCGAGCAGCTGCAACCACTCGGAATCGCT |
| >CRISPR_II_metagenome_CYOAX80TF-VT-9 |
| TCTATACACTGGTAGTCAATGGAGGCATCGATGAGCGACTT |
| >CRISPR_II_metagenome_CYOAX80TF-VT-10 |
| GTGGAGGTATCCCTCATACAAACTCTTGGATTACTTAAG |
| >CRISPR_II_metagenome_CYOAX80TR-VT-1 |
| AGTAGGTCGTTATCCAGGTAGTTCCTTCTCTTGTCAC |
| >CRISPR_II_metagenome_CYOAX80TR-VT-2 |
| TTTCAACCCCATTGGTTTGGACACCAATAGGTTTACTGGG |
| >CRISPR_II_metagenome_CYOAX80TR-VT-3 |
| GGTTGGTACACCTCGGCTAGGGGCTTGTCAGCCTCCTT |
| >CRISPR_II_metagenome_CYOAX80TR-VT-4 |
| GCTGTATGCGTAGCTTCGTCATACTACGCAGGCATTTGATT |
| >CRISPR_II_metagenome_CYOAX80TR-VT-5 |
| AAGCTACGCATACAGCGTGGTGAATATTACGAGGCATTGAA |
| >CRISPR_II_metagenome_CYOB470TF-VT-1 |
| CCCGCCTTATTGGCGGGGGATTTTTTGTGGCTATAGG |
| >CRISPR_II_metagenome_CYOB470TF-VT-2 |
| TCGCTTTGTCCAAAGCGCACTCAATACACGTTTCGCA |
| >CRISPR_II_metagenome_CYOB470TF-VT-3 |
| ATCTATTAGTCGTGCTGTATTGCTATCTGCATCTTCTT |
| >CRISPR_II_metagenome_CYOB470TF-VT-4 |
| AGCAAATTCTTCGTTCCTCTCGGTGCGAGTATATTCGCG |
| >CRISPR_II_metagenome_CYOB470TF-VT-5 |
| TACCACCTCGGGAGCGGACTCATCTTTGGTCTTTGCAC |
| >CRISPR_II_metagenome_CYOB470TF-VT-6 |
| TTTACCCAGACATAGAATTTGGAAGTTGTACATAGCTGCT |
| >CRISPR_II_metagenome_CYOB470TF-VT-7 |
| GATAAAGTGGAATACTTCAACGATTGTTTCCTCCGGGAG |
| >CRISPR_II_metagenome_CYOB470TF-VT-8 |
| TGGACCAAATACAGTGGTTTGCCAGAACCATAGTCTACC |
| >CRISPR_II_metagenome_CYOB470TR-VT-1 |
| ACAACTCCGGGAGCTGACCGTATGGTCAGTGAAAG |
| >CRISPR_II_metagenome_CYOB470TR-VT-2 |
| ATGGCCAACTCCCTCAGGGAAGGTTTGGCTGTTCCTCAA |
| >CRISPR_II_metagenome_CYOB470TR-VT-3 |
| TGTCGGACGGTTCCGGGTAACCGGGGTCACCAACGGGT |
| >CRISPR_II_metagenome_CYOB470TR-VT-4 |
| ACCAACGAGTGTGATCATTTTGCACCTCCCTTATGTGGGTAG |
| >CRISPR_II_metagenome_CYOB470TR-VT-5 |
| TACTTGGCGTCTAATTCAGTAAGGTTGTTCGAAAGAACCAG |
| >CRISPR_II_metagenome_CYOB470TR-VT-6 |
| GAGAACACAGTCCGTGCCTAGCACCTTCTCAATGAT |
| >CRISPR_II_metagenome_CYOB470TR-VT-7 |
| TTTATTTTAGGCAGACCTTGTTGTTCACTGCCTACAGG |
| >CRISPR_II_metagenome_CYOB470TR-VT-8 |
| TTACTGAGTTAAACAACAATTTCATGTTCAAACTGGTAAATCTA |
| >CRISPR_II_metagenome_CYOB470TR-VT-9 |
| AGTAGTTATAATCATCATTGTCATCATTGTTATTAAC |
| >CRISPR_II_metagenome_CYOB470TR-VT-10 |
| GTTCTAGATCATCAAATCTTTTGGAGAAGAACTTAATA |
| >CRISPR_II_metagenome_CYOB470TR-VT-11 |
| TTGAAGTTGTTGATGCAGATTGGAGGTTGACTA |
| >CRISPR_II_metagenome_CYOBC31TR-VT-1 |
| TAAGCAAAGATCGACTCTTCATTGAATGGGAAAAGTGGGCTAAA |
| >CRISPR_II_metagenome_CYOBC31TR-VT-2 |
| TTATAACATCCCTGGCTCCAAGCCTGTAATCGAACC |
| >CRISPR_II_metagenome_CYOBC31TR-VT-3 |
| GGTAGATTGCGGCGACTTTTCCTACCAGCAATTCATGGTGGT |
| >CRISPR_II_metagenome_CYOBC31TR-VT-4 |
| CAAATCTGGCTCAGCGGGAGGAATAAGTTGAATTAA |
| >CRISPR_II_metagenome_CYOBC31TR-VT-5 |
| TGGAGGAGGCTCTGCCAGTTGGGTCTGGGATAAACGT |
| >CRISPR_II_metagenome_CYOBC31TR-VT-6 |
| TACATAAGGTACATCCCTTCATGGGCAGTAGCGGGTAGCACGATCTAGT |
| >CRISPR_II_metagenome_CYOBC31TR-VT-7 |
| AGCATGGTGAAGTAATTGCAGTCTGTTATTACGCAGACTGGA |
| >CRISPR_II_metagenome_CYOBC31TR-VT-8 |
| GTTACTGTTATTCCCTGCCTTAGGAGGTTAACTATGACTACCA |
| >CRISPR_II_metagenome_CYOBC31TR-VT-9 |
| TTGTCTCCCATCCCGAACCCCACGAAGAAAATCAAAAACAATA |
| >CRISPR_II_metagenome_CYOBC31TR-VT-10 |
| AGAAGAGTGGTACCAGGATGCATTCGAGCCGT |
| >CRISPR_II_metagenome_CYOBD48TR-VT-1 |
| CATAAGCAGAACACACCCGTCGATGCCGAGGTTGTCGG |
| >CRISPR_II_metagenome_CYOBD48TR-VT-2 |
| TATTAGCTCAGTGGGGTCTGCAGCTCCAATTGATTGCGGAC |
| >CRISPR_II_metagenome_CYOBD48TR-VT-3 |
| TCGATGAGCCAAAGCCCATCTCCCACAGGTATTGCAGT |
| >CRISPR_II_metagenome_CYOBD48TR-VT-4 |
| TTGTGCTTCCCATGCTTTAAGCGTTCCTTTAGTTGTC |
| >CRISPR_II_metagenome_CYOBD48TR-VT-5 |
| TTGGGGTACGAGGTAACCCAAGAGTCGGACTACCTATGTACG |
| >CRISPR_II_metagenome_CYOBD48TR-VT-6 |
| AAACCACCCCTTCCAAAAGGGTGGTTTTTCTATCTGCTTAAGG |
| >CRISPR_II_metagenome_CYOBD48TR-VT-7 |
| GCGAATCAAGTTGTTGGGTCCTGTTCTCGCTTACCC |
| >CRISPR_II_metagenome_CYOBD48TR-VT-8 |
| CTCTTTTGTCAGCATCTTTGTTACTCCACCATTCTAGTTT |
| >CRISPR_II_metagenome_CYOBD48TR-VT-9 |
| TCACATGAGCCCGGTAAATGTTTAGTTCAGTTTGAAGGGAA |
| >CRISPR_II_metagenome_CYOBD48TR-VT-10 |
| ATTCTGGAGGTAGAAGCTCAACCTACTGCACTGAGAGC |
| >CRISPR_II_metagenome_CYOC968TR-VT-1 |
| ATCAAGATGCTTTGGTTCTGGCAACTCCACTACAGTG |
| >CRISPR_II_metagenome_CYOC968TR-VT-2 |
| AACACCAGGGATCCAGCCACAATTAGGCTGCAGCTTGTCCC |
| >CRISPR_II_metagenome_CYOC968TR-VT-3 |
| GCCTGGTCCACTCCACTGGTGGGACAAGAAGGCCCCCTCCACCTT |
| >CRISPR_II_metagenome_CYOC968TR-VT-4 |
| AGCGACCGACATGCGAATGCTCTTCGCATTCACTTCTTTC |
| >CRISPR_II_metagenome_CYOC968TR-VT-5 |
| ATTCCCTGCCAGTTGGAGGTTGGTTATGGTTATCAA |
| >CRISPR_II_metagenome_CYOC968TR-VT-6 |
| GCTGACCCTTGCTGGCGGGGCCGACCACACCACCACGTCCATCCAG |
| >CRISPR_II_metagenome_CYOC968TR-VT-7 |
| TTCTAGGGCAGCTAGCCCGGACGGACCTGTTCCACCAAT |
| >CRISPR_II_metagenome_CYOC968TR-VT-8 |
| AGGCTGTTAAACGCAGTCGGACTGGGAATTCTCGCTA |
| >CRISPR_II_metagenome_CYOC968TR-VT-9 |
| GGGGAGTTCATCAAACGCATACCGAAGGGGATTCAGTTTA |
| >CRISPR_II_metagenome_CYOC968TR-VT-10 |
| ATGACCAATCAGTCACTGTAGTTGAGATTGAGCTT |
| >CRISPR_II_metagenome_CYOCE57TF-VT-1 |
| TATTTGCGGATGCCTAATGTGTACAAGATCTCGTTAG |
| >CRISPR_II_metagenome_CYOCE57TF-VT-2 |
| ATTTTCCGATCTTTTGTCGACCACACCTCCAGGTTGCGCAGT |
| >CRISPR_II_metagenome_CYOCE57TF-VT-3 |
| TCCCTGGAAAACGGGCGGGAAATTGGTTAGGGTAACCGAAA |
| >CRISPR_II_metagenome_CYOCE57TF-VT-4 |
| GAACGGGACGGTTGTCCACGTTCTTCTTGGCCTCTACCGA |
| >CRISPR_II_metagenome_CYOCE57TF-VT-5 |
| TCTGAATAACCTCTTCCCGGCTGCCATCGCGCCCAATCTT |
| >CRISPR_II_metagenome_CYOCE57TF-VT-6 |
| GCGGCTCAGGTTCTTGCGAACCTTTTTGTTGAACCGT |
| >CRISPR_II_metagenome_CYOCE57TF-VT-7 |
| CTTTTCGCTTTTGCCCGAATGACCTCCTCAATTTGGCC |
| >CRISPR_II_metagenome_CYOCJ70TF-VT-1 |
| GCCGGGTTGGGCAGTGATGGGTGAGCTCCTATAAAA |
| >CRISPR_II_metagenome_CYOCJ70TF-VT-2 |
| TTCTCGAAGAGGGGTGGGATGACTTCGTAGTCCAATA |
| >CRISPR_II_metagenome_CYOCJ70TF-VT-3 |
| GCGGCAGCGGGCTGAACAACAAGCCCGTGTAGAACGA |
| >CRISPR_II_metagenome_CYOCJ70TF-VT-4 |
| TCATCTCTTCCTCCTCTCTCGGCAGTGCTAAACAA |
| >CRISPR_II_metagenome_CYOCJ70TF-VT-5 |
| TGCCCGATTACGAATGGGCCAATAGCCGCTAATAACTAGT |
| >CRISPR_II_metagenome_CYOCJ70TF-VT-6 |
| ATTAAATTCCTGCGTCAATTGGCCAAAACTTTCGGTT |
| >CRISPR_II_metagenome_CYOCJ70TF-VT-7 |
| TTGACTGTGAGAGAGAGATACCTAAGGAAATTTGGC |
| >CRISPR_II_metagenome_CYOCJ70TF-VT-8 |
| TTGCTTAGTTTCTGGACCACCGAAGTGGGTCCGGCTATCGC |
| >CRISPR_II_metagenome_CYOCJ70TF-VT-9 |
| TCCGTGTGCCCGATTTTGAGTGGGCCAATAGCTAATATAA |
| >CRISPR_II_metagenome_CYOCJ70TR-VT-1 |
| AGCAGCTAATGCTACTACTGGAGATGCTTGAGGTAGT |
| >CRISPR_II_metagenome_CYOCJ70TR-VT-2 |
| TACTTTAAACTGACATTTCATCTTCCCCTTCCTCCT |
| >CRISPR_II_metagenome_CYOCJ70TR-VT-3 |
| AGCCCAATATACTGGTCTTACACAGGACCAGCTCGATTGGC |
| >CRISPR_II_metagenome_CYOCJ70TR-VT-4 |
| CCCAATTCTCCAAAATATTTGGGATGAGGTTGAGTATGTC |
| >CRISPR_II_metagenome_CYOCJ70TR-VT-5 |
| CCACAGATTCAGGGTTGGTCCAGAGCCCCAAACCTGGGAGTTA |
| >CRISPR_II_metagenome_CYOCJ70TR-VT-6 |
| CGGTTCAGTCTTTCACCTGCCTTTAGGAGGAACTATGCTTAT |
| >CRISPR_II_metagenome_CYOCJ70TR-VT-7 |
| CTATAGCTCCAGTTTCGAGGTTTGTTAGTCTATAGCCAACACC |
| >CRISPR_II_metagenome_CYOCJ70TR-VT-8 |
| ATACTGGGTGCTGAGGGGTTTAGATCAGCCAATCTTCCTCGT |
| >CRISPR_II_metagenome_CYOCJ70TR-VT-9 |
| TTCTACCTAGATACTCTGCCATTCCGTTTGCATAAGCCA |
| >CRISPR_II_metagenome_CYPAC23TR-VT-1 |
| TGGACCCACCGAAAAGGCCACCCTATGGGCAGAGAT |
| >CRISPR_II_metagenome_CYPAC23TR-VT-2 |
| GGGGGAGGTTGGTTGGGAGGTTGGTGGGAGTTAGACT |
| >CRISPR_II_metagenome_CYPAC23TR-VT-3 |
| GTCTCTTTCTACATCCCAGTAACGATCTAACCTAACCT |
| >CRISPR_II_metagenome_CYPAC23TR-VT-4 |
| CCCTGCTGTCTTGGCCAAGCGGGCCCCAAATCGCTCTG |
| >CRISPR_II_metagenome_CYPAC23TR-VT-5 |
| TTTTCTGCAACACGGTGCATACCTTTTAAGAGCTAGCG |
| >CRISPR_II_metagenome_CYPAC23TR-VT-6 |
| TGATCCAATACCAATACGTTCCAAACTTCAAGGCAAAAGTTCAGT |
| >CRISPR_II_metagenome_CYPAO83TF-VT-1 |
| TTGAGGAGATCCGCGAGAAGTGGGAAGACGGGTTGTC |
| >CRISPR_II_metagenome_CYPAO83TF-VT-2 |
| GTTTGTTGACGGTGAGACCGTCTTTATTTTCAATAATT |
| >CRISPR_II_metagenome_CYPAO83TF-VT-3 |
| TTCAACACCCAGGATTATTTCCTGGGTGTTCTTTATC |
| >CRISPR_II_metagenome_CYPAO83TF-VT-4 |
| CACGGATGAAATAGGGAGTACAGACAATTGAATATTTCCTGGG |
| >CRISPR_II_metagenome_CYPAO83TF-VT-5 |
| TTATCTACAGCAGTAAGCGCAAGTACCTGCGCGACTGT |
| >CRISPR_II_metagenome_CYPAQ10TR-VT-1 |
| ACGAATATCACCTGGTCTTCTTCGAACTGGATTGCCCT |
| >CRISPR_II_metagenome_CYPAQ10TR-VT-2 |
| CGGCGGGGGTACGACACGTCCCACCCCCACTACTTTGTACT |
| >CRISPR_II_metagenome_CYPAQ10TR-VT-3 |
| AAGAAGTACTTGACTAAATCGGTGTCAATACTAACTCTTG |
| >CRISPR_II_metagenome_CYPAQ10TR-VT-4 |
| AAGATGCATTCGGTGTTCTAGCCCAGTGGGGCTTGCA |
| >CRISPR_II_metagenome_CYPAQ10TR-VT-5 |
| TGTATGCGACTATAGTCGTCACACACCGACATGAGTTTGCT |
| >CRISPR_II_metagenome_CYPAQ10TR-VT-6 |
| TGTATGCGACTATAGTCGTCACACACCGACATGAGTTTGCT |
| >CRISPR_II_metagenome_CYPAQ10TR-VT-7 |
| CCACCATTGCAAACCAGGGGACCAAGCAACTTAATCAT |
| >CRISPR_II_metagenome_CYPAQ10TR-VT-8 |
| ATTCTCCGTAGTGAGCGCTGCTCGTACATCAGGGACATTG |
| >CRISPR_II_metagenome_CYPAQ10TR-VT-9 |
| GGCTTGCAGACCTCAATGGGCAGTCGAGAAGTGGTGTAA |
| >CRISPR_II_metagenome_CYPAQ10TR-VT-10 |
| ATCACTACAACGTAACGACCATCGGGTGAACTAAGTTTAAT |
| >CRISPR_II_metagenome_CYPAW81TR-VT-1 |
| GCGCATCTCGGTGACGCTAATGGACATTACCTTCACCGCCGGT |
| >CRISPR_II_metagenome_CYPAW81TR-VT-2 |
| GAATCCGATCTGGTAAGTGTGGGACGTATGTATACTCAATAA |
| >CRISPR_II_metagenome_CYPAW81TR-VT-3 |
| GTGTATACCGAGCCTATTAATAAAGAGGCAGAGCAGGT |
| >CRISPR_II_metagenome_CYPAW81TR-VT-4 |
| TTCTAATTACAAAGGTAGGTGGTACACAGTTAAAA |
| >CRISPR_II_metagenome_CYPAW81TR-VT-5 |
| ACACCCAGGCTCACATACCTGGGTGTCTTTTCTATCCC |
| >CRISPR_II_metagenome_CYPAW81TR-VT-6 |
| CGAACCACCAACTTCGGCTCTTCATGATGTACTTTA |
| >CRISPR_II_metagenome_CYPAW81TR-VT-7 |
| TGTGAGGTTGTCGAGCATCCAGTTGATTTGTTCCT |
| >CRISPR_II_metagenome_CYPAW81TR-VT-8 |
| ACCACCTCTCCCTTTATCAGGGATAAAGAACACCCAGGAT |
| >CRISPR_II_metagenome_CYPAW81TR-VT-9 |
| GCAGTACTTTCGCAGCCTCATACTGCCCTCCCCAAC |
| >CRISPR_II_metagenome_CYPAW81TR-VT-10 |
| CTCATACCGAGTTACCAAGTACGCATACTTGATCAAGTC |
| >CRISPR_II_metagenome_CYPB357TR-VT-1 |
| AGCCCCCGCGCTGGCGGGGGCTTTCGTTTGC |
| >CRISPR_II_metagenome_CYPB357TR-VT-2 |
| ATCATTTGGGGGAAAGGACGCTGGGTGATCTCCTTCTCGT |
| >CRISPR_II_metagenome_CYPB357TR-VT-3 |
| CGACCTACTACGTGGTGGAGGTAGTCAACACCACGACGT |
| >CRISPR_II_metagenome_CYPB357TR-VT-4 |
| TGGCCTGTCCAAGGGTGTCGACACCTGCCCGAGAGGGAGGTGTGT |
| >CRISPR_II_metagenome_CYPB357TR-VT-5 |
| TCTTTCGCCACCTCGAACCCGGAGCCTCCTATGGGGT |
| >CRISPR_II_metagenome_CYPB357TR-VT-6 |
| GGTACAAATGTACATCACGTTGTTTGGTCCGTGGGTTAACTATGT |
| >CRISPR_II_metagenome_CYPB357TR-VT-7 |
| GGCCAAACAAGTCTACGACGCAGGGGGCTTCGTCCAGTGGT |
| >CRISPR_II_metagenome_CYPBH35TR-VT-1 |
| ACATGGTTAACCAATTTTTGCTAGGCCGTTGGCTCCTA |
| >CRISPR_II_metagenome_CYPBH35TR-VT-2 |
| TCTGAGGACATCACGGATGTAGTTATCCACCTGAGAGTTT |
| >CRISPR_II_metagenome_CYPBH35TR-VT-3 |
| CCCAAAAGAGCATCGCGGACGCTACCGCCCACAACGT |
| >CRISPR_II_metagenome_CYPBH35TR-VT-4 |
| ACAAAAACTCCAAAATCTCCAGGCGGTTTAAATCGCCTTT |
| >CRISPR_II_metagenome_CYPBH35TR-VT-5 |
| GTGGTAGCCTTTTTATTTTTTGAAGACTTATACCCTAAG |
| >CRISPR_II_metagenome_CYPBH35TR-VT-6 |
| ACAGAGCGGATAGTTCGGGAGAAAATGCCGGGCTCGT |
| >CRISPR_II_metagenome_CYPBH35TR-VT-7 |
| TATAGCAGTGCGAGGATGCTGTGGTTTTGGCAGACCACTGC |
| >CRISPR_II_metagenome_CYPBH35TR-VT-8 |
| ACCTAACATTAGGTATCCAAGTCTCATGCCGTTTATTTTGGC |
| >CRISPR_II_metagenome_CYPBH35TR-VT-9 |
| GCTTAAGCTGCCCTGGGCCAACCTGGCCAGTGGTAAAG |
| >CRISPR_II_metagenome_CYPBH35TR-VT-10 |
| TCGAATTACTTGAAAACCTTATGTACACTCTCAGATTACTTTAAA |
| >CRISPR_II_metagenome_CYPBM48TF-VT-1 |
| TACACCAAACGGTGCATGGCTGATCTATACTGATGCGG |
| >CRISPR_II_metagenome_CYPBM48TF-VT-2 |
| AGCATCATTGTTAATCAGATTTAATTTGAGTTGCTTTGTTAAG |
| >CRISPR_II_metagenome_CYPBM48TF-VT-3 |
| AAGAAGGGGAAAACGAAGATGGGTGGAACCTCATTAAT |
| >CRISPR_II_metagenome_CYPBM48TF-VT-4 |
| TTCCTTCGTATATACTCATGGAATAGCTTCTTACCTGC |
| >CRISPR_II_metagenome_CYPBM48TF-VT-5 |
| ATGCTCACCATCCGGAGAGCTTCCTGGATGGTTGTCTCAT |
| >CRISPR_II_metagenome_CYPBM48TF-VT-6 |
| TATAGATAATACGATTGAAGGTGTTTTTGTACCAGGT |
| >CRISPR_II_metagenome_CYPBM48TF-VT-7 |
| TATAGATAATACGATTGAAGGTGTTTTTGTACCAGGT |
| >CRISPR_II_metagenome_CYPBM48TF-VT-8 |
| TAGGGATGTAAACCCTAATTATACACTTGAGATTGATAATT |
| >CRISPR_II_metagenome_CYPBM48TF-VT-9 |
| GGCCTCACTCCCTTACGCAGGTGATAAAAGCACCTAAAT |
| >CRISPR_II_metagenome_CYPBM89TF-VT-1 |
| TCTGGATCTACAATGCAGTCGGAAAGATACTCATCTT |
| >CRISPR_II_metagenome_CYPBM89TF-VT-2 |
| GTGAAGGTTGGATCATAACTGACTAACCCCATTAAAGGCT |
| >CRISPR_II_metagenome_CYPBM89TF-VT-3 |
| GCAACGGTCTTACGACGAGTCGCCTCTTCCTGGCGTTGCTG |
| >CRISPR_II_metagenome_CYPBM89TF-VT-4 |
| GGCAAGCACTTTATTAACGGCAGAGTTGATTTCTTT |
| >CRISPR_II_metagenome_CYPBM89TF-VT-5 |
| AATGAGTCTCTTTCTGAAGTTCTCATCACGCAATTCATG |
| >CRISPR_II_metagenome_CYPBM89TF-VT-6 |
| AGTTGGGACGCCCGAATGGCTGCTTGCACAGCTGCCGGGTCTTTTT |
| >CRISPR_II_metagenome_CYPBM89TF-VT-7 |
| TGCCAATACGGCAGCCTTGTTGGGGTTAGATTAGTTGTA |
| >CRISPR_II_metagenome_CYPBM89TF-VT-8 |
| ACCTTCGGTGATCCCAACCACGTGGAACCGGCCAGCAC |
| >CRISPR_II_metagenome_CYPBM89TF-VT-9 |
| ACCTTCTGTGATCCCAACCACGTGGAACCGGCCAGCAC |
| >CRISPR_II_metagenome_CYPBS56TF-VT-1 |
| CTGTTGTGTAGGTTGACCTCCCCAACAAGACACCCAGGATT |
| >CRISPR_II_metagenome_CYPBS56TF-VT-2 |
| CTGAACCAAGCTCACTTGAGTGTTACTAAGTTGAATC |
| >CRISPR_II_metagenome_CYPBS56TF-VT-3 |
| GTAAAACAGTCGAACTCCCCGAGACGTAGGTTGC |
| >CRISPR_II_metagenome_CYPBS56TF-VT-4 |
| GGTTGGGCTGTTGCTAACCTAGATATCGCATGTGAGTGCG |
| >CRISPR_II_metagenome_CYPBS56TF-VT-5 |
| GTGGCTGATCGCCGCAGAAGTCTGGGGCCAGGTTACGATAG |
| >CRISPR_II_metagenome_CYPBU82TR-VT-1 |
| TGTTTTGCAAGTGAGGCTTTATACTCGGCTTCGAGCCGA |
| >CRISPR_II_metagenome_CYPBU82TR-VT-2 |
| ACACCCAGCCCAGGCCGCTGTAAAACCGGACGACAGCATAGT |
| >CRISPR_II_metagenome_CYPBU82TR-VT-3 |
| TGGGTGTGTTTGGGGAGGCTAGGTTAGGTTAGAGTG |
| >CRISPR_II_metagenome_CYPBU82TR-VT-4 |
| CTGGGCTGACCACTAGTGCAGCTCAACTTTGGGCAGTAGATA |
| >CRISPR_II_metagenome_CYPBU82TR-VT-5 |
| TCCCCCACCAACACCCAGGTATTTCACCTGGGTGTTGTTT |
| >CRISPR_II_metagenome_CYPBU82TR-VT-6 |
| ATCAACCTCCGATTTGGCAGGGAATGACGAAACCTGCCTT |
| >CRISPR_II_metagenome_CYPBU82TR-VT-7 |
| TCTTTTTTGATAGTCTGAAAGATCCTGTCTATTTGTCGAA |
| >CRISPR_II_metagenome_CYPBU82TR-VT-8 |
| TTATTTCTGGAGGACGAAACCGTACTCATCTTCAACAACTT |
| >CRISPR_II_metagenome_CYPBU82TR-VT-9 |
| CAAACAGGAGGTTAGCTATGGATATGTTGAATCCAGAAT |
| >CRISPR_II_metagenome_CYPBU82TR-VT-10 |
| TGCCACCTCCAGCTCCAGCAAGCCCGGCACTTGTTGTTTCAA |
| >CRISPR_II_metagenome_CYPC676TF-VT-1 |
| TAGAACCCCAAGAGGTAGAGGAGGAAGATGAGGAGCT |
| >CRISPR_II_metagenome_CYPC676TF-VT-2 |
| AATATTAAAATCAGCGAAGTATCTAAACTTATAAATTAAA |
| >CRISPR_II_metagenome_CYPC676TF-VT-3 |
| CGACCGAACGAACCGGCAGGCTTCGATCCAACTC |
| >CRISPR_II_metagenome_CYPC676TF-VT-4 |
| ATAACCCTCCTGCAGTACAACCCAGCTTCCTTGGA |
| >CRISPR_II_metagenome_CYPC676TF-VT-5 |
| TTTTCTTCTTTCGCGATTTAGATGAGTACTTGTCTC |
| >CRISPR_II_metagenome_CYPCA29TF-VT-1 |
| CGAATCGGGTGTTGAGTTTGTGGACGGTGAAACCATCT |
| >CRISPR_II_metagenome_CYPCA29TF-VT-2 |
| TTGGTGTCGGGTAGTTTACGGCTGCAACTGTATTGCAGA |
| >CRISPR_II_metagenome_CYPCA29TF-VT-3 |
| CTTCCTAAAATCAAAAACCCTCAGAAACTTCGAATCGGCCT |
| >CRISPR_II_metagenome_CYPCA29TF-VT-4 |
| AGGTTAGACTACTTGTCCGTCAGGGCTCGGTAGCAC |
| >CRISPR_II_metagenome_CYPCA29TF-VT-5 |
| ATCGCGGATGTACTCACAGCGCTCGCTTCTGAGGATTTGCT |
| >CRISPR_II_metagenome_CYPCA29TF-VT-6 |
| CGCTATACTTCGCCTGATTGCCGATGGGCCACTGGCCCT |
| >CRISPR_II_metagenome_CYPCM14TR-VT-1 |
| CCTACAGCACCTATACTACCCCCCTGCCCACCTAAA |
| >CRISPR_II_metagenome_CYPCM14TR-VT-2 |
| CTATTGTTACAAGGATGAGTTGCCGTGTCTCTACGCAGAATG |
| >CRISPR_II_metagenome_CYPCM14TR-VT-3 |
| CAGGGGGGTGGGAGCCGGCGCGTCCACTCACTGTCAC |
| >CRISPR_II_metagenome_CYPCM14TR-VT-4 |
| CTCAGTCGGTTTGGGATTGTTGACCCCGATGTTAA |
| >CRISPR_II_metagenome_CYPCM14TR-VT-5 |
| ATGACTACACTCCAGCCACGCTGGACTACAGTCAGCAA |
| >CRISPR_II_metagenome_CYPCM14TR-VT-6 |
| TGAGGATCTATTACTCTAGTCAAGAGGAGTACTTCGG |
| >CRISPR_II_metagenome_CYPCM14TR-VT-7 |
| TCCGGGAAATCTACAGCACTGTCCAAGTCCGAATGGTC |
| >CRISPR_II_metagenome_CYPCM14TR-VT-8 |
| ACCCAGATTGCTCTAGCCTTCGCTTCCGAAGTTGGA |
| >CRISPR_II_metagenome_CYPCM14TR-VT-9 |
| GTCCAAAGAGCTTACCAAAGCTCTAAAGGCTCAGGCT |
| >CRISPR_II_metagenome_CYPCM14TR-VT-10 |
| TTAGACAGGGAGACGCTCACGCGATCCCCATCATAGT |
| >CRISPR_II_metagenome_CYPCN60TF-VT-1 |
| TTTAGGCTGGGATGCTACCTCCTTCAACTTAGGCA |
| >CRISPR_II_metagenome_CYPCN60TF-VT-2 |
| TTCAACTCGCTGTAGTTACCACGATTGAGCCAATCGCT |
| >CRISPR_II_metagenome_CYPCN60TF-VT-3 |
| ACGAGATAAGTAAGTTCCGCTACGAGCGGGAGACGGATT |
| >CRISPR_II_metagenome_CYPCN60TF-VT-4 |
| CACGATGGGGCGGGCAAGTTGTCTTCTGTTAATTCACCAAGC |
| >CRISPR_II_metagenome_CYPCN60TF-VT-5 |
| GTGAGGTCCTCAGTTGCAGGAGACCAATCACTATGCAAA |
| >CRISPR_II_metagenome_CYPDL59TR-VT-1 |
| GGGAATTTTCTCTGCAACAACATCTGGCACCCTCCAGTAA |
| >CRISPR_II_metagenome_CYPDL59TR-VT-2 |
| GGTTACGGAGGACAACTGGCCAAACCCACAATGGGCGGTAGC |
| >CRISPR_II_metagenome_CYPDL59TR-VT-3 |
| ATCCAAGATCAACACCAACACTGTGGTTCTTAGGTGGAATA |
| >CRISPR_II_metagenome_CYPDL59TR-VT-4 |
| TCGAGAGAAGGCTCTTAGTCAAAGGGTATTTATCTGAGT |
| >CRISPR_II_metagenome_CYPDL59TR-VT-5 |
| TTGCGGTAGGGAACCGCAATTATGTAGTGGCCGTCAGTACCGGCA |
| >CRISPR_II_metagenome_CYPDL59TR-VT-6 |
| TCGCCGAACTGGCACTCGTAGGCCGCTTTGGCCATCTCG |
| >CRISPR_II_metagenome_CYPDL59TR-VT-7 |
| TTTAAACCGATCACTCCCTCGAATGGCTTTCGCCAA |
| >CRISPR_II_metagenome_CYPDL59TR-VT-8 |
| CGGCCAGGGAAGATGTACGAGTAGCCCATTAGTGCTACT |
| >CRISPR_II_metagenome_CYPDL59TR-VT-9 |
| CCTATAGCCACAAAAAATCCCCCGCCAATAAGGCGGG |
| >CRISPR_II_metagenome_CYPDM04TF-VT-1 |
| TTAAAGACCTCCCAGATCGCCGTTGCAGTGATCTCCA |
| >CRISPR_II_metagenome_CYPDM04TF-VT-2 |
| TTCAGAGAGCAATCCTCAAGGTATGCCTCAAAACCCG |
| >CRISPR_II_metagenome_CYPDM04TF-VT-3 |
| ACCAACAACCTCAACGAAATCATTTGGATCCAGC |
| >CRISPR_II_metagenome_CYPDM04TF-VT-4 |
| TCGCTCACAAAGCACGAAACCTTTGAGGGACTACCTTGT |
| >CRISPR_II_metagenome_CYPDM04TF-VT-5 |
| CAGCCTTTGAGGGAACTACAATCCGAGAGACTCTCG |
| >CRISPR_II_metagenome_CYPDM04TF-VT-6 |
| AACTATCTACACTCCAACTAAGGCGTTATTGTCCCACCAAGG |
| >CRISPR_II_metagenome_CYPDM04TF-VT-7 |
| AATCATCATAACTACCTCCCTTTACAGGGAATAAAGA |
| >CRISPR_II_metagenome_CYPDM04TF-VT-8 |
| CAACTTAGGTATTTCTCAATATTACTGAGGATTAG |
| >CRISPR_II_metagenome_CYPDM04TF-VT-9 |
| ACTCCTTATACATACTACAACAGCGGACACCGCTTCGTTA |
| >CRISPR_II_metagenome_CYPDM04TF-VT-10 |
| CGAAGATGGGTTTATCGTCTGGAATCAGACAAATCCCA |
| >CRISPR_II_metagenome_CYPDP07TF-VT-1 |
| ACCCCTCTCGGGCATTGGTGTGTATGCGGACAGTACT |
| >CRISPR_II_metagenome_CYPDP07TF-VT-2 |
| GTTTATCCCAATCGAAGGGAGGTTCGAATGAGAATAAA |
| >CRISPR_II_metagenome_CYPDP07TF-VT-3 |
| GCAATATCTGTGAAGTATTGAACTAACTGCATCGCAGTCAGT |
| >CRISPR_II_metagenome_CYPDP07TF-VT-4 |
| CATCGTGGTGGCTTTCTTTGAAGAGGACAACTAAATA |
| >CRISPR_II_metagenome_CYPDP07TF-VT-5 |
| AGTAACTCAAGAATCGGATTACTTATTGACGAAATTTT |
| >CRISPR_II_metagenome_CYPDP07TF-VT-6 |
| ATGTATTCGCAACGTTGGGCACGAATGATTTGCTCTT |
| >CRISPR_II_metagenome_CYPDP07TF-VT-7 |
| TTCATAGCATTCATGACTACCTCTCCCTTCAATTGGGAT |
| >CRISPR_II_metagenome_CYPDP07TF-VT-8 |
| CCCAACTTGGAGCGGGCCACCCATCTTGTGTGAGATCCTCCAG |
| >CRISPR_II_metagenome_CYPDP07TF-VT-9 |
| CTCTGCCTTACGAGCCCGCAGGGCGTCCAGGACAAAAT |
| >CRISPR_II_metagenome_CYPDP07TF-VT-10 |
| GCTAACGCAGTGTCCGCTACGCGTCCATTAATCCAG |
| >CRISPR_II_metagenome_CYPDP07TF-VT-11 |
| TAGTAGCTATCTCCTACCCTTTCTTCGTACGGGTAGGCCTG |
| >CRISPR_II_metagenome_CYPDQ72TR-VT-1 |
| CCGGGTTAGCGGGGGCCACTCCGGGAACAGAATACGA |
| >CRISPR_II_metagenome_CYPDQ72TR-VT-2 |
| GAGACACAGTTGAGGTGTGGCTAACTGCGCCCAATCAG |
| >CRISPR_II_metagenome_CYPDQ72TR-VT-3 |
| TGGCCAGGAGCCAATTGTAGAGGTAGCCCCTCACCGTTAGCTTGC |
| >CRISPR_II_metagenome_CYPDQ72TR-VT-4 |
| GGGAAACGGTTGAACGGGTCATACCAACGCAACGGATC |
| >CRISPR_II_metagenome_CYPDQ72TR-VT-5 |
| ACGATCTTGAACGGATTAGCCAACGGCGAGCCTTTTAGCCCTGG |
| >CRISPR_II_metagenome_CYPDQ72TR-VT-6 |
| TACTCAATTGCTGCGTCATCCCAACCTTCCTCGATG |
| >CRISPR_II_metagenome_CYPDQ72TR-VT-7 |
| AGAAACAGTCGCCCGCAAACGCCCCTGGACGCCCACAGT |
| >CRISPR_II_metagenome_CYPDQ72TR-VT-8 |
| TGCAGCGCGAAAGTAGCGCGGGTATTTTATTCATTT |
| >CRISPR_II_metagenome_CYPDQ72TR-VT-9 |
| CTCCTAAGGCAGGTGAACGACTACCCTGCCTATCTAAAAATA |
| >CRISPR_II_metagenome_CYPDR90TR-VT-1 |
| CTCGTAACAAACAGTCTGCGAGAGGCTGCAGAAATCGT |
| >CRISPR_II_metagenome_CYPDR90TR-VT-2 |
| ATTCCCTATGCAGCCCGCTGGCTGCTGATTGAGGAAC |
| >CRISPR_II_metagenome_CYPDR90TR-VT-3 |
| TTTCTTTCAATCAAATGCCTGCGTAGTATGACGAAG |
| >CRISPR_II_metagenome_CYPDR90TR-VT-4 |
| ATTAAAAGCGGTAGTGCAAGCCACGATCCGCTGAGCAAATTG |
| >CRISPR_II_metagenome_CYPDR90TR-VT-5 |
| AACCTATGACTACTACCACCATCTTCTCTCGTATTCCGG |
| >CRISPR_II_metagenome_CYPDR90TR-VT-6 |
| ACGGCGAGGCTCTGGCTAAAGCACATAAAATACATAGT |
| >CRISPR_II_metagenome_CYPDR90TR-VT-7 |
| CGTACTGCAAGGCCCCAGTGCCACGGGCCTAAATCAATTG |
| >CRISPR_II_metagenome_CYPDV63TR-VT-1 |
| TGCTAAGGGGACCTGCAAGCCCTGGGAGCACCAGCTA |
| >CRISPR_II_metagenome_CYPDV63TR-VT-2 |
| GATTGGGCAATCGGACGGGCATTCGGGTAGTCGGCG |
| >CRISPR_II_metagenome_CYPDV63TR-VT-3 |
| ATTTCTTCCATAAAAGCTATACAAAGGTCTTGAGA |
| >CRISPR_II_metagenome_CYPDV63TR-VT-4 |
| TAGAAGAACACGACTCGCAGAGGCTTATCCAGGGCCT |
| >CRISPR_II_metagenome_CYPDV63TR-VT-5 |
| TCTCCATCGCGGATTGCAATCCAATGGAAATTGAAAA |
| >CRISPR_II_metagenome_CYPDW52TF-VT-1 |
| ATTGAGGAGCTACCAAGCCTTCTACCATATCAGCAGGAT |
| >CRISPR_II_metagenome_CYPDW52TF-VT-2 |
| CTCTTCTTGAAGTGCGGGATGTATATGTACTGGACGGTAA |
| >CRISPR_II_metagenome_CYPDW52TF-VT-3 |
| CTGAGTCCTTGGTTTATTCGTGGCGGCCGTGTCGA |
| >CRISPR_II_metagenome_CYPDW52TF-VT-4 |
| TATTAGTGCGAAAAATAGGATGCTGCTCTGTCTTATACTTAAT |
| >CRISPR_II_metagenome_CYPDW52TF-VT-5 |
| GTAAAGACTGTTAGTAACCAGTCGCTTGCGAACGAATCGG |
| >CRISPR_II_metagenome_CYPDW52TF-VT-6 |
| TGTAGTACAGGGGTGTGTGGGTGATCCCGCCAGAA |
| >CRISPR_II_metagenome_CYPDW52TF-VT-7 |
| GGCAGGATGTAGCCCAGTCAGGACTGGGTATACCATCAG |
| >CRISPR_II_metagenome_CYPE216TR-VT-1 |
| TGCATACATGGCCGAAGAGGCCTATGCGGCTACGTTCGG |
| >CRISPR_II_metagenome_CYPE216TR-VT-2 |
| AAGATGGGTAATTAGCTGGTAGTCAGCGATCCCCA |
| >CRISPR_II_metagenome_CYPE216TR-VT-3 |
| TGCGACGAGTTCGAATCGCTGAGTAGAGCCTTGACCAT |
| >CRISPR_II_metagenome_CYPE216TR-VT-4 |
| ACGCGGTGATCGTCTGGGGACGGGGACGCTGGGTGAT |
| >CRISPR_II_metagenome_CYPE216TR-VT-5 |
| TCAGCTTTAGTTTGTGGACACGGTTGGCATAATTTCTA |
| >CRISPR_II_metagenome_CYPE216TR-VT-6 |
| AAAACTGCTACCAAGCCATGTTCGGCGCACCAGAGTC |
| >CRISPR_II_metagenome_CYPE216TR-VT-7 |
| CTAACATATGTCCCATGGTACCACGCGGCCCGATT |
| >CRISPR_II_metagenome_CYPE874TF-VT-1 |
| ACCAAATACAGTGGTTTGCCAGAACCATAGTCTACCT |
| >CRISPR_II_metagenome_CYPE874TF-VT-2 |
| CCCGGGGCGGGTCTTACGGGCTTGGGTAGCAAAGCAGGAA |
| >CRISPR_II_metagenome_CYPE874TF-VT-3 |
| TTGGAACCAACTCGGTCTGGTTACATTCTGACCTCCT |
| >CRISPR_II_metagenome_CYPE874TF-VT-4 |
| ACTGGAACGGGGGACAGCTCTCTCAGACCGAACGGTTTA |
| >CRISPR_II_metagenome_CYPE874TF-VT-5 |
| CTTCATACGATTGTGCAGTTGACAGAGACTCTTTAACC |
| >CRISPR_II_metagenome_CYPE874TF-VT-6 |
| TCCGGAGTCGGTTGATACACCGAACCAGAAAAAGTTCGACAGTA |
| >CRISPR_II_metagenome_CYPE874TF-VT-7 |
| AACACCCAGGTATCTTCACCTGGGTGTTGTTTCTATCCCGATAAG |
| >CRISPR_II_metagenome_CYPE874TF-VT-8 |
| AAGAGGTAGAGGAGGAAGATGAGGAGCTGCAGGAGCTAA |
| >CRISPR_II_metagenome_CYPE874TF-VT-9 |
| CTGCCACTTCTGTGGCAGCCTTTGATGAGATAATTACCGCA |
| >CRISPR_II_metagenome_CYPEB70TF-VT-1 |
| ATAGGCCAAACTCGTTCGATCTCTCTTCAAGAGATTTTGAGT |
| >CRISPR_II_metagenome_CYPEB70TF-VT-2 |
| TTTCCAAAAAGACAAGATTGGCTAGAACAGGCTCTGGCAA |
| >CRISPR_II_metagenome_CYPEB70TF-VT-3 |
| ACACACCATTAATGAAGTAATGACGGGGTTAAACCTA |
| >CRISPR_II_metagenome_CYPEB70TF-VT-4 |
| TTGGGTAGAGCATGCAGCTCTAATCCTGGACTAACCCC |
| >CRISPR_II_metagenome_CYPEB70TF-VT-5 |
| GTCGGAGAGTTCCGCCAATAACTTGGCGGAAAAGTTGTAAGTAGAAA |
| >CRISPR_II_metagenome_CYPEB70TF-VT-6 |
| CACGCCGACTACCTCCATGTAGTCGGAGAGTTCCGCCAA |
| >CRISPR_II_metagenome_CYPEB70TF-VT-7 |
| CGTTTGATCCATATTGGATCATGTTAGAACACGGTT |
| >CRISPR_II_metagenome_CYPEB70TF-VT-8 |
| GTTAGAGCGAGTGAACAACTCCAGCTTACCGTCTACTT |
| >CRISPR_II_metagenome_CYPEB70TF-VT-9 |
| AACGGAGGCTTGACCTCCACATCCAGCAGTGAGGAT |
| >CRISPR_II_metagenome_CYPEB70TF-VT-10 |
| CTTCCCTGCCCCCCGGGGAGTAGACCATCTCATTCTCTT |
| >CRISPR_II_metagenome_CYPEB70TF-VT-11 |
| TTCCGCAGCAGCATTGCCTCCAGCCGCTGCAGCAGTTGCTCC |
| >CRISPR_II_metagenome_CYPEK25TR-VT-1 |
| CCCCACGCGCTTCAAGGTCGCTGCGCTCCCTCTG |
| >CRISPR_II_metagenome_CYPEK25TR-VT-2 |
| ACGTGCGTAGTTGCTGAATAGTTGCACGTGTAACGT |
| >CRISPR_II_metagenome_CYPEK25TR-VT-3 |
| ACTGAATAATCAGTCTGGATACACCGAAGATGTTACCAATG |
| >CRISPR_II_metagenome_CYPEK25TR-VT-4 |
| AGCAACATCCCTAATATATTCGCAGCGTGTGCTGCGAAT |
| >CRISPR_II_metagenome_CYPEK25TR-VT-5 |
| CATCAAACAGCATCCGCGAGGCATACGAAGTCCTCAAAGAAG |
| >CRISPR_II_metagenome_CYPEK25TR-VT-6 |
| TAAACCGTCCTCCGCTCTGGAGACATGGCGACTGCTGCGAAGT |
| >CRISPR_II_metagenome_CYPEK25TR-VT-7 |
| TTAATTGTCGTTCCTGGTAAATAGTTATCTTGTGCG |
| >CRISPR_II_metagenome_CYPEK25TR-VT-8 |
| ACCAAGTGCAAACTGCATTCATCGAATGGGGGCATC |
| >CRISPR_II_metagenome_CYPEK25TR-VT-9 |
| TCCCGATTCAAAGGCTGCCACTAAGGCAGCCTTTTT |
| >CRISPR_II_metagenome_CYPEK25TR-VT-10 |
| ATGACCCAGTTGAGCCCGCCTTTTACTTTACTTCCAAG |
| >CRISPR_II_metagenome_CYPEU81TR-VT-1 |
| CATCGTCATAGCTGATGACAGCGAAACTGCCCCACCTT |
| >CRISPR_II_metagenome_CYPEU81TR-VT-2 |
| CCCTCTGCTTGAAAATGGTCAAACGTTATATGCAC |
| >CRISPR_II_metagenome_CYPEU81TR-VT-3 |
| TCTGGGAGTTACCCAGGATTATTCAGTTGTAATACT |
| >CRISPR_II_metagenome_CYPEU81TR-VT-4 |
| AATTCTTCCCAGGCTTCTGAGTATAGTTGCCTGAT |
| >CRISPR_II_metagenome_CYPEU81TR-VT-5 |
| TCTTTCTTCCCGGTTAAGGGAGAGGAGTTTGTTATGCATA |
| >CRISPR_II_metagenome_CYPEU81TR-VT-6 |
| ACTCCTGCTCGTCTGCTATCATGACTCTCGTAATAATGGGAAA |
| >CRISPR_II_metagenome_CYPEU81TR-VT-7 |
| GGGCTGGGAGCCGACATCACTACGCCATCGTTCAGCTT |
| >CRISPR_II_metagenome_CYPF753TF-VT-1 |
| TTCAAAGTTCCAATAGAATTGTTTCCCGTTGGATAC |
| >CRISPR_II_metagenome_CYPF753TF-VT-2 |
| TTTTTGAGCTAACTCCAGGCTCGTTAGATTAGATTGGCC |
| >CRISPR_II_metagenome_CYPF753TF-VT-3 |
| CACAACTCCGGGAGCTGACCGTATGGTCAGTGAAAGACCGG |
| >CRISPR_II_metagenome_CYPF753TF-VT-4 |
| TTCTCTTCTCTACGAGAGTACGTAGGAGCAAAGAAATG |
| >CRISPR_II_metagenome_CYPFP72TF-VT-1 |
| CCCAACTTGGGTCGGGCCACCCATCATTGGTGACCTCCT |
| >CRISPR_II_metagenome_CYPFP72TF-VT-2 |
| TTAATGGACAATCGCGACCACTTCGGGTAGGATCACT |
| >CRISPR_II_metagenome_CYPFP72TF-VT-3 |
| TTCCAGAGGCCTTCCTGGGCATTTTAGAGGCATTCCAG |
| >CRISPR_II_metagenome_CYPFP72TF-VT-4 |
| TGGATGATCCGGATTTACTTATTAGTATTGCCGGAT |
| >CRISPR_II_metagenome_CYPFP72TF-VT-5 |
| TTGGTACTTCCTGCGCATCCTCGGTGCGGACTACATGCTA |
| >CRISPR_II_metagenome_CYPFP72TF-VT-6 |
| AAAATAAAAAGGAGAGTAATTAGAGTTTGCACTCTAGC |
| >CRISPR_II_metagenome_CYPFP72TF-VT-7 |
| GACTTAGGTTAGTTTGAATATAAACTTACTTTGCATCGT |
| >CRISPR_II_metagenome_CYPFP72TF-VT-8 |
| ATAGAGCTAGTTGAGTATGTAGGTCAAAAAATTAAG |
| >CRISPR_II_metagenome_CYPG183TF-VT-1 |
| TCTAGCGGCAATTCGCTTTGTCCGCAGTTACCTGAA |
| >CRISPR_II_metagenome_CYPG183TF-VT-2 |
| ATTGGTTGTATAGACAATACTCGGCTTAACAATTCAG |
| >CRISPR_II_metagenome_CYPG183TF-VT-3 |
| TACCACGAAAGCAAGCATTACTGAGCCTTATGTAAGGCCC |
| >CRISPR_II_metagenome_CYPG183TF-VT-4 |
| TACATTTTACGACCACCAATCTGGCGCACTATAAGCT |
| >CRISPR_II_metagenome_CYPG183TF-VT-5 |
| GGTTCATTGTGAGTATTCAAAATGGACATAGCTTCTTCATACGA |
| >CRISPR_II_metagenome_CYPG183TF-VT-6 |
| TCCACCCCAGCCGCATCCCCTACGTGCAGGTGGCAGT |
| >CRISPR_II_metagenome_CYPG183TF-VT-7 |
| CTCAGAGGGTATATCTTTGGAAGGCTGCGAGTCATCGAGTAC |
| >CRISPR_II_metagenome_CYPGW04TF-VT-1 |
| ATCTTTTCCCCGTAGTAGCGTGGATAGTTCTGCTG |
| >CRISPR_II_metagenome_CYPGW04TF-VT-2 |
| GACTAGTGCACTCTTGGCCTTTATGTCCGCATCGCCGAC |
| >CRISPR_II_metagenome_CYPGW04TF-VT-3 |
| ATATTGACACCGTTCAGCGCGAATGATTTGCTCTTTGGT |
| >CRISPR_II_metagenome_CYPGW04TF-VT-4 |
| GGGCATCAGGTCCTCCGGTTCGAGTAGTACAACATCGA |
| >CRISPR_II_metagenome_CYPGW04TF-VT-5 |
| AACGGAGGATAAAGAACACCCAGGTGAAAATACCTGGGTG |
| >CRISPR_II_metagenome_CYPGW04TF-VT-6 |
| AGCAGTTGCTTGAGTAATCATTGAGATAGGTCTAAAT |
| >CRISPR_II_metagenome_CYPGW04TF-VT-7 |
| ATTAAGTAGCTGTACAGGGGTTTTAGGTCGAAATAAAGC |
| >CRISPR_II_metagenome_CYPGW04TF-VT-8 |
| ACGAAAAGAAGTTTTCCACCATTGGCTCCGCACTCTTGGG |
| >CRISPR_II_metagenome_CYPGW04TF-VT-9 |
| CACCCAGGATTAATTCCTGGGTGCTTAGGGGGAGGT |
| >CRISPR_II_metagenome_CYPH645TF-VT-1 |
| TCCCACTAACCCAGACGAATGCCTCCCGCTCAGTTGTC |
| >CRISPR_II_metagenome_CYPH645TF-VT-2 |
| CGGGCACCGTTTGGTCATACGGACCCAACGCTGTTGAG |
| >CRISPR_II_metagenome_CYPH645TF-VT-3 |
| GCACGGTAACACTCGTAAGCTTCTCTGATGCTATTTGTGACTA |
| >CRISPR_II_metagenome_CYPH645TF-VT-4 |
| TCGTTATTATTGTTGACTGCAACCCACATCCCTGGGT |
| >CRISPR_II_metagenome_CYPHA03TR-VT-1 |
| AACCTATATCCCGTGGTATCACGCGGCACGGCTACTAGACTT |
| >CRISPR_II_metagenome_CYPHA03TR-VT-2 |
| AACCTATATCCCGTGGTATCACGCGGCACGGCTACTAGACTT |
| >CRISPR_II_metagenome_CYPHA03TR-VT-3 |
| AACCTATATCCCGTGGTATCACGCGGCACGGCTACTAGACTT |
| >CRISPR_II_metagenome_CYPHA03TR-VT-4 |
| ACGGTAAGTGGTTTTGGGCTAATCCAGGCTCTATTGTTGT |
| >CRISPR_II_metagenome_CYPHA03TR-VT-5 |
| CCTGCCGAAGGAGGAGGTAATATGACTACACTCACCCA |
| >CRISPR_II_metagenome_CYPHA03TR-VT-6 |
| ATTCATGGACAACGGGGTACGCTTCTTATATTTCAATA |
| >CRISPR_II_metagenome_CYPHA03TR-VT-7 |
| CTTCTACCAGTTCTGCGTAGGCGGAAAACCACTGACTACG |
| >CRISPR_II_metagenome_CYPHA03TR-VT-8 |
| TCTTCAGGGTTCAGGTAAATGTCGTTACCTTCGGTGA |
| >CRISPR_II_metagenome_CYPHF94TF-VT-1 |
| TTCCTAAGATCTTCGCAATGTCACGGATGTACTCGCA |
| >CRISPR_II_metagenome_CYPHF94TF-VT-2 |
| TTTAGGCGTGTCCATTGAGCAGATTCGGCGGGTTGAAGTTT |
| >CRISPR_II_metagenome_CYPHF94TF-VT-3 |
| CTGGATAATTTTCCAGGACGGTTTAACCTGCGATGG |
| >CRISPR_II_metagenome_CYPHF94TF-VT-4 |
| TATCACCCGTTTGTATGACCCATCCTACACGGGGGA |
| >CRISPR_II_metagenome_CYPHF94TF-VT-5 |
| GGAATGCTAGGGGTGTGCCTGGGACATTCCTGGAGT |
| >CRISPR_II_metagenome_CYPHF94TF-VT-6 |
| CCCCAATTATGTGGGCGACCAGATCCTTTTCCTCGAGGGT |
| >CRISPR_II_metagenome_CYPHF94TF-VT-7 |
| GGCCTCGCCACGGCTGCTGCTTTGATCTGGGC |
| >CRISPR_II_metagenome_CYPHF94TF-VT-8 |
| ATGAAAATAGTAATGACAGGTTCCATTTTTACCTCCCTTCTT |
| >CRISPR_II_metagenome_CYPHF94TF-VT-9 |
| GTGGTACCGAAACCCTGAGTGGGCAGACGATGAGTTA |
| >CRISPR_II_metagenome_CYPHM76TR-VT-1 |
| ATGAGATGGCCGACATGGCCGAGGCCTGCTATCAGGT |
| >CRISPR_II_metagenome_CYPHM76TR-VT-2 |
| GAGGAGTATTGAAACAACTCAAAACTGTCTTAATACTC |
| >CRISPR_II_metagenome_CYPHM76TR-VT-3 |
| TTCTAAAATAAAAGGAGTAAGCCTCACACAACACTTGTAT |
| >CRISPR_II_metagenome_CYPHM76TR-VT-4 |
| AACGGGAGCCTCCTTAAGGGAGAACCCATTAATCTTAAA |
| >CRISPR_II_metagenome_CYPHM76TR-VT-5 |
| GGAATTCGACAGCGAGTTCGATATCATCTAACCCAACAC |
| >CRISPR_II_metagenome_CYPHM76TR-VT-6 |
| ATCAACTAGTTCAAAGTCTAGTCCTTCATACAAGTACT |
| >CRISPR_II_metagenome_CYPHM76TR-VT-7 |
| ATCCAACCTAAAGACACATTTGGATTGAGACTCTTTGGAT |
| >CRISPR_II_metagenome_CYPHM76TR-VT-8 |
| TTCCCTGCGGTGGCCTAGGTTGGGATGCTACCTCCTTCA |
| >CRISPR_II_metagenome_CYPHM76TR-VT-9 |
| CTACCAGAACTTGAAGACTTCGCTGGGCTTCCCAATCCTGTTCC |
| >CRISPR_II_metagenome_CYPHT46TF-VT-1 |
| TCTGAAATACAGGGAAAAGCCCTGGGGTAAAGTATTCAC |
| >CRISPR_II_metagenome_CYPHT46TF-VT-2 |
| AAGTATATCCGGGCATTGTTATGCCCTTATATAGGG |
| >CRISPR_II_metagenome_CYPHT46TF-VT-3 |
| ATGAACCCGGGTGCCGAAGGGGAGAATAACCTAGAT |
| >CRISPR_II_metagenome_CYPHT46TF-VT-4 |
| ATACCAATACGTTCCAAACTTCGAGGCAAAAGTTCAGTGTT |
| >CRISPR_II_metagenome_CYPHT46TF-VT-5 |
| TGCCATTAAGCTCGTTGGGCCCTGGATCATCGCAAACAACT |
| >CRISPR_II_metagenome_CYPHT46TF-VT-6 |
| AGGTGCGTCCAAAGCACTCGCCACTGCAACTACAGTGTTGTCCA |
| >CRISPR_II_metagenome_CYPHT46TF-VT-7 |
| TTCCTTGAGAAGTACTTTAGACCTACTACAATTGCAC |
| >CRISPR_II_metagenome_CYPHT46TF-VT-8 |
| TAACACCCGGATAGTCCACAGTTGCACGGTACAAAGT |
| >CRISPR_II_metagenome_CYPIE52TR-VT-1 |
| GTCGGCCAACTGGATCTCTACAGCGTCTAGCTGAGCGC |
| >CRISPR_II_metagenome_CYPIE52TR-VT-2 |
| GTCGGCCAACTGGATCTCTACAGCGTCTAGCTGAGCGC |
| >CRISPR_II_metagenome_CYPIE52TR-VT-3 |
| TCGCGAGTGCACAAGCAATCGTGCCCGTCTTGAC |
| >CRISPR_II_metagenome_CYPIE52TR-VT-4 |
| TATCCAATCCCACATCAATACCCACCGCCCTTTTGACCG |
| >CRISPR_II_metagenome_CYPIE52TR-VT-5 |
| CCTTGACTGCTTTGGCTTTGACGGGCTGGCCTTTCTCGTT |
| >CRISPR_II_metagenome_CYPIP26TF-VT-1 |
| TGGATTTCCGCCGCTCACGACAGCCAAGTATCAAA |
| >CRISPR_II_metagenome_CYPIP26TF-VT-2 |
| TATGATGGTCTGCAACCACCACAACAGGATCCACAACAT |
| >CRISPR_II_metagenome_CYPIP26TF-VT-3 |
| TTCAACGATTGTTTCCTCCGGGAGATACCTGAGTGGGTT |
| >CRISPR_II_metagenome_CYPIP26TF-VT-4 |
| TGTACTCGATTACCTCGCCACTGGCCGTTCCTTCCTCT |
| >CRISPR_II_metagenome_CYPIP26TF-VT-5 |
| TTCTGACCTCCTTGTTCTCAGACTGTTCAAGATGTAGTCG |
| >CRISPR_II_metagenome_CYPIP26TF-VT-6 |
| CCCAATTTGTACCAAATTTTTGAGTGTGGAATATTGTTATC |
| >CRISPR_II_metagenome_CYPIP26TF-VT-7 |
| TGTTCACAGTGGATACGCTAGCTAAAGCACTTGCA |
| >CRISPR_II_metagenome_CYPIP26TF-VT-8 |
| GGCATGATTGTTCCCCTATCTTTAAACCTGGATGGTA |
| >CRISPR_II_metagenome_CYPJ361TR-VT-1 |
| GCCAATACTCCTCAGGTAGGAGTCTGACAAGGAAGCCGAA |
| >CRISPR_II_metagenome_CYPJ361TR-VT-2 |
| CGTCAAAACAGGTTCCATACTCAACCTCCCTTTACAGGGTA |
| >CRISPR_II_metagenome_CYPJ361TR-VT-3 |
| CGACTCGCCAGTCGGAGCCTTAGAAACTGGATGGTTTCTAAGAA |
| >CRISPR_II_metagenome_CYPJ361TR-VT-4 |
| CTGATATGCCTTCACCCTTGGGGGTGGGAACCACCCTTACAGGCT |
| >CRISPR_II_metagenome_CYPJ361TR-VT-5 |
| ACCCCATAGAATACAGCAGTGGTAGTCATATATCCTCCTAA |
| >CRISPR_II_metagenome_CYPJ361TR-VT-6 |
| TTGAAGATAAATTCATTACTCCTATCATTAAA |
| >CRISPR_II_metagenome_CYPJ361TR-VT-7 |
| TTAACTAGGGAAAACATCCACTCCGTTGGAGAGTGTATTTG |
| >CRISPR_II_metagenome_CYPJ361TR-VT-8 |
| TCCCGTTTAAAGGCGGCTTAAAAGGCTTTTAAAGGGCTTT |
| >CRISPR_II_metagenome_CYPJ361TR-VT-9 |
| GAGAATCACATACCATTCTCGCCGGCGAGAATGGAAAGCACTAGAACTGTTTGATTGCTA |
| >CRISPR_II_metagenome_CYPJ513TR-VT-1 |
| TAGTTAAGGGAGGTACATATGAATAAGGAAATTAAGCTCG |
| >CRISPR_II_metagenome_CYPJ513TR-VT-2 |
| TTCATCGATGACGAGACACTCTTCGTCTCTAATAGTCTCG |
| >CRISPR_II_metagenome_CYPJ513TR-VT-3 |
| ATGGCCCATGATGGCGATGGCCAGCCATTCTCATTA |
| >CRISPR_II_metagenome_CYPJ513TR-VT-4 |
| TTACGGATACCTAAGGCATAGAGAATCTCGTTAGCCTCGC |
| >CRISPR_II_metagenome_CYPJ513TR-VT-5 |
| GTACTCCCCGAAGTTGGGGTCACACTGGTTTACACCCCTGA |
| >CRISPR_II_metagenome_CYPJ513TR-VT-6 |
| TTTCCTTATCGCGGCGAGACACTGTCTCGAAATGGGCTGCCTGCAA |
| >CRISPR_II_metagenome_CYPJ513TR-VT-7 |
| ACGCGGGATGCGGCTCCGACTATACCGGGCATTCGGGGTTA |
| >CRISPR_II_metagenome_CYPJ513TR-VT-8 |
| AGCTCTTCGTCCTCCTCTACGTCTTGAGACTCAACATCTCTCGAAG |
| >CRISPR_II_metagenome_CYPJ513TR-VT-9 |
| TGCTTAGTGCATTGACAAGTTCGAACATTGTTACCT |
| >CRISPR_II_metagenome_CYPJ513TR-VT-10 |
| ACAGTCGCGCAGGTACTTGCGCTTACTGCTGTAGATAA |
| >CRISPR_II_metagenome_CYPJK82TF-VT-1 |
| GAACTGTCCGCTTCTCTAGCCTAAGCCCCCTTGTCAAAAGGAGAGCC |
| >CRISPR_II_metagenome_CYPJK82TF-VT-2 |
| GAACTCCATTAAGGCTCGTGAGGAGGAAGCAGCCCTCCGC |
| >CRISPR_II_metagenome_CYPJK82TF-VT-3 |
| GAACGGGAAATCAAGATCCATCGTCCGCCGGTCGAGCTGGTCAAG |
| >CRISPR_II_metagenome_CYPJK82TF-VT-4 |
| GAACAACAGGTGTTGGAACTGGGATCCGATCCCGACCAACTGGA |
| >CRISPR_II_metagenome_CYPK078TF-VT-1 |
| ATTGGATTACTTAAGCGCGGAGGGCTTGAGTTATCCTTT |
| >CRISPR_II_metagenome_CYPK078TF-VT-2 |
| AAGTCTGGAGTTACCTCCCTGGTACTAACTACCCA |
| >CRISPR_II_metagenome_CYPK078TF-VT-3 |
| TGGACGTACACGTTGTGGGTACAGCCGGAGTTCTT |
| >CRISPR_II_metagenome_CYPK078TF-VT-4 |
| ATGAAGGGATCCCTGATTGGCTCTGGGACAGTGGC |
| >CRISPR_II_metagenome_CYPK078TF-VT-5 |
| TGTAGTACATGGCAGACTCCTCCCTTTTCAGGGTAGAAA |
| >CRISPR_II_metagenome_CYPK078TF-VT-6 |
| GGAAAAGTGAGCTGGATCGACGCCGGTCGCTTCATTCGAGC |
| >CRISPR_II_metagenome_CYPK078TF-VT-7 |
| GTGAGGTTTCTGTATGGCTGACAAGCGTGTCTTGATTAAC |
| >CRISPR_II_metagenome_CYPK078TF-VT-8 |
| GAAGGGATATGGTATCTCGACGAGGTAGGTACTCTCGA |
| >CRISPR_II_metagenome_CYPK078TF-VT-9 |
| ATTTTAGAAAACGGTATTTACAGCATGATTGATCTCAGGG |
| >CRISPR_II_metagenome_CYPK266TR-VT-1 |
| CGATTCAACCACCCATCATACCCTTCGGGTATTTCAGGGCA |
| >CRISPR_II_metagenome_CYPK266TR-VT-2 |
| ACTCCTAGCTGCGGACTATATGTTAGTCCGCGCTATCGG |
| >CRISPR_II_metagenome_CYPK266TR-VT-3 |
| TTCTTGCCAGGCTATTTGAGCCTTCGAGAGAAGGCTT |
| >CRISPR_II_metagenome_CYPK266TR-VT-4 |
| TTTGGTCATTTTGACCCACCGCTGCCGTTGGGAACACCAG |
| >CRISPR_II_metagenome_CYPK266TR-VT-5 |
| AAGAAGCTTGATTCGCATAACAGACTCCTCCCTT |
| >CRISPR_II_metagenome_CYPK266TR-VT-6 |
| GCTACTCGTATATCTTCCCCGGCAAAGGGCTGAAGCC |
| >CRISPR_II_metagenome_CYPK266TR-VT-7 |
| TACGGCCATCCAGCCCAGTGCCCCTATTGACCCCGAT |
| >CRISPR_II_metagenome_CYPK266TR-VT-8 |
| CTGGTACTGAATTACAATACCGTCGTGACCTTCTTCCAGAA |
| >CRISPR_II_metagenome_CYPK266TR-VT-9 |
| AACGGTGGGCTTCACTCCGTACTCGCGAAGCTGCTTCCGC |
| >CRISPR_II_metagenome_CYPK266TR-VT-10 |
| AGTACATCCCGAGCTGGTGTAAAATGGGTAGCACGAT |
| >CRISPR_II_metagenome_CYPK565TR-VT-1 |
| GCTCACGACAGGTGAGCATAAGGCGGAGCATATAGTGGGT |
| >CRISPR_II_metagenome_CYPK565TR-VT-2 |
| GTCCTTATAGTATCTTGTTTCAACGTATAAATAATCAG |
| >CRISPR_II_metagenome_CYPK565TR-VT-3 |
| ATAAACTTCTGAGAAAAGAAAGTACCGTAATCAACCCAGT |
| >CRISPR_II_metagenome_CYPK565TR-VT-4 |
| CACCGAGAGTCTGAGGATTCCTCAAGTATGCTGGGTA |
| >CRISPR_II_metagenome_CYPK565TR-VT-5 |
| TCGATTATCTCGTTACCATCACTCTCCTCAATGATA |
| >CRISPR_II_metagenome_CYPK565TR-VT-6 |
| TCTGAAACTGACCTAGAAGCATGGGGAATGTACATAGG |
| >CRISPR_II_metagenome_CYPK565TR-VT-7 |
| GACAAAGACCACACGTATTCGTGTGTACTGTCTAGC |
| >CRISPR_II_metagenome_CYPK565TR-VT-8 |
| ATCTAGCCCATTGTGGGATGAAGATGCATTTGGTGT |
| >CRISPR_II_metagenome_CYPK565TR-VT-9 |
| ACACTAGGGCGACCAGTACAATTGTACCCCTCCTCCA |
| >CRISPR_II_metagenome_CYPK570TF-VT-1 |
| CAGCAGCGACTGGAGAGGTTCAAGGGTGGCGATGGGAT |
| >CRISPR_II_metagenome_CYPK570TF-VT-2 |
| ATGGGGACGGAGACGGAGTGTGGGTATTGCTCTTCGT |
| >CRISPR_II_metagenome_CYPK570TF-VT-3 |
| TGAGTTTGTAGTGATGGAGGGACGTTGATGCTGAGT |
| >CRISPR_II_metagenome_CYPK570TF-VT-4 |
| TTGAAGACTCTCTTACTAACAAACCGTGGGCGTAGGCCAA |
| >CRISPR_II_metagenome_CYPK570TF-VT-5 |
| ACCTCTCACAGAACTTAGATAGAATCCAACCAGGAA |
| >CRISPR_II_metagenome_CYPK570TF-VT-6 |
| TACTCAATAACCTTATTGTACGCCTCTTCCAAGTCTTGTT |
| >CRISPR_II_metagenome_CYPK570TF-VT-7 |
| ATCAGGATCCAGTATGGATCGTACGGAACTACGATCAT |
| >CRISPR_II_metagenome_CYPK570TF-VT-8 |
| GTGAGCGCTTTAGACTTACGAGCTACGTTCTTACGT |
| >CRISPR_II_metagenome_CYPK570TF-VT-9 |
| AATTCGACAAAGTGTTCTTTGAAAGTCTGCTTGCTAAC |
| >CRISPR_II_metagenome_CYPKD67TR-VT-1 |
| TTTGTGCCCTTATGGCTCCCGTTGGCCCAGGTCTTCGGGTGCCC |
| >CRISPR_II_metagenome_CYPKD67TR-VT-2 |
| TATATCATCCATCAGGACACAGAGGTAATCCATATAAAG |
| >CRISPR_II_metagenome_CYPKD67TR-VT-3 |
| CGTAGTCAGTGGTTTTCCGCCTACGCAGAACTGGTAGAA |
| >CRISPR_II_metagenome_CYPKD67TR-VT-4 |
| TATTGAAATATAAGAAGCGTACCCCGTTGTCCATGAA |
| >CRISPR_II_metagenome_CYPKE37TF-VT-1 |
| GGTGTTCTTTCTACTCCGTTAAGGGAGGAGTCTATATGGA |
| >CRISPR_II_metagenome_CYPKE37TF-VT-2 |
| TCGCGGATATACTGACACCGTTCAGCGTGAATGATTTGC |
| >CRISPR_II_metagenome_CYPKE37TF-VT-3 |
| TTAGCCGCCTTGAGCACGACATCGCCATGACAAGGCAGAGGCGC |
| >CRISPR_II_metagenome_CYPKE37TF-VT-4 |
| TGTAATCAAAGAACTCTTCGTCGCTGCAGACTTTGATCT |
| >CRISPR_II_metagenome_CYPKE37TF-VT-5 |
| TGCCTAACCTAAGCTGGACAGTCGCCTGCTTGATCTAGA |
| >CRISPR_II_metagenome_CYPKE37TF-VT-6 |
| TTTTAATAGAGCTTTGAAATTAGGAGCTAGCTCTGACCTTA |
| >CRISPR_II_metagenome_CYPKE37TF-VT-7 |
| TTCAGTTTGCTGATCTGGTTGGAACCCAGCTTAGTGC |
| >CRISPR_II_metagenome_CYPKE37TF-VT-8 |
| CGTTTGGATTATAACAGTATTCGACTACAATGCTCGGT |
| >CRISPR_II_metagenome_CYPKE37TF-VT-9 |
| GCCAACCTCCTAAATGTTTATAGACCTAGAAATAAAAAAGAGGG |
| >CRISPR_II_metagenome_CYPKX54TF-VT-1 |
| TTTGAGGAGTACAAGAAAGTCTGTCAAATCTCCGGTC |
| >CRISPR_II_metagenome_CYPKX54TF-VT-2 |
| TTCGTGACAACAATGGCCAGCCCATCGGATGCAAAGGCTTCCGTAGG |
| >CRISPR_II_metagenome_CYPKX54TF-VT-3 |
| CCTCCCCGCGAAAAGGGAAGGAGTTAAGATTAAAGAG |
| >CRISPR_II_metagenome_CYPKX54TF-VT-4 |
| AGGAACGGCCAGTGGCGAGGTGATCGAATACACCTACACTCG |
| >CRISPR_II_metagenome_CYPKX54TF-VT-5 |
| TTGTGCTAGAGGAGGGCTGGAATCGCGTTGGTAAACC |
| >CRISPR_II_metagenome_CYPKX54TF-VT-6 |
| TGCGCATAGAGCGCGGAGAAGTTAGGGAGGCTTTCGAG |
| >CRISPR_II_metagenome_CYPKX54TF-VT-7 |
| AGCATCCCAGCCTAAGCCACCACAGGAGACTACAGAGTTCTA |
| >CRISPR_II_metagenome_CYPL031TF-VT-1 |
| ACTGCGGCTGTCCGAAAACTTGGGGCAATGGCCGACAC |
| >CRISPR_II_metagenome_CYPL031TF-VT-2 |
| GCACTAGTGGGCAGCTCACTAGGGAATGCTCCCTTAGGCACG |
| >CRISPR_II_metagenome_CYPL031TF-VT-3 |
| TTGGTAACTAACAGCCTGAGGGAAGCGTACGAATGCTAC |
| >CRISPR_II_metagenome_CYPL031TF-VT-4 |
| TGCATATGCTCGTAGATATTCGTTGAGGCTGTTAGAG |
| >CRISPR_II_metagenome_CYPL031TF-VT-5 |
| GCTAACCTAGTTTATAGACATAGTTGAGACTGTTCTTTT |
| >CRISPR_II_metagenome_CYPL031TF-VT-6 |
| TGGCTGAGCGCGGAGCGGCGCCGGCAACGCCAACTCGACCAAA |
| >CRISPR_II_metagenome_CYPL031TF-VT-7 |
| TCTTAAAGTTAAATGAGACAACCATCCGGGAAGCTCT |
| >CRISPR_II_metagenome_CYPL031TF-VT-8 |
| TCGCTTTGATGGTTTATTTGTACTTGGTGTAGTAACT |
| >CRISPR_II_metagenome_CYPL031TF-VT-9 |
| ACCTGGGATCCGAACGCGAAGCCGGAGGACGGAGAGA |
| >CRISPR_II_metagenome_CYPL031TF-VT-10 |
| AGGAGTGTCTATGTACCTTAAGTTGGCTGGACGTAAT |
| >CRISPR_II_metagenome_CYPLF23TR-VT-1 |
| AGGAATAGTATGAACTAACTTTTCACCCAAACACCACCCCCTCCAC |
| >CRISPR_II_metagenome_CYPLF23TR-VT-2 |
| AGTATCGACTTGCATGTCGAATGCTACAATCGTGAACTCCTGTT |
| >CRISPR_II_metagenome_CYPLF23TR-VT-3 |
| GCCTTAGGAGGATTCTATGTCTACTGTCGTGTTCTACGGGG |
| >CRISPR_II_metagenome_CYPLF23TR-VT-4 |
| ATCTATTCAGGTCTCAGCTCTCCCCAGTCCTCAGGCAGCTCT |
| >CRISPR_II_metagenome_YMAA781TF-VT-1 |
| TTCGCTAGCGCAAGCGATAAGTTTCCCGTTTTTAACCGTGC |
| >CRISPR_II_metagenome_YMAA781TF-VT-2 |
| CTGGAACTACTACACCAGATACGGGCAGCGTTTTGTCAAAC |
| >CRISPR_II_metagenome_YMAA781TF-VT-3 |
| CCGTTGAAGGCAAACCTCCAACGGAGCATACACCCAGA |
| >CRISPR_II_metagenome_YMAA781TF-VT-4 |
| CTTTGGAAACTCCCCCTGCTGTGTTCCAGGGATGATCTGGATCCCCTGAA |
| >CRISPR_II_metagenome_YMAA781TF-VT-5 |
| CCGCTCCCGGCATTCCGAGAGCGGCAAAGGTTTTAGCAGT |
| >CRISPR_II_metagenome_YMAAC54TF-VT-1 |
| AAGATTTCTATTCGGCATTGATTGATCTGAAATCT |
| >CRISPR_II_metagenome_YMAAC54TF-VT-2 |
| TTGGCGAAAGCCATTCGAGGGGATTCACGGTTCAATGGC |
| >CRISPR_II_metagenome_YMAAC54TF-VT-3 |
| TTTCAGTCGGAATCTACCCAACGCGTTAAACGCTGGGT |
| >CRISPR_II_metagenome_YMAAC54TF-VT-4 |
| TCTCGGATTACTTAAACGCGGGGGAGGGCTTGAGTAATC |
| >CRISPR_II_metagenome_YMAAC54TF-VT-5 |
| TTGATAAACAGTACTATCTACCATTATGGGAATATCGT |
| >CRISPR_II_metagenome_YMAAC54TF-VT-6 |
| ATTTTGTTAATAAGAGACATAGTAGCCTCCAGGGTGGAT |
| >CRISPR_II_metagenome_YMAAC54TF-VT-7 |
| CTGGATACCGGAATGTAGAAGGAGAAGGCTACAAAA |
| >CRISPR_II_metagenome_YMAAC54TF-VT-8 |
| TTATTGGGAAGTCCCAAAAGAGCATCTCGGACACTACCG |
| >CRISPR_II_metagenome_YMAAC54TF-VT-9 |
| TACCGCGTAACCTGAAACCCCTCGTAAATAGTAGAGAATGGCG |
| >CRISPR_II_metagenome_YMAAV88TF-VT-1 |
| AACGATACCAAAGAGCAAATTCTGCGTTCCTCTCGGTGTG |
| >CRISPR_II_metagenome_YMAAV88TF-VT-2 |
| AACGAGTCCATAGAGTGAACTTGAACTTAGCGTTATCAG |
| >CRISPR_II_metagenome_YMAAV88TF-VT-3 |
| AACAATCAAATCTTCACACACTACCTTGACGACTAAGTTAG |
| >CRISPR_II_metagenome_YMAAV88TF-VT-4 |
| AACACCCAGAAGCACGTCGCGGAGCTCCAGCTTGATGGTCAG |
| >CRISPR_II_metagenome_YMAAV88TF-VT-5 |
| AACTGGGTGTTCTTTTACCCCTGTGAAGGGAGGTTGAC |
| >CRISPR_II_metagenome_YMAAV88TF-VT-6 |
| AACTTCATACGACCTCCCTTTCGGGATAAGAAACAACA |
| >CRISPR_II_metagenome_YMAAV88TF-VT-7 |
| AACTTAAGGGCGTTCAGTTCACTAAGAGGGGAATCCGAAT |
| >CRISPR_II_metagenome_YMAAV88TF-VT-8 |
| AACCAGTAAAGGGTTGGTCTTCTTAGTCCAAACGGGTTGG |
| >CRISPR_II_metagenome_YMAAZ74TR-VT-1 |
| TTCACGAAAAGAAGGAAAAAATTCTTCATAAATCCTGT |
| >CRISPR_II_metagenome_YMAAZ74TR-VT-2 |
| TTGTAACCTTCTACCTCACCTGCGATCTAGAGTAGTGTA |
| >CRISPR_II_metagenome_YMAAZ74TR-VT-3 |
| GGCCAGGGATCTTTCTCATCTTTGGCGTGCCACTGG |
| >CRISPR_II_metagenome_YMAAZ74TR-VT-4 |
| TTGGTGAATCTTACCCAGCGCTGTTGTTGGAAATTCCAATA |
| >CRISPR_II_metagenome_YMAAZ74TR-VT-5 |
| ACGGACTTTTGTCCATGCATCTTTGATAACTTGTTCGG |
| >CRISPR_II_metagenome_YMAAZ74TR-VT-6 |
| CTCTATCGTACATCTCAGTATTAGGTCCAAAAACAATT |
| >CRISPR_II_metagenome_YMAAZ74TR-VT-7 |
| AACAGAGAGCAGCTAGCCCCCCATTCCTGCTCTTGCGA |
| >CRISPR_II_metagenome_YMAAZ74TR-VT-8 |
| ACACTCGCGCAGATACCTGCGCTTGCTCGAATAGATGAT |
| >CRISPR_II_metagenome_YMAC324TF-VT-1 |
| TTTCATGCTGGGCTTTGCCCAGTGATGTGTTGTTTCGGAGGTC |
| >CRISPR_II_metagenome_YMAC324TF-VT-2 |
| CAACACTGGTCGCGCCCGCCTGACGAGCCGAGGTCGAAACCGAG |
| >CRISPR_II_metagenome_YMAC324TF-VT-3 |
| GTGGTTTGTAATAGGAGGCACTATGGCGACTATCAAGAT |
| >CRISPR_II_metagenome_YMAC324TF-VT-4 |
| TTTTTTACGAGTCGTTTTCAGGAGGCGGAAATGAAACTTCTT |
| >CRISPR_II_metagenome_YMAC333TR-VT-1 |
| AGAGCGTAAACGCGCTCGCTTCAATAAAGCTGTACGTA |
| >CRISPR_II_metagenome_YMAC333TR-VT-2 |
| GTGAGTTGCTGGATAACAACTAACCTCCCCCAAAAG |
| >CRISPR_II_metagenome_YMAC333TR-VT-3 |
| CAGAAGCCAGAATATACATTTATAACCCATTTTCTAAAA |
| >CRISPR_II_metagenome_YMAC333TR-VT-4 |
| TTGGAGGGGGCGAGCGCCCCGCTTGAAAGGATTACTCAAG |
| >CRISPR_II_metagenome_YMAC333TR-VT-5 |
| AGATTTATTACAGTGCCGGTCTGCACTGGTCCGAATTAGTCGG |
| >CRISPR_II_metagenome_YMAC333TR-VT-6 |
| AAACTCTATGAACCACCCAAGTTGGTGGTTCGCGTCTTCAA |
| >CRISPR_II_metagenome_YMAC333TR-VT-7 |
| TCTACTCCCAAAAGGGAGGAGGTAAATACAATGATCCTATGG |
| >CRISPR_II_metagenome_YMAC333TR-VT-8 |
| AGCTAGCTCTAGGTCTTACAGATAAACGAGTCTGTAGGACT |
| >CRISPR_II_metagenome_YMAC333TR-VT-9 |
| ACCCATACAGGAGTTAGCTAGATGATATTTACCCTGAT |
| >CRISPR_II_metagenome_YMAC333TR-VT-10 |
| ACGTCACCCAAATGGGAGGGGACTACGACGGGGACCGGA |
| >CRISPR_II_metagenome_YMAC970TR-VT-1 |
| TTTTCAATTTCCATTGGATTGCAATCCGCGATGGAGA |
| >CRISPR_II_metagenome_YMAC970TR-VT-2 |
| TTCCTATTGGATTAGCTTGAAAGACCCCGCAAGGAA |
| >CRISPR_II_metagenome_YMAC970TR-VT-3 |
| CGCCGACTACCCGAATGCCCGTCCGATTGCCCAATC |
| >CRISPR_II_metagenome_YMAC970TR-VT-4 |
| CGTCGGCCTCGGCCTGCGGGATCCGGGCCGGAGGTGCAAA |
| >CRISPR_II_metagenome_YMAC970TR-VT-5 |
| ACCCCAAAACACCCAGGAAAAATCCTGGGTGTTCTTTCT |
| >CRISPR_II_metagenome_YMAC970TR-VT-6 |
| TCAATTGGCTTAGTACCCCGACTTCTTCCAGATACCACCCGT |
| >CRISPR_II_metagenome_YMAC970TR-VT-7 |
| TTCCCGAAAGTGCTATACGGGAAGCTTGGGAGAAGGTCA |
| >CRISPR_II_metagenome_YMAC970TR-VT-8 |
| ATCATGGGAGCAGCATCTGGACTGCCCCGAAATCCCATCGG |
| >CRISPR_II_metagenome_YMAC970TR-VT-9 |
| TCGAAAATGCCCGCCCGAGAGGGGGGCTTGTTTACGGAC |
| >CRISPR_II_metagenome_YMAC970TR-VT-10 |
| GTCAAGATTAAGACCTCGCAAATCCAGGTGTGCGA |
| >CRISPR_II_metagenome_YMACA76TF-VT-1 |
| ACGGGTGGTATCTGGAAGAAGTCGGGGTACTAAGCCAATTGA |
| >CRISPR_II_metagenome_YMACA76TF-VT-2 |
| AGAAAGAACACCCAGGATTTTTCCTGGGTGTTTTGGGGT |
| >CRISPR_II_metagenome_YMACA76TF-VT-3 |
| TTTGCACCTCCGGCCCGGATCCCGCAGGCCGAGGCCGACG |
| >CRISPR_II_metagenome_YMACA76TF-VT-4 |
| GATTGGGCAATCGGACGGGCATTCGGGTAGTCGGCG |
| >CRISPR_II_metagenome_YMACA76TF-VT-5 |
| GATTGGGCAATCGGACGGGCATTCGGGTAGTCGGCG |
| >CRISPR_II_metagenome_YMACA76TF-VT-6 |
| TTCCTTGCGGGGTCTTTCAAGCTAATCCAATAGGAA |
| >CRISPR_II_metagenome_YMACA76TF-VT-7 |
| TCTCCATCGCGGATTGCAATCCAATGGAAATTGAAAA |
| >CRISPR_II_metagenome_YMBAM20TR-VT-1 |
| TTCTAGTATGTTAGCAGCTTCGTTCCAAGCTGCTTGAGC |
| >CRISPR_II_metagenome_YMBAM20TR-VT-2 |
| TTACTATTCATCTACCTCATCTGGTAGGAAGTAGCCGAAAC |
| >CRISPR_II_metagenome_YMBAM20TR-VT-3 |
| AAGGCACACATGTCCACGTAGTCCTTCAGGTTGTTAAA |
| >CRISPR_II_metagenome_YMBAM20TR-VT-4 |
| GATAATGCGGAAGACTTGGTAAACCTCCGTTATGTTCTGGTAA |
| >CRISPR_II_metagenome_YMBAM20TR-VT-5 |
| ACAATTGACGCAACAGACCTACAGCTGTGTTATACTG |
| >CRISPR_II_metagenome_YMBAM20TR-VT-6 |
| TGTGCTGCAAACCATAAGGCCCAATGGGCAACGGCTTGTACT |
| >CRISPR_II_metagenome_YMBAM20TR-VT-7 |
| AGTGTTGGTAAGATTTCCTGCGGCACCGGACGTTCAC |
| >CRISPR_II_metagenome_YMBAM20TR-VT-8 |
| TTTCTATATAACATTAAGTAATGGAAATACGTTATGCCGTA |
| >CRISPR_II_metagenome_YMBAM20TR-VT-9 |
| CTTTCCCAAGGATAATTGCAACGTCGCGAATGTATT |
| >CRISPR_II_metagenome_YMBAM20TR-VT-10 |
| AGTGTAGGCAAACAGGATATTTATGCTGGACAGGTAGG |
| >CRISPR_II_metagenome_YMBAM20TR-VT-11 |
| CTCTATACCCCTAGGCCTATGCCGTATAAAGTT |
| >CRISPR_II_metagenome_YMBAN46TR-VT-1 |
| ATCCGCATCTCTGGTGACCGCATCAAGGTACCTATCCTTGG |
| >CRISPR_II_metagenome_YMBAN46TR-VT-2 |
| GGGTCGATGGTCAGTACATCATCGCCATTCCGTATGGTG |
| >CRISPR_II_metagenome_YMBAN46TR-VT-3 |
| AATCTAGCAGGCGAATCACAACTAACCTGCTCTAAAA |
| >CRISPR_II_metagenome_YMBAN46TR-VT-4 |
| TAACTTGTGACCACTACAACCGAATCCACTATATGCT |
| >CRISPR_II_metagenome_YMBAN46TR-VT-5 |
| TCAAACGGTTTATTGATCAGATCTAATTCGCGCTCGACGC |
| >CRISPR_II_metagenome_YMBAN46TR-VT-6 |
| TACAAAGTTATCAATAGCAATACAGTTCATATCATCGAAA |
| >CRISPR_II_metagenome_YMBAN46TR-VT-7 |
| CAACACACCCAGGAGAAATCCTGGGTGTTGTTTTCATCC |
| >CRISPR_II_metagenome_YMBBN70TR-VT-1 |
| TTGAGGACGCGTGACTAAAGCAAGCCCGGGAGTTCTTAAACTCCCTGGGCGTTTCCAA |
| >CRISPR_II_metagenome_YMBBN70TR-VT-2 |
| GATTGTCTTTCACCACCCTGATGAGTGGTATGAATACTAGTTTCCAA |
| >CRISPR_II_metagenome_YMBBN70TR-VT-3 |
| AATGGACCCCTTTCGGTGTCCTTCTAAT |
| >CRISPR_II_metagenome_YMBBN70TR-VT-4 |
| CTCCCCCATTGAGATCCCCATCGTTAACTGCCCTCCCGAGTGTTTCCAA |
| >CRISPR_II_metagenome_YMBBN70TR-VT-5 |
| AATCCGAAGGATCTGTTATATTTCCTGGTTCCTGTCGTTTCCAA |
| >CRISPR_II_metagenome_YMBBN70TR-VT-6 |
| ACGGCGTCATCGAGTGGATCCCATCTTCTGGCCCGATCTGGGTTTCCAA |
| >CRISPR_II_metagenome_YMBBN70TR-VT-7 |
| TTATCTTCTACGATCTGGAGACAGTCGTAGAGCTACTGGGGTTTCCAA |
| >CRISPR_II_metagenome_YMBBN70TR-VT-8 |
| ATGAGTACCTAGTGTACAACTAAGTGTATCGTATACGTTTCCAA |
| >CRISPR_II_metagenome_YMBCR81TF-VT-1 |
| ATCACCCTGCTGCCCAAGGGATCCACATCCAGCAATGGGC |
| >CRISPR_II_metagenome_YMBCR81TF-VT-2 |
| GGATGGTGGACAAAGCCGCCTTCCCACTTGAGGGTAAACT |
| >CRISPR_II_metagenome_YMBCR81TF-VT-3 |
| CCCCGCTCCAGCAGACTGTCGCTGCAGTCTAACCAAAAC |
| >CRISPR_II_metagenome_YMBCR81TF-VT-4 |
| ATCCTCGCAACCCTGTCCATCGCCACTTGAACCTTCGCAATG |
| >CRISPR_II_metagenome_YMBD452TF-VT-1 |
| TTCATCAGAGCTATTTCTAGCAAACAAAAAGCAGACCTT |
| >CRISPR_II_metagenome_YMBD452TF-VT-2 |
| GGCCACCCGTCCTCCGTTGTGTGTTCCAACTTACAGTA |
| >CRISPR_II_metagenome_YMBD452TF-VT-3 |
| AATGAGATGGGGGCTGATCTAACTCATACCTTTGGTTTGGA |
| >CRISPR_II_metagenome_YMBD452TF-VT-4 |
| CACGATCAACCCATCGTGTGTCATCGACGGTATTCCCGTACT |
| >CRISPR_II_metagenome_YMBD452TF-VT-5 |
| GGACCGAATGCAGTGGTCTGCCAGAACCAAATACACTT |
| >CRISPR_II_metagenome_YMBD452TF-VT-6 |
| GAATGCGGTTATAGTGGTCGCAGGTTATCATGAGCCTGCT |
| >CRISPR_II_metagenome_YMBD452TF-VT-7 |
| GGTAAATACAAACCGTATACCTTCGGTTGTATACTCAA |
| >CRISPR_II_metagenome_YMBD452TF-VT-8 |
| AACGGTCTTTATTGGACCAAACCTGGACGCAATTTCAA |
| >CRISPR_II_metagenome_YMBEL14TR-VT-1 |
| TCACCCAAATGGGAGGACAGCCCACCCAGCCTGCTT |
| >CRISPR_II_metagenome_YMBEL14TR-VT-2 |
| GCGGTGCTCACAACCAAGTGCCCGTTTTTGACCGTCCCGAAG |
| >CRISPR_II_metagenome_YMBEL14TR-VT-3 |
| AGTAGAACTCCTAGTATTGTTGTCAAAAGATTCTGGAGT |
| >CRISPR_II_metagenome_YMBEL14TR-VT-4 |
| TCGAGTCTCCCTTCATAGCGCTTGAAGTAATCTCTG |
| >CRISPR_II_metagenome_YMBEL14TR-VT-5 |
| GCGCTGCTGCGGGCCCGTGCTGCTGCCGAAGGCTGGAAA |
| >CRISPR_II_metagenome_YMBEL14TR-VT-6 |
| TGGAACTACTACACCAGATACGGGCAGCGTTTTGTCAAAC |
| >CRISPR_II_metagenome_YMBEL14TR-VT-7 |
| CGTTGAAGGCAAACCTCCAACGGAGCATACACCCAGA |
| >CRISPR_II_metagenome_YMBEL14TR-VT-8 |
| TTTGGAAACTCCCCCTGCTGTGTTCCAGGGATGATCTGGATCCCCTGAA |
| >CRISPR_II_metagenome_YMBEL14TR-VT-9 |
| CGCTCCCGGCATTCCGAGAGCGGCAAAGGTTTTAGCAGT |
| >CRISPR_II_metagenome_YMBEQ85TF-VT-6 |
| CCTACCAGAAGTGGTAGGGATTGTTGAAGGAGAATAAAT |
| >CRISPR_II_metagenome_YMBEQ85TF-VT-7 |
| TCTGCAACAGGTCGTAGCGTGAATCCCGCAGGTCAGGTCCGT |
| >CRISPR_II_metagenome_YMBEQ85TF-VT-8 |
| TAAGCTCCTCATCCTCGTCTGTATCTTGGGGTTCGG |
| >CRISPR_II_metagenome_YMBEQ85TF-VT-9 |
| AACCACCAACTTGGGTGGTTCGATTACAGGCTTGGAGCCA |
| >CRISPR_II_metagenome_YMIA938TF-VT-1 |
| AAACCATTCGGGGTCGAGATAGTCTGATAACTTTATCA |
| >CRISPR_II_metagenome_YMIA938TF-VT-2 |
| ATTACTTTACCTGCTTGAACGTCACTTTGCGTGACG |
| >CRISPR_II_metagenome_YMIA938TF-VT-3 |
| TGGGTTGAGTACATGGTTACAACGAATTACGATCTAATCTAGTCCC |
| >CRISPR_II_metagenome_YMIA938TF-VT-4 |
| ACTAAGGGTCCACTCTGGTCCGGTATGGCACTGGT |
| >CRISPR_II_metagenome_YMIA938TF-VT-5 |
| ACTAAGGGTCCACTCTGGTCCGGTATGGCACTGGT |
| >CRISPR_II_metagenome_YMIA938TF-VT-6 |
| CTTTTCCAAAGAGCACCGAAAAGCTGGTGGAGAACATGTTCTC |
| >CRISPR_II_metagenome_YMIA938TF-VT-7 |
| ATCTAATTAACAACGATGCTATGGTAGGTTTTGCAGGT |
| >CRISPR_II_metagenome_YMIA938TF-VT-8 |
| CGGACTTCGACGAGTATATTCGGGACTGCACAATACA |
| >CRISPR_II_metagenome_YMIA938TF-VT-9 |
| TTTTCATCCCAGTGACCAAGTAGTCTAACCTCCCCCTCAAG |
| >CRISPR_II_metagenome_YMIB088TR-VT-1 |
| AGGTGATCACGGCGCCCTCCGGGACGTCGCCGCCATCCACGACG |
| >CRISPR_II_metagenome_YMIB088TR-VT-2 |
| CAACTTACGCAGGAAAGCGATGTTATGCATAGTAAA |
| >CRISPR_II_metagenome_YMIB088TR-VT-3 |
| CCCCAAACCTGGGGTTACCCCAGATTAAATATTT |
| >CRISPR_II_metagenome_YMIB088TR-VT-4 |
| GCTGCTATCAGGCGTAACCTTGAAGCCTTTATGCCCCACTC |
| >CRISPR_II_metagenome_YMIB088TR-VT-5 |
| TGATAGAGATCATCGGAGTGCCTCCATTGACTACACCCC |
| >CRISPR_II_metagenome_YMIB088TR-VT-6 |
| CTATAGCCACAAAAAATCCCCCGCCAATAAGGCGGG |
| >CRISPR_II_metagenome_YMIB386TR-VT-1 |
| AAGCCTTGTTGAAGGCTCTCCCCAAAGAACTCGGCAAAATTGAGGA |
| >CRISPR_II_metagenome_YMIB386TR-VT-2 |
| GGGCACTTTTGACCCTTACTTTTGGGGCCGGCACACCCCCT |
| >CRISPR_II_metagenome_YMIB386TR-VT-3 |
| AGCCTTTGTTGAGATTAGTCAGCTAGTTGGTTTAGTTGTCGA |
| >CRISPR_II_metagenome_YMIB386TR-VT-4 |
| CACCCACGGTCCGAATAGTGTAATAAACATTGTAAA |
| >CRISPR_II_metagenome_YMIB386TR-VT-5 |
| GTTTTCATTAGAGAAGAAGAGGAGTAAGTAACTAGTA |
| >CRISPR_II_metagenome_YMIB386TR-VT-6 |
| TGCAAGTCAAATTGAGGCCCAGGGACAAAGCCTGAA |
| >CRISPR_II_metagenome_YMIB386TR-VT-7 |
| ATATACTCGCAACGAGCGCTCCTTAGAATCTGCTCTTTAGT |
| >CRISPR_II_metagenome_YMIB386TR-VT-8 |
| ATCCCCGATATATTCGATGCCTTCTCCTACGTAGTTT |
| >CRISPR_II_metagenome_YMIB557TR-VT-1 |
| CTCCGACTAATTCGGACCAGTGCAGACCGGCACTGTA |
| >CRISPR_II_metagenome_YMIB557TR-VT-2 |
| TGTCTTCAGTGATTTGTGCTGGCTTGCCGGAGGGAGTCGCG |
| >CRISPR_II_metagenome_YMIB557TR-VT-3 |
| AACAGCAGCAAGCGCAGGAGAAACTGGGGTTTGGGCC |
| >CRISPR_II_metagenome_YMIB557TR-VT-4 |
| GTTTTCGGGAACATCGTCAGCTTCTACGATTAACTTCCCGT |
| >CRISPR_II_metagenome_YMIB557TR-VT-5 |
| GACTTATAGTGAGCAATGTTGAATTTCTCACGGTCTT |
| >CRISPR_II_metagenome_YMIB557TR-VT-6 |
| ATCACCTTCTTTTTCTGACAGTGATTTCTCTAGCTTAGCTATG |
| >CRISPR_II_metagenome_YMIB557TR-VT-7 |
| CCTATAGCCACAAAAAATCCCCCGCCAATAAGGCGGG |
| >CRISPR_II_metagenome_YMJAT64TF-VT-1 |
| AACGATATCCCCCTGACCCCCTTACCCCCGCTTTCCCATCGT |
| >CRISPR_II_metagenome_YMJAT64TF-VT-2 |
| AACTTCTCCAGCCGCTTATTCTGGAACCACGTAACTCCATT |
| >CRISPR_II_metagenome_YMJAT64TF-VT-3 |
| AACGCCCAACAGCTCTTCCTTGATAAATCCTTCTCTAATAG |
| >CRISPR_II_metagenome_YMJAU14TF-VT-1 |
| GCCATTTACGAGCGGGTGGTCTACTTCAGACCAATCGAG |
| >CRISPR_II_metagenome_YMJAU14TF-VT-2 |
| GGGATATCCTCAAACAAGACCTAGACGAAGCGTATGAGAA |
| >CRISPR_II_metagenome_YMJAU14TF-VT-3 |
| AGGAATGGTTGATTGATTAAATAAAAAAGAGGGGTTTAAG |
| >CRISPR_II_metagenome_YMJAU14TF-VT-4 |
| ATGACTGGCATCAGCCAGATAGCTGGCGGGTGCCAGATGCTAT |
| >CRISPR_II_metagenome_YMJAU14TF-VT-5 |
| AAGAATTCTTCGACGCCACCTTACTCGAACCGGAGG |
| >CRISPR_II_metagenome_YMJAU14TF-VT-6 |
| TATAACACCATAGCGGAGGTATATGAGGTTTTCCA |
| >CRISPR_II_metagenome_YMJAU14TF-VT-7 |
| CATGAAAGTAGTCGCGGCGTTTAACTAGTCCAATCCCAAGTC |
| >CRISPR_II_metagenome_YMBDR75TR-VT-1 |
| GGTTAACCCAACTGGGGTGAAGTTGTAGAGGATAACTTTG |
| >CRISPR_II_metagenome_YMBDR75TR-VT-2 |
| GTCAAAACAGGTTCCATAACAACCTCCCTTTACGGAGT |
| >CRISPR_II_metagenome_YMBDR75TR-VT-3 |
| ACTCTCTTCGAGGACCAACCAGCGATTCTTATAAGGAATC |
| >CRISPR_II_metagenome_YMBDR75TR-VT-4 |
| TCATAACTAACCTCCAAAGGAGTAAAGTTTTCAAAAATAAAA |
| >CRISPR_II_metagenome_YMBDR75TR-VT-5 |
| ACAGTTTTCTTCTACAGAGGAAACTGGGTGATCTTCCAAGAC |
| >CRISPR_II_metagenome_YMBDR75TR-VT-6 |
| TTTTTCAGTGAAGTTCGGGACGTATTGGTAGCGAA |
| >CRISPR_II_metagenome_YMBDR75TR-VT-7 |
| TGGAAGAAAAGCGCGGGAACACCGTGGTCCAATACCAATA |
| >CRISPR_II_metagenome_YMBDR75TR-VT-8 |
| CAGAGGAAAACAAAGCTTATATGGATCAAAAAAAAGAA |
| >CRISPR_II_metagenome_YMBDR75TR-VT-9 |
| AAGAAAGTTAATTCAAATTATTCCTCCCGCTGAGCC |
| >CRISPR_II_metagenome_YMBDR75TR-VT-10 |
| CCTCAAAGAACAGCGGAAGCAGATGGAAGCCGCTCTGAA |
| >CRISPR_II_metagenome_CYOAP27TR-VT-11 |
| CTCGGGACAGAGATCTTCGGGGTTTACTGAGACAACA |
| >CRISPR_II_metagenome_CYOAP27TR-VT-12 |
| TAACCGCAGCCATTGACGGGCTGCTTCCACCTGCT |
|  |
|  |
| >CRISPR_III_A_OS-A-VT-1 |
| GGACGGATCCCTTGCCGGGTCAGGGCCCTCCCTT |
| >CRISPR_III_A_OS-A-VT-2 |
| TGAAGTCCTGCCGTCCCGCGGGCCGGGCCGACTT |
| >CRISPR_III_A_OS-A-VT-3 |
| GTACGCTCCCACGATCTCCAAGCCTGCCGCCTTCG |
| >CRISPR_III_A_OS-A-VT-4 |
| TCCAGTGCCACCGCTATTGCGGGGACTAGCCAGGT |
| >CRISPR_III_A_OS-A-VT-5 |
| GACTCTTTGTCCGTCACTCCAACGTAGAGGATTA |
| >CRISPR_III_A_OS-A-VT-6 |
| GCTATTCGGTGGGCTTCCCGCTCAAGTTGTGCTTC |
| >CRISPR_III_A_OS-A-VT-7 |
| CCGGTGAACTCCCGCAGCTTGGGCGCCCACTTGTT |
| >CRISPR_III_metagenome_CYMA202TF-VT-1 |
| GTACAGCAGGAGAACTACCAGGGGGCTCATAGATT |
| >CRISPR_III_metagenome_CYMA202TF-VT-2 |
| TCTCAGCACTGGAGATTTGTGAGCAGATTGAGCGC |
| >CRISPR_III_metagenome_CYMA202TF-VT-3 |
| GCTCTGCTGGCGGCTCATCTGGCGCGTCCGGCGGC |
| >CRISPR_III_metagenome_CYMA202TF-VT-4 |
| GGGTTGTTCACTTCCATCGTGAATCTCTTACTGGC |
| >CRISPR_III_metagenome_CYMA202TF-VT-5 |
| CCAAACTGGAGCCGGCGGGGCGCACCAGCACCACC |
| >CRISPR_III_metagenome_CYMA202TF-VT-6 |
| GTGATTCCAGTGTCGATTACTGGTCGGATTAGCAGC |
| >CRISPR_III_metagenome_CYMA202TF-VT-7 |
| GGGCAGAGGTCTTTCGAGACGCGCGTTTGGTAGGGC |
| >CRISPR_III_metagenome_CYMA202TF-VT-8 |
| TCTCCGGGGAATAGGTGAACCTTGGCGCAACGCCT |
| >CRISPR_III_metagenome_CYMA202TF-VT-9 |
| TCTCCGGGGAATAGGTGAACCTTGGCGCAACGCCT |
| >CRISPR_III_metagenome_CYMA202TF-VT-10 |
| TCGGCAAATGACGCACCAGCTGGCTGTATTTGTC |
| >CRISPR_III_metagenome_YMJAN92TF-VT-1 |
| GTACAGCAGGAGAACTACCAGGGGGCTCATAGATT |
| >CRISPR_III_metagenome_YMJAN92TF-VT-2 |
| TCTCAGCACTGGAGATTTGTGAGCAGATTGAGCGC |
| >CRISPR_III_metagenome_YMJAN92TF-VT-3 |
| GCTCTGCTGGCGGCTCATCTGGCGCGTCCGGCGGC |
| >CRISPR_III_metagenome_YMJAN92TF-VT-4 |
| GGGTTGTTCACTTCCATCGTGAATCTCTTACTGGC |
| >CRISPR_III_metagenome_YMJAN92TF-VT-5 |
| CCAAACTGGAGCCGGCGGGGCGCACCAGCACCACC |
| >CRISPR_III_metagenome_YMJAN92TF-VT-6 |
| TGCCCAACCTGGCCGACGCGGTGCGACGGGTGGCC |
| >CRISPR_III_metagenome_YMJAN92TF-VT-7 |
| TCTCCGGGGAATAGGTGAACCTTGGCGCAACGCCT |
| >CRISPR_III_metagenome_YMJAN92TF-VT-8 |
| TCGGCAAATGACGCACCAGCTGGCTGTATTTGTC |
| >CRISPR_III_metagenome_CYPKN21TF-VT-1 |
| GGGATCCCTCTAACCCCAGAGAAGACTGCCATG |
| >CRISPR_III_metagenome_CYPKN21TF-VT-2 |
| TGCCAGCCGCCACCCAAGACGGGCCATGCTAGA |
| >CRISPR_III_metagenome_CYPKN21TF-VT-3 |
| GTTCACCCCGCCCGGACGCTGCTCTGCCTTCGA |
| >CRISPR_III_metagenome_CYPKN21TF-VT-4 |
| TAGCTATCCCATTGCTCCTCCGATACTGTGAGA |
| >CRISPR_III_metagenome_CYPKN21TF-VT-5 |
| GGAAAATCGGCTTACAGAAGAAGAACTGGAAGC |
| >CRISPR_III_metagenome_CYPKN21TF-VT-6 |
| GCAGGGGCCGTTGGAAGGGCCGAGAAGGCTGCT |
| >CRISPR_III_metagenome_CYPKN21TF-VT-7 |
| GATATAGGCTGAGACTCTAAGGAAACTTTCTACC |
| >CRISPR_III_metagenome_CYPKN21TF-VT-8 |
| TCAGCTGGGGATCCCAGTCATCCCCACGCCGTT |
| >CRISPR_III_metagenome_CYPKN21TF-VT-9 |
| GTCTCGTCGACAACCGAGTATACTGACGTAGAC |
| >CRISPR_III_metagenome_CYPKN21TF-VT-10 |
| GCGGGCTAAAGGTGCTACTCAGGACGCAGATACA |
| >CRISPR_III_metagenome_CYPKN21TF-VT-11 |
| CCCCATCCTCTGCTTCTAGCGTCAGCCCTTGCA |
| >CRISPR_III_metagenome_CYPKN21TF-VT-12 |
| GGCGTTGAGCACGAAAACTTGCCCACCTTCTGGG |
| >CRISPR_III_metagenome_GYUA948TR-VT-1 |
| GGTCCAGGTTTCCCTCGACTCCCGTGAGTGGT |
| >CRISPR_III_metagenome_GYUA948TR-VT-2 |
| ATCAACGACATCATGAATGCTGCCGCGGCTGT |
| >CRISPR_III_metagenome_GYUA948TR-VT-3 |
| GCAGCAGAGACCTGATCCAGCCAGGAGATGAT |
| >CRISPR_III_metagenome_GYUA948TR-VT-4 |
| TTTTTCCGCGACCGCCCGCTGCCGACGATTGA |
| >CRISPR_III_metagenome_GYUA948TR-VT-5 |
| AGCGCTGCCGACGCCGGAACGTCGCTAAAGAC |
| >CRISPR_III_metagenome_GYUA948TR-VT-6 |
| ACGAGACGGAAGCGGCGTCGTTGACGGAAGCG |
| >CRISPR_III_metagenome_GYUA948TR-VT-7 |
| GTGGTCGCGCGGAGCGGATTTGATACCTACGT |
| >CRISPR_III_metagenome_GYUA948TR-VT-8 |
| AGATCGCCGGGTCGGAACTGTACGCGATCAAT |
| >CRISPR_III_metagenome_GYUA948TR-VT-9 |
| AACAGAAACCGCCAGGCGTGCGCCCGGCGGTT |
| >CRISPR_III_metagenome_GYUA948TR-VT-10 |
| CTCGTGCACGCGCGCGCAACGCTCAGCAGCAA |
| >CRISPR_III_metagenome_GYUA948TR-VT-11 |
| CGGTGACCATAGCGACGTAAGGCTCGGCGTCG |
| >CRISPR_III_metagenome_CYNAC89TF-VT-1 |
| TGCGGTCGTCGATAGGAATGAACTGCTCGTAGAA |
| >CRISPR_III_metagenome_CYNAC89TF-VT-2 |
| GTTTGAGGACAATCTGGGGCTTTGCTCTGTTCC |
| >CRISPR_III_metagenome_CYNAC89TF-VT-3 |
| GCGAATTTGGGCTGGGCGTGCCAGTGCGGATGCT |
| >CRISPR_III_metagenome_CYNAC89TF-VT-4 |
| CAACCTCGCAATTGCTTGCAGTAAGCCGACTTTT |
| >CRISPR_III_metagenome_CYNAC89TF-VT-5 |
| TTAGCCAGTCGTCTCAAGGAGCACGGCTGGCCC |
| >CRISPR_III_metagenome_CYNAC89TF-VT-6 |
| TCACGGTTGACGCCGCCGTGGCCACTATCGGGGC |
