# Supplementary material for: Germ Warfare in a Microbial Mat Community: CRISPRs Provide Insights into the Co-Evolution of Host and Viral Genomes
Source: PLoS One. 2009 Jan 9;4(1):e4169. doi: 10.1371/journal.pone.0004169 (PMC2612747; doi:10.1371/journal.pone.0004169)
Supplement: Table S6 — Summary of sample sites, number of CRISPR containing sequences and total number of sequences in the metagenome datasets. (0.04 MB DOC) [file pone.0004169.s006.doc]

**Table S5 Metagenome datasets. From {Bhaya, 2007 #27}. Summary of sample sites, number of CRISPR containing sequences and total number of sequences in the metagenome datasets.**

| **Sample Site** | **Number of sequences with a CRISPR** | **Number of sequences** | **Comment** |
| --- | --- | --- | --- |
| **Octopus Spring Total** |  | **106,870** |  |
| Octopus Spring High Temperature | 6 | 8,054 | Top 1mm of core, taken from area of Octopus Spring; temperature ranged from 58-67°C. Nov 5, 2004 |
| Octopus Spring Low Temperature | 125 | 98,816 | Top 1mm of core, taken from area of Octopus Spring; temperature ranged from 53.5-63.4°C. Nov 5, 2004 |
| **Mushroom Spring Total** |  | **67,181** |  |
| Mushroom Spring Low Temperature | 17 | 16,692 | Top 1mm of core taken from area of Mushroom Spring where temperature was 60°C. Oct 2, 2003 |
| Mushroom Spring High Temperature | 39 | 50,489 | Top 1mm slice of core taken from area of Mushroom Spring where temperature was 65°C. Oct 2, 2003 |
