# Supplementary material for: Germ Warfare in a Microbial Mat Community: CRISPRs Provide Insights into the Co-Evolution of Host and Viral Genomes
Source: PLoS One. 2009 Jan 9;4(1):e4169. doi: 10.1371/journal.pone.0004169 (PMC2612747; doi:10.1371/journal.pone.0004169)
Supplement: Supplementary file 6 [file pone.0004169.s005.doc]

## Sheet: No-Roseo

Genome or Metagenome	Spring	Temperature	Repeat	Make up	Shared Spacers	Shared Spacers	Shared Spacers	Shared Spacers	Shared Spacers	Shared Spacers
Meta-only	Both	Both	Mix	Mix	CRISPR_IA unmapped_YMIAV82TR-SP-4	CRISPR_IIF OS-B'_CYPAO83TF-SP-3	CRISPR_IIB OS-A_YMBAN46TR-SP-7	CRISPR_IID OS-B'_YMJAO09TR-SP-3	CRISPR_IIE OS-A_YMAC970TR-SP-5	CRISPR_IIE OS-A_YMACA76TF-SP-2
Both	Both	High-Only	CRISPR_2	OS-A	CRISPR_IIB OS-A_Genome-SP-6	CRISPR_IIB OS-A_GYRAT76TR-SP-3	CRISPR_IIB OS-A_YMAAA56TR-SP-1	CRISPR_IIB OS-A_YMABY66TF-SP-5	CRISPR_IIB OS-A_YMBB565TF-SP-2	CRISPR_IIB OS-A_YMBBN70TR-SP-1
Both	Both	Low-Only	CRISPR_2	OS-B	CRISPR_IIA OS-B'_Genome-SP-1	CRISPR_IIA OS-B'_CYPDL59TR-SP-9	CRISPR_IIA OS-B'_CYPFC31TF-SP-1	CRISPR_IIA OS-B'_CYOB470TF-SP-1	CRISPR_IIA OS-B'_YMIB088TR-SP-6	CRISPR_IIA OS-B'_YMIB557TR-SP-7
Both	OS-Only	Both	CRISPR_1	Mix	CRISPR_IA OS-A_Genome-SP-1	CRISPR_IA OS-B'_Genome-SP-1	CRISPR_IA unmapped_CYPGU34TR-SP-1	CRISPR_IA OS-B'_CYPLL02TR-SP-7	CRISPR_IA OS-B'_CYPF247TF-SP-1
Meta-only	OS-Only	Low-Only	CRISPR_2	Unknown	CRISPR_II unmapped_CYOAB48TR-SP-2	CRISPR_IIA OS-A_CYOAB48TR-SP-3	CRISPR_IID OS-B'_CYPHA03TR-SP-1	CRISPR_IID OS-B'_CYPHA03TR-SP-2	CRISPR_IID OS-B'_CYPHA03TR-SP-3
Meta-only	OS-Only	Low-Only	CRISPR_2	Unknown	CRISPR_II unmapped_CYOAB48TR-SP-7	CRISPR_II unmapped_CYOAB48TR-SP-8        	CRISPR_II unmapped_CYOAB48TR-SP-9	CRISPR_IID OS-B'_CYPE216TR-SP-3
Meta-only	Both	Both	CRISPR_2	OS-A	CRISPR_IIE OS-A_CYPDV63TR-SP-2	CRISPR_IIE OS-A_YMAC970TR-SP-3	CRISPR_IIE OS-A_YMACA76TF-SP-4	CRISPR_IIE OS-A_YMACA76TF-SP-5
Meta-only	Both	Both	CRISPR_2	OS-A	CRISPR_IIC OS-A_CYOBD48TF-SP-1	CRISPR_IIC OS-A_CYPB048TR-SP-1	CRISPR_IIC OS-A_YMAC324TF-SP-1	CRISPR_IIC OS-A_YMBEE53TR-SP-1
Both	Both	High-Only	CRISPR_2	OS-A	CRISPR_IIA OS-A_Genome-SP-2	CRISPR_IIA OS-A_YMAA781TF-SP-4	CRISPR_IIA OS-A_YMBDT21TF-SP-1	CRISPR_IIA OS-A_YMBEL14TR-SP-8
Both	OS-Only	Both	CRISPR_1	Mix	CRISPR_IB OS-A_Genome_OS-A-SP-5	CRISPR_IA OS-B'_CYPJW61TR-SP-4          	CRISPR_IA unmapped_CYPGU34TR-SP-3
Meta-only	OS-Only	Low-Only	CRISPR_1	OS-B	CRISPR_IB OS-B'_CYPA321TF-SP-2	CRISPR_IB OS-B'_CYPA321TF-SP-3	CRISPR_IB OS-B'_CYPHE06TR-SP-9
Meta-only	Both	Both	Mix	Mix	CRISPR_IB OS-A_YMBDU19TF-SP-1	CRISPR_IIG OS-B'_CYPAW81TR-SP-8	CRISPR_IIE OS-A_CYNA391TR-SP-4
Meta-only	Both	Both	CRISPR_2	Mix	CRISPR_IIB OS-A_YMBD452TF-SP-5	CRISPR_IIA OS-B'_CYOB470TF-SP-8	CRISPR_II unmapped_CYPE874TF-SP-1
Meta-only	OS-Only	Low-Only	CRISPR_2	OS-B	CRISPR_IID OS-B'_CYPEG04TR-SP-6	CRISPR_IID OS-B'_CYPEG04TR-SP-7	CRISPR_IID OS-B'_CYPEG04TR-SP-8
Meta-only	OS-Only	Low-Only	Mix	OS-B	CRISPR_IA OS-B'_CYPCF80TF-SP-3	CRISPR_IA OS-B'_CYPCF80TF-SP-2	CRISPR_IIF OS-B'_CYPK565TR-SP-7
Meta-only	Both	Low-Only	CRISPR_2	Mix	CRISPR_IIE OS-A_CYPDW52TF-SP-6	CRISPR_IB OS-B'_YMJA680TF-SP-8	CRISPR_IIE OS-A_Genome-SP-11
Meta-only	Both	Both	CRISPR_2	OS-A	CRISPR_IIE OS-A_CYPDV63TR-SP-5	CRISPR_IIE OS-A_YMAC970TR-SP-1	CRISPR_IIE OS-A_YMACA76TF-SP-7
Both	Both	High-Only	CRISPR_2	OS-A	CRISPR_IIA OS-A_Genome-SP-1	CRISPR_IIA OS-A_YMAA781TF-SP-5	CRISPR_IIA OS-A_YMBEL14TR-SP-9
Both	Both	High-Only	CRISPR_2	OS-A	CRISPR_IIE OS-A_Genome-SP-1	CRISPR_IIE OS-A_YMBBC81TR-SP-2	CRISPR_IIE OS-A_YMJAT64TF-SP-2
Meta-only	Both	Both	CRISPR_3	OS-A	CRISPR_IIIA OS-A_CYMA202TF-SP-8	CRISPR_IIIA OS-A_CYMA202TF-SP-9	CRISPR_IIIA OS-A_YMJAN92TF-SP-7
Meta-only	Both	Low-Only	CRISPR_2	Mix	CRISPR_IID OS-A_YMIAI61TR-SP-3	CRISPR_IIF OS-B'_CYOCJ70TR-SP-3
Meta-only	OS-Only	Low-Only	Mix	Mix	CRISPR_IA OS-A_CYPEU56TF-SP-1	CRISPR_IIG OS-B'_CYPAW81TR-SP-3
Meta-only	OS-Only	Low-Only	Mix	Mix	CRISPR_IB OS-B'_CYPBW78TR-SP-2	CRISPR_IIC OS-A_CYOBD48TR-SP-1
Meta-only	OS-Only	Low-Only	CRISPR_1	Mix	CRISPR_IA OS-A_CYOCM11TF-SP-7	CRISPR_IA OS-B'_CYPM809TR-SP-2
Meta-only	OS-Only	Low-Only	CRISPR_1	Mix	CRISPR_IA OS-A_CYPC933TR-SP-1	CRISPR_IA OS-B'_CYPF247TF-SP-2
Meta-only	OS-Only	Low-Only	CRISPR_1	Mix	CRISPR_IB OS-B'_CYPA321TF-SP-7	CRISPR_IA OS-A_CYPC933TR-SP-2
Meta-only	Both	Both	Mix	Mix	CRISPR_IB OS-B'_CYPH825TF-SP-3	CRISPR_IIC OS-A_YMAAP30TF-SP-5
Meta-only	Both	Both	CRISPR_2	Mix	CRISPR_IID OS-A_YMBDR75TR-SP-2	CRISPR_IIG OS-B'_CYPJ361TR-SP-2
Meta-only	Both	Both	CRISPR_2	Mix	CRISPR_IID OS-A_YMBDR75TR-SP-9	CRISPR_IIG OS-B'_CYOBC31TR-SP-4
Meta-only	MS-Only	Both	CRISPR_2	Mix	CRISPR_IIA OS-B'_YMIB557TR-SP-1	CRISPR_IIB OS-A_YMAC333TR-SP-5
Meta-only	MS-Only	Both	CRISPR_2	Mix	CRISPR_IIA OS-B'_YMIB088TR-SP-3	CRISPR_IIC OS-A_YMAAP30TF-SP-8
Both	OS-Only	Low-Only	CRISPR_1	Mix	CRISPR_IB OS-B'_Genome-SP-4	CRISPR_IA OS-B'_CYPF150TR-SP-5  
Both	OS-Only	Both	Mix	Mix	CRISPR_IA OS-A_Genome-SP-25	CRISPR_IIG OS-B'_CYPK760TF-SP-2
Both	OS-Only	Both	Mix	Mix	CRISPR_IA OS-A_Genome-SP-40	CRISPR_IIF OS-B'_CYPDM04TF-SP-1
Both	OS-Only	Both	Mix	Mix	CRISPR_IA OS-A_Genome-SP-26	CRISPR_IID OS-B'_CYPBU82TR-SP-1
Both	OS-Only	Both	CRISPR_2	Mix	CRISPR_IA OS-A_Genome-SP-33	CRISPR_IIF OS-B'_CYOCJ70TF-SP-3
Meta-only	MS-Only	Low-Only	CRISPR_2	OS-A	CRISPR_IIB OS-A_YMIA938TF-SP-5	CRISPR_IIB OS-A_YMIA938TF-SP-4
Meta-only	OS-Only	Low-Only	CRISPR_2	OS-A	CRISPR_IIC OS-A_CYPEU81TR-SP-7	CRISPR_IIC OS-A_CYPE865TR-SP-2
Meta-only	OS-Only	Low-Only	CRISPR_2	OS-A	CRISPR_IIC OS-A_CYPEU81TR-SP-6	CRISPR_IIC OS-A_CYPE865TR-SP-1
Meta-only	OS-Only	Low-Only	CRISPR_1	Mix	CRISPR_IA OS-A_CYOCM11TF-SP-8	CRISPR_IB OS-B'_CYPBV46TF-SP-5  
Meta-only	Both	High-Only	CRISPR_2	OS-A	CRISPR_IIE OS-A_YMAC970TR-SP-8	CRISPR_IIE OS-A_CYNA391TR-SP-9
Meta-only	Both	High-Only	CRISPR_2	OS-A	CRISPR_IIE OS-A_YMAC970TR-SP-9	CRISPR_IIE OS-A_CYNA391TR-SP-8
Meta-only	Both	High-Only	CRISPR_2	OS-A	CRISPR_IIE OS-A_YMAC970TR-SP-10	CRISPR_IIE OS-A_CYNA391TR-SP-7
Meta-only	Both	High-Only	CRISPR_1	OS-A	CRISPR_IA unmapped_YMAAA02TF-SP-1	CRISPR_IA unmapped_YMAAA02TF-SP-3
Meta-only	MS-Only	High-Only	CRISPR_2	OS-A	CRISPR_IIE OS-A_YMACA76TF-SP-1	CRISPR_IIE OS-A_YMAC970TR-SP-6
Meta-only	MS-Only	High-Only	CRISPR_2	OS-A	CRISPR_IIE OS-A_YMACA76TF-SP-3	CRISPR_IIE OS-A_YMAC970TR-SP-4
Meta-only	MS-Only	High-Only	CRISPR_2	OS-A	CRISPR_IIE OS-A_YMACA76TF-SP-6	CRISPR_IIE OS-A_YMAC970TR-SP-2
Meta-only	MS-Only	High-Only	CRISPR_2	OS-A	CRISPR_IIB OS-A_YMBBN70TR-SP-2	CRISPR_IIB OS-A_YMABY66TF-SP-4
Meta-only	MS-Only	High-Only	CRISPR_2	OS-A	CRISPR_IIB OS-A_YMBBN70TR-SP-3	CRISPR_IIB OS-A_YMABY66TF-SP-3
Meta-only	MS-Only	High-Only	CRISPR_2	OS-A	CRISPR_IIB OS-A_YMBBN70TR-SP-4	CRISPR_IIB OS-A_YMABY66TF-SP-2
Meta-only	MS-Only	High-Only	CRISPR_2	OS-A	CRISPR_IIB OS-A_YMBBN70TR-SP-5	CRISPR_IIB OS-A_YMABY66TF-SP-1
Meta-only	MS-Only	High-Only	CRISPR_2	OS-A	CRISPR_IIC OS-A_YMABI04TR-SP-4	CRISPR_IIC OS-A_YMABI04TR-SP-2
Meta-only	MS-Only	High-Only	CRISPR_2	OS-A	CRISPR_IIC OS-A_YMABI04TR-SP-3	CRISPR_IIC OS-A_YMABI04TR-SP-1
Meta-only	MS-Only	High-Only	CRISPR_2	OS-A	CRISPR_IIA OS-A_YMBEL14TR-SP-7	CRISPR_IIA OS-A_YMAA781TF-SP-3
Meta-only	MS-Only	High-Only	CRISPR_2	OS-A	CRISPR_IIA OS-A_YMBEL14TR-SP-6	CRISPR_IIA OS-A_YMAA781TF-SP-2
Meta-only	Both	Both	CRISPR_3	OS-A	CRISPR_IIIA OS-A_CYMA202TF-SP-10	CRISPR_IIIA OS-A_YMJAN92TF-SP-8
Meta-only	Both	Both	CRISPR_3	OS-A	CRISPR_IIIA OS-A_CYMA202TF-SP-5	CRISPR_IIIA OS-A_YMJAN92TF-SP-5
Meta-only	Both	Both	CRISPR_3	OS-A	CRISPR_IIIA OS-A_CYMA202TF-SP-4	CRISPR_IIIA OS-A_YMJAN92TF-SP-4
Meta-only	Both	Both	CRISPR_3	OS-A	CRISPR_IIIA OS-A_CYMA202TF-SP-3	CRISPR_IIIA OS-A_YMJAN92TF-SP-3
Meta-only	Both	Both	CRISPR_3	OS-A	CRISPR_IIIA OS-A_CYMA202TF-SP-2	CRISPR_IIIA OS-A_YMJAN92TF-SP-2
Meta-only	Both	Both	CRISPR_3	OS-A	CRISPR_IIIA OS-A_YMJAN92TF-SP-1	CRISPR_IIIA OS-A_CYMA202TF-SP-1                
Meta-only	Both	Both	CRISPR_2	OS-A	CRISPR_IIC OS-A_YMACD96TF-SP-4	CRISPR_IIC OS-A_CYPB357TR-SP-1
Meta-only	Both	Both	CRISPR_1	OS-A	CRISPR_IB OS-A_YMBCA60TF-SP-7	CRISPR_IA OS-A_CYPEU56TF-SP-2
Meta-only	MS-Only	Both	CRISPR_2	OS-A	CRISPR_IIE OS-A_YMJAT64TF-SP-3	CRISPR_IIE OS-A_YMBBC81TR-SP-3
Genome	OS-Only	High-Only	CRISPR_1	OS-A	CRISPR_IB OS-A_Genome-SP-7	CRISPR_IB OS-A_Genome-SP-8
Both	Both	High-Only	CRISPR_2	OS-A	CRISPR_IA OS-A_Genome-SP-24	CRISPR_IIA OS-A_YMBDT21TF-SP-8
Both	Both	High-Only	CRISPR_2	OS-A	CRISPR_IIB OS-A_Genome-SP-5	CRISPR_IIB OS-A_YMBB565TF-SP-1
Both	Both	High-Only	CRISPR_1	OS-A	CRISPR_IA OS-A_Genome-SP-21	CRISPR_IA unmapped_YMAAA02TF-SP-2
Both	OS-Only	Both	CRISPR_2	OS-A	CRISPR_IIE OS-A_Genome-SP-10	CRISPR_IIE OS-A_CYPDW52TF-SP-7
Both	OS-Only	Both	CRISPR_2	OS-A	CRISPR_IIE OS-A_Genome-SP-12	CRISPR_IIE OS-A_CYPDW52TF-SP-5
Both	OS-Only	Both	CRISPR_2	OS-A	CRISPR_IIE OS-A_Genome-SP-13	CRISPR_IIE OS-A_CYPDW52TF-SP-4
Both	OS-Only	Both	CRISPR_2	OS-A	CRISPR_IIE OS-A_Genome-SP-14	CRISPR_IIE OS-A_CYPDW52TF-SP-3
Both	OS-Only	Both	CRISPR_2	OS-A	CRISPR_IIE OS-A_Genome-SP-15	CRISPR_IIE OS-A_CYPDW52TF-SP-2
Both	OS-Only	Both	CRISPR_2	OS-A	CRISPR_IIE OS-A_Genome-SP-16	CRISPR_IIE OS-A_CYPDW52TF-SP-1
Meta-only	Both	Low-Only	CRISPR_2	OS-B	CRISPR_IIG OS-B'_YMIAX35TR-SP-1	CRISPR_IIG OS-B'_CYPIX94TF-SP-1
Meta-only	Both	Low-Only	CRISPR_1	OS-B	CRISPR_IB OS-B'_CYPDX40TR-SP-8	CRISPR_IB OS-B'_YMJA680TF-SP-5
Meta-only	Both	Low-Only	CRISPR_1	OS-B	CRISPR_IB OS-B'_CYPDX40TR-SP-9	CRISPR_IB OS-B'_YMJA680TF-SP-4
Meta-only	Both	Low-Only	CRISPR_1	OS-B	CRISPR_IB OS-B'_CYPDX40TR-SP-10	CRISPR_IB OS-B'_MJA680TF-SP-3
Meta-only	OS-Only	Low-Only	CRISPR_2	OS-B	CRISPR_IIA OS-B'_CYPKX54TF-SP-4	CRISPR_IIF OS-B'_CYPIP26TF-SP-4
Meta-only	OS-Only	Low-Only	CRISPR_2	OS-B	CRISPR_IIG OS-B'_CYPIE52TR-SP-2	CRISPR_IIG OS-B'_CYPIE52TR-SP-1
Meta-only	OS-Only	Low-Only	CRISPR_2	OS-B	CRISPR_IID OS-B'_CYPKD67TR-SP-3	CRISPR_IID OS-B'_CYPHA03TR-SP-7
Meta-only	OS-Only	Low-Only	CRISPR_2	OS-B	CRISPR_IID OS-B'_CYPKD67TR-SP-4	CRISPR_IID OS-B'_CYPHA03TR-SP-6
Meta-only	OS-Only	Low-Only	CRISPR_2	OS-B	CRISPR_IIF OS-B'_CYPC676TF-SP-1	CRISPR_II unmapped_CYPE874TF-SP-8
Meta-only	OS-Only	Low-Only	CRISPR_2	OS-B	CRISPR_IID OS-B'_CYPBU82TR-SP-5	CRISPR_II unmapped_CYPE874TF-SP-7
Meta-only	OS-Only	Low-Only	CRISPR_2	OS-B	CRISPR_IIF OS-B'_CYPBM48TF-SP-7	CRISPR_IIF OS-B'_CYPBM48TF-SP-6
Meta-only	OS-Only	Low-Only	CRISPR_2	OS-B	CRISPR_IIF OS-B'_CYPJ513TR-SP-10	CRISPR_IIF OS-B'_CYPAO83TF-SP-5
Meta-only	OS-Only	Low-Only	CRISPR_2	OS-B	CRISPR_IIG OS-B'_CYPHT46TF-SP-4	CRISPR_IIA OS-B'_CYPAC23TR-SP-6
Meta-only	OS-Only	Low-Only	CRISPR_2	OS-B	CRISPR_IIF OS-B'_CYOCJ70TF-SP-6	CRISPR_IIF OS-B'_CYOCB32TF-SP-9
Meta-only	OS-Only	Low-Only	CRISPR_2	OS-B	CRISPR_IIF OS-B'_CYOCJ70TF-SP-7	CRISPR_IIF OS-B'_CYOCB32TF-SP-8
Meta-only	OS-Only	Low-Only	CRISPR_2	OS-B	CRISPR_IIF OS-B'_CYOCJ70TF-SP-8	CRISPR_IIF OS-B'_CYOCB32TF-SP-7
Meta-only	OS-Only	Low-Only	CRISPR_2	OS-B	CRISPR_IIF OS-B'_CYOCJ70TF-SP-9	CRISPR_IIF OS-B'_CYOCB32TF-SP-6
Meta-only	OS-Only	Low-Only	CRISPR_2	OS-B	CRISPR_IIF OS-B'_CYOCJ70TF-SP-5	CRISPR_IIF OS-B'_CYOCB32TF-SP-10
Meta-only	OS-Only	Low-Only	CRISPR_2	OS-B	CRISPR_IIG OS-B'_CYPF753TF-SP-3	CRISPR_IIA OS-B'_CYOB470TR-SP-1
Meta-only	OS-Only	Low-Only	CRISPR_2	OS-B	CRISPR_IIA OS-B'_CYPDR90TR-SP-3	CRISPR_IIF OS-B'_CYOAX80TR-SP-4
Meta-only	OS-Only	Low-Only	Mix	OS-B	CRISPR_IB OS-B'_CYPDX40TR-SP-4	CRISPR_IIF OS-B'_CYOAX80TR-SP-3
Meta-only	OS-Only	Low-Only	Mix	OS-B	CRISPR_IB OS-B'_CYPD971TF-SP-3	CRISPR_IIF OS-B'_CYOAX80TF-SP-2        
Meta-only	OS-Only	Low-Only	CRISPR_1	OS-B	CRISPR_IB OS-B'_CYPLU89TR-SP-2	CRISPR_IB OS-B'_CYPLU89TR-SP-3
Meta-only	OS-Only	Low-Only	CRISPR_1	OS-B	CRISPR_IB OS-B'_CYPD971TF-SP-10	CRISPR_IB OS-B'_CYPD971TF-SP-7
Meta-only	OS-Only	Low-Only	CRISPR_1	OS-B	CRISPR_IB OS-B'_CYPBW78TR-SP-10	CRISPR_IB OS-B'_CYPBW78TR-SP-9
Genome	OS-Only	Low-Only	CRISPR_2	OS-B	CRISPR_IIA OS-B'_Genome-SP-4	CRISPR_IIA OS-B'_Genome-SP-3
Genome	OS-Only	Low-Only	CRISPR_2	OS-B	CRISPR_IIF OS-B'_Genome-SP-2	CRISPR_IIF OS-B'_Genome-SP-1
Both	Genome	Low-Only	Mix	OS-B	CRISPR_IID OS-B'_Genome-SP-4	CRISPR_IB OS-B'_CYPD001TR-SP-3
Both	OS-Only	Low-Only	CRISPR_2	OS-B	CRISPR_IIA OS-B'_Genome-SP-7	CRISPR_IIG OS-B'_CYPKE37TF-SP-1
Both	OS-Only	Low-Only	CRISPR_2	OS-B	CRISPR_IIA OS-B'_Genome-SP-15	CRISPR_IIA OS-B'_CYPAC23TR-SP-2
Both	OS-Only	Low-Only	CRISPR_2	OS-B	CRISPR_IIF OS-B'_Genome-SP-9	CRISPR_IIF OS-B'_CYOCJ70TR-SP-6
Meta-only	Both	Low-Only	CRISPR_2	Unknown	CRISPR_II unmapped_YMJAY30TR-SP-10	CRISPR_IIG OS-B'_CYPKE37TF-SP-7
Meta-only	Both	Low-Only	CRISPR_2	Unknown	CRISPR_II unmapped_YMIAS32TR-SP-2	CRISPR_IIG OS-B'_CYPAW81TR-SP-10
Meta-only	MS-Only	Low-Only	CRISPR_2	Unknown	CRISPR_II unmapped_YMIAS32TR-SP-3	CRISPR_II unmapped_YMIAS32TR-SP-1
Meta-only	OS-Only	Low-Only	CRISPR_2	Unknown	CRISPR_II unmapped_CYOC015TF-SP-5	CRISPR_IIA OS-B'_CYPKX69TF-SP-1
Meta-only	OS-Only	Low-Only	CRISPR_2	Unknown	CRISPR_II unmapped_CYOAE25TR-SP-4	CRISPR_IID OS-B'_CYPEK25TR-SP-5
Meta-only	OS-Only	Low-Only	CRISPR_2	Unknown	CRISPR_IIA OS-B'_CYPBM89TF-SP-9	CRISPR_IIA OS-B'_CYPBM89TF-SP-8
Meta-only	OS-Only	Low-Only	Mix	Unknown	CRISPR_IB OS-B'_CYPA321TF-SP-9	CRISPR_IIA OS-B'_CYPBM89TF-SP-5
Meta-only	OS-Only	Low-Only	CRISPR_2	Unknown	CRISPR_II unmapped_CYPAQ10TR-SP-6	CRISPR_II unmapped_CYPAQ10TR-SP-5
Meta-only	OS-Only	Low-Only	CRISPR_2	Unknown	CRISPR_IID OS-B'_CYPHA03TR-SP-8	CRISPR_II unmapped_CYOC015TR-SP-2
Meta-only	OS-Only	Low-Only	CRISPR_2	Unknown	CRISPR_IIA OS-B'_CYPBM89TF-SP-1	CRISPR_II unmapped_CYOAE25TR-SP-3
Meta-only	OS-Only	Low-Only	CRISPR_2	Unknown	CRISPR_IIA OS-B'_CYPBM89TF-SP-2	CRISPR_II unmapped_CYOAE25TR-SP-2
Meta-only	OS-Only	Low-Only	CRISPR_2	Unknown	CRISPR_IIA OS-B'_CYPBM89TF-SP-3	CRISPR_II unmapped_CYOAE25TR-SP-1
Meta-only	OS-Only	Low-Only	CRISPR_2	Unknown	CRISPR_IID OS-B'_CYPE216TR-SP-2	CRISPR_II unmapped_CYOAB48TR-SP-6
Meta-only	OS-Only	Low-Only	CRISPR_2	Unknown	CRISPR_IID OS-B'_CYPE216TR-SP-1	CRISPR_II unmapped_CYOAB48TR-SP-5
Meta-only	OS-Only	Low-Only	CRISPR_2	Unknown	CRISPR_II unmapped_CYPCN89TR-SP-9	CRISPR_II unmapped_CYOAB48TF-SP-4
Meta-only	OS-Only	Low-Only	CRISPR_2	Unknown	CRISPR_II unmapped_CYPCN89TR-SP-8	CRISPR_II unmapped_CYOAB48TF-SP-3
Meta-only	OS-Only	Low-Only	CRISPR_2	Unknown	CRISPR_II unmapped_CYPCN89TR-SP-7	CRISPR_II unmapped_CYOAB48TF-SP-2
Meta-only	MS-Only	High-Only	CRISPR_2	Unknown	CRISPR_II unmapped_YMBEQ85TF-SP-5	CRISPR_IIA OS-B'_YMACF06TF-SP-5
Meta-only	Both	Both	CRISPR_2	Unknown	CRISPR_IIF OS-B'_CYPBH35TR-SP-3	CRISPR_IIB OS-A_YMAAC54TF-SP-8
Meta-only	OS-Only	Both	CRISPR_2	Unknown	CRISPR_II unmapped_CYOAB48TR-SP-1	CRISPR_IID OS-B'_CYNAM11TR-SP-3
				6	3
				5	2
				4	4
				3	11
				2	104
				1	1300
